# Quantitative-enhancer-FACS-seq (QeFS) reveals epistatic interactions among motifs within transcriptional enhancers in developing *Drosophila* tissue

**DOI:** 10.1186/s13059-021-02574-x

**Published:** 2021-12-20

**Authors:** Colin T. Waters, Stephen S. Gisselbrecht, Yuliya A. Sytnikova, Tiziana M. Cafarelli, David E. Hill, Martha L. Bulyk

**Affiliations:** 1 Division of Genetics, Department of Medicine, Brigham and Women’s Hospital and Harvard Medical School, Boston, MA 02115 USA; 2 Program in Biological and Biomedical Sciences, Harvard University, Cambridge, MA 02138 USA; 3 Center for Cancer Systems Biology, Dana-Farber Cancer Institute, Boston, MA 02215 USA; 4 Department of Cancer Biology, Dana-Farber Cancer Institute, Boston, MA 02215 USA; 5 Department of Genetics, Blavatnik Institute, Harvard Medical School, Boston, MA 02115 USA; 6 Department of Pathology, Brigham and Women’s Hospital and Harvard Medical School, Boston, MA 02115 USA

**Keywords:** Enhancers, Transcription factor binding sites, Epistasis, *Drosophila* embryonic mesoderm, Reporter assay

## Abstract

Understanding the contributions of transcription factor DNA binding sites to transcriptional enhancers is a significant challenge. We developed Quantitative enhancer-FACS-Seq for highly parallel quantification of enhancer activities from a genomically integrated reporter in *Drosophila melanogaster* embryos. We investigate the contributions of the DNA binding motifs of four poorly characterized TFs to the activities of twelve embryonic mesodermal enhancers. We measure quantitative changes in enhancer activity and discover a range of epistatic interactions among the motifs, both synergistic and alleviating. We find that understanding the regulatory consequences of TF binding motifs requires that they be investigated in combination across enhancer contexts.

**Supplementary Information:**

The online version contains supplementary material available at 10.1186/s13059-021-02574-x.

## Background

Identifying sequence features of *cis*-regulatory elements that are important for their activities is a key challenge in understanding how regulatory information is encoded in genomes. Computational analysis of transcriptional enhancers with overlapping tissue and temporal expression patterns for enrichment of sequence matches to transcription factor (TF) DNA binding site motifs can aid in the identification of TFs that contribute to the enhancers’ activities [1, 2]. Numerous studies have investigated the contributions of individual motifs to enhancer activity [3–6]. In many cases, combinatorial regulation by multiple TFs is required to specify an appropriate expression pattern, and the contribution of a given motif instance to transcriptional regulation is sensitive to context. This complicates the interpretation of motif enrichment. Experimental analysis of enhancers in which motif occurrences have been mutated in various combinations is needed to determine the contributions of the motifs to the activities of the enhancers and whether a motif exerts different effects depending on the enhancer context [7, 8].

Various genomic sequence features surrounding a TF binding site can present a (dis)favorable context for binding by the corresponding TF. Local sequence context can affect the probability of TF binding by various mechanisms, such as by providing a flanking sequence that adopts a DNA shape preferred by a particular TF [9], contains a motif that is preferential for co-binding with a protein partner(s) [10–12], contains a motif bound by a TF that affects local chromatin accessibility [13–15], contains a motif for competitive binding by a different TF [16, 17], or corresponds to sequence disfavorable for nucleosomes [18]. The potential for motifs present within enhancers to influence the activity of nearby motifs is referred to as epistasis or genetic interaction. Such epistatic interactions, where the regulatory output of a particular combination of motifs is different from that expected if the motifs are treated as acting independently, are important to identify: rules learned for individual motifs in one enhancer context might not be informative of those motifs’ regulatory consequences in a different enhancer context. In a study of human TF binding sites, linear regression models that incorporated pairwise interactions between motifs in 145-bp sequences explained a higher fraction of the variance in activation of reporter expression, with five motif pairs showing significant interactions with regard to activity of both genomic and synthetic enhancer sequences [19]. A challenge for investigations to identify context-specific effects of motifs on enhancer activity is the need for quantitative data on wildtype enhancers and enhancers mutated for particular motifs across a panel of different enhancer sequence contexts.

Enabled by advances in high-throughput DNA sequencing and oligonucleotide synthesis, various technologies have been developed for highly parallel analysis of genomic or synthetic sequences for promoter or enhancer activity in a reporter assay [18, 20]. Massively parallel reporter assays (MPRAs) [21] and STARR-Seq [22] are powerful in their ability to assay tens to hundreds of thousands of short elements (typically less than 200 bp in length) for their ability to drive expression of a reporter in cultured cells. While these assays have allowed the identification of nucleotides within enhancers that are important for their *cis*-regulatory activity, they have a number of limitations. First, they employ episomal reporter constructs, but the activities of chromosomally integrated sequences can differ substantially from their activities when tested in episomes [23]. Furthermore, not all cell types are well modeled by cell lines, and the activities of some tissue-specific enhancers may not be cell autonomous. While MPRAs have been performed in retinal explants [24–26] and in mouse liver [27], there is a need to assay enhancers in additional tissues in the context of whole organisms, such as for tissue-specific developmental enhancers active in embryos [28, 29].

The enhancer-FACS-seq (eFS) technology addresses these needs by using (1) a genomically integrated enhancer-reporter system in live, developing animals; (2) cell-type-specific-expression of an ectopic cell-surface marker to isolate a cell population of interest; and (3) analysis of reporter gene expression driven from a site-specific integration site with low background activity. This technology is limited in throughput by the efficiency of *Drosophila* transgenesis, which permits the use of lower-throughput library construction methods that test the activity of longer sequences, making it a complement to higher-throughput episomal methods. eFS analysis of a pool of candidate enhancers identified by various criteria, including enrichment for occupancy by mesodermal TFs or clustering of conserved TF binding motifs, validated 12 known and identified 45 novel enhancers active in *Drosophila* embryonic mesoderm [1].

Motif enrichment and pairwise motif analysis of these mesodermal enhancers revealed enrichment of the DNA binding specificity motifs of TFs with a known role in mesoderm development and also of factors not previously known to have a role in transcriptional regulation in mesoderm [1]. These putative novel mesodermal regulatory motifs included those corresponding to the factors Deaf1, Schlank, ZIPIC, and CG12236­PB. Best characterized of these four factors is Deaf1, which was previously identified as a cofactor for the homeotic TF Deformed (Dfd) [30] and mutants of which led to embryonic segmentation defects [31]. Both repressive and activating activities of Deaf1 have been reported in different cell types [32], suggesting Deaf1 may exhibit cell type-specific activity, but its overall regulatory activity remains poorly understood. ZIPIC has been characterized as a putative insulator protein that physically interacts with CP190 [33] and was subsequently found to bind topologically associated domain (TAD) boundaries [34], supporting its putative role as an insulator. Schlank was initially characterized as a transmembrane protein with a role in fatty acid synthesis [35]; intriguingly, its homeodomain has been shown to play a role in fatty acid homeostasis independent of its enzymatic activity [36]. CG12236-PB has not been investigated beyond the identification of its DNA binding motif through a bacterial one-hybrid screen [37]. All of these factors have maternally deposited mRNA and ubiquitous expression early in embryogenesis [38], which may have contributed to their failure to be characterized by classical *Drosophila* genetics.

We sought to develop a method that would allow us to uncover the context-dependent, combinatorial impact of the DNA binding motifs of these four TFs on tissue-specific activities of the mesodermal enhancers that we had identified by eFS. To address this need, here we have developed Quantitative enhancer-FACS-Seq (QeFS) technology, which provides an RNA-Seq-based readout of enhancer activity. QeFS thus provides a quantitative readout of enhancer activity, allowing sensitive measurement of the effects of loss of individual or combinations of motifs within an enhancer on its activity. Comparisons of QeFS data among a series of enhancer variants enabled us not only to quantify the contributions of individual motifs but also to identify epistatic interactions between motifs through the analysis of combinatorially mutated enhancers. Such sensitive, quantitative readout is crucial for understanding the activity of factors that have subtle effects on relative gene expression levels. We attempted QeFS in whole mesoderm and separately in fusion competent myoblasts (FCMs), a small subset of the mesoderm, in developing *Drosophila* embryos to study the roles of the DNA binding motifs recognized by the TFs Deaf1, Schlank, ZIPIC, and CG12236­PB on mesodermal enhancer activity and to identify epistatic interactions among these motifs.

## Results

### Quantitative measurement of the contributions of TF DNA binding motifs to enhancer activity in embryos

**Fig. 1:**
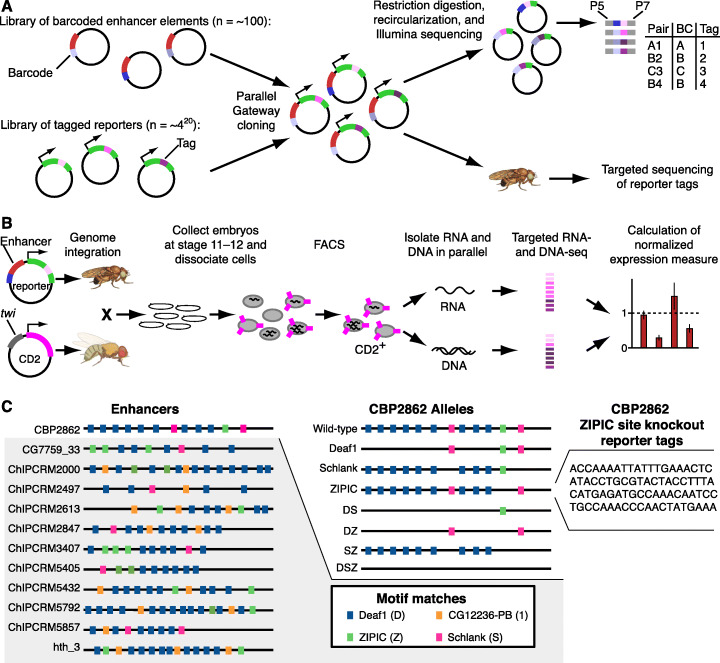
Strategy of the QeFS experiment. **A** Barcoded enhancers were cloned into reporter constructs containing degenerate 3′ tags. A sequencing-based approach (see the “Methods” section) associates tags with barcodes (“BC”). P5, P7: Illumina adapters. **B** A pool of reporter flies was generated and crossed to a marker line enabling FACS isolation of the desired cell types. Targeted RNA- and DNA-sequencing of degenerate tags measured reporter and transcript abundance; DNA-normalized enhancer expression levels were calculated and used for subsequent analysis. **C** Schematic of enhancer library design. *(Left)* The 12 endogenous enhancers and relative locations of motifs selected for perturbation are displayed in the left panel. *(Middle)* An example mutational series is shown for the enhancer CBP2862, including the wildtype, single motif mutants, double mutants and the triple mutant, comprising mutations in the DNA binding motifs of Deaf1, Schlank, and ZIPIC. These enhancers otherwise retain their endogenous sequence context, and other TF binding motifs are maintained in the sequence. *(Right)* Each enhancer construct is represented by multiple tags in the reporter library, providing internal biological replicates

To assay *cis*-regulatory elements for enhancer activity in a quantitative, highly parallel fashion in a chromosomal context in the context of particular tissues in whole *Drosophila* embryos, we developed quantitative enhancer-FACS-Seq (QeFS). In QeFS, each *cis*-regulatory element was cloned upstream of a reporter gene, in whose 3′ UTR a degenerate sequence was inserted and used as an identifying tag in RNA-Seq readout of enhancer activity (Additional file 1: Fig. S1, S2, S3). To simplify the process by which highly similar *cis*-regulatory elements were distinguished, we barcoded the elements. Briefly, we synthesized a library of wildtype and mutant enhancers with flanking 8-mer barcodes and cloned them into a reporter backbone, with each reporter containing a unique, degenerate 20-mer tag in the 3′ UTR of the reporter transcript (Fig. 1A). Sequence-validated reporter constructs were pooled to create a library of 303 unique enhancer-reporter constructs. We used a custom high-throughput sequencing library preparation protocol involving digestion, re-circularization, and sequencing [27] to generate a lookup table that associates reporter tags to the corresponding enhancer barcodes (Additional file 1: Fig. S4, S5, S6, S7). Typically at least 3 different tags were associated with each enhancer (Additional file 2: Table S1; Additional file 1: Fig. S8).

Next, as in eFS [1], we injected the enhancer-reporter library into *Drosophila* embryos, resulting in genomic integration of individual enhancer-reporter elements at a fixed landing site in each haploid genome of an embryo’s germ cell progenitors. Assaying all the wildtype and mutant enhancer constructs from the same chromosomal integration site allowed us to determine the effects of a particular mutation or combination of mutations on the activity of an enhancer. Following a series of fly crosses, a population of flies was obtained, each with a single enhancer-reporter construct present in the genome of every cell in the adult fly. We marked our cell types of interest by crossing this population to a fly line expressing the rat cell surface marker CD2 under the control of a cell type-specific enhancer (either *twi* for whole mesoderm or *Mef2-I-E*_*D5*_ for FCMs) (Fig. 1B). Embryos from this cross were aged to the desired stage (i.e., stages 11–12), harvested, and used to prepare single-cell suspensions. These suspensions were then subjected to FACS for the CD2 marker, to purify our cell types of interest (Additional file 1: Fig. S9; Additional file 2: Table S2). The small numbers (generally less than 10^5^; see Additional file 2: Table S2) of cells recovered from the more specific *mef2*:CD2+ population gave information on very few tested elements after filtering to remove unreliable measurements (see the “Methods” section), establishing the limitations of the method for rare cell types at this scale. The complementary *mef2*:CD2- population was more reliably measured and provided a proxy for whole embryo measurements, as very few cells are excluded.

We processed the collected cell populations (CD2+ and CD2-) to prepare RNA-seq and DNA-seq libraries in parallel from the same collection of informative cells, representing the reporter tag in the 3′ UTR of the reporter transcript, or the reporter tag in the 3′ UTR of the reporter DNA template, respectively (Additional file 1: Fig. S10, S11). We used a unique molecular identifier (UMI) strategy to count individual molecules containing each tag, assigned tag counts to enhancers using the lookup table, and normalized the tag counts in the RNA-seq library to those in the DNA-seq library, thus scaling reporter transcript levels to the underlying abundance of individual reporters in the fly population (Additional file 1: Fig. S12, S13).

In order to test this experimental strategy, we designed a library of wildtype and mutant enhancers intended to take advantage of the strengths of this approach, including the resulting quantitative reporter data. We previously found that the DNA binding site motifs of the ubiquitously expressed [39] TFs Deaf1, CG7928 (ZIPIC), CG12236-PB, and Schlank were enriched among mesodermally active enhancers [1]. Although these TFs were not known to regulate embryonic mesoderm development, the enrichment of their recognition motifs both individually and together with each other and with master regulators in either whole mesoderm or fusion-competent myoblasts (FCMs), an abundant mesodermal subpopulation, suggested that they may contribute to mesodermal enhancer activity. To investigate the roles of these motifs in mesodermal enhancer activity, we selected a panel of 12 endogenous enhancers of approximately 1 kb in length that are active in embryonic mesoderm during developmental stages 11–12 [1] and that each contain matches to three of these four motifs. To allow us to determine the contributions of each of the Deaf1, CG7928 (ZIPIC), CG12236-PB, and Schlank motifs to enhancer activity, we designed a series of systematic single-motif mutations, whereby in each enhancer we mutated all the sites matching each of these motifs, considering each motif one at a time (“single-motif mutants”). Additionally, to identify potential regulatory interactions between these motifs, we designed combinatorial enhancer mutants comprising mutations in all possible pairs and three-way combinations of these motif mutants; thus, for each of the twelve enhancers, we synthesized eight different versions: wildtype, three single-motif mutants, three pairwise mutants, and one triple mutant (Fig. 1C, Additional file 3: Table S3). Mutant enhancers differed from wildtype by as little as 1 nucleotide out of ~ 1000, with a mean of 7.2 nucleotides mutated per enhancer. In total, we collected QeFS data for 88 wildtype or mutant enhancers and 5 spiked-in control elements (2 wildtype and 3 mutants) described below. Normalized reporter expression data generally correlated well across pairs of tags measuring the activity of the same enhancer construct in a given collection (Spearman’s *ρ* = 0.786); enhancer construct activities were calculated as the mean normalized value across tags across replicate QeFS experiments.

As positive controls to assess the sensitivity and cell-type specificity of QeFS, we analyzed two enhancers known to exhibit mesoderm-specific activity during embryonic stages 11–12 and previously found to be sensitive to either a single or a set of motif mutations. One element, for *Actin 57B*, showed early visceral mesoderm activity, spreading to whole mesoderm by stage 12 of development [40]. This element was reported to be very sensitive to loss of a single binding site for the TF Mef2, with 87% reduction in *lacZ* reporter activity [40]. The second enhancer, for *β3Tub60D*, had been reported to be active in developing visceral mesoderm [41]. Imaging experiments using *lacZ* reporters revealed that this element was moderately sensitive to a single site mutation for the TF bagpipe (bap), while combinatorial mutations in two biniou (bin) sites and this single bap site led to complete loss of enhancer activity [41]*.* As previously described, both enhancers' activities are restricted to the mesoderm, and none of the selected site mutations caused any detectable non-mesodermal expression.

We cloned each of these wildtype and mutant enhancers—wildtype and Mef2 site mutant *Actin 57B* enhancer, and wildtype, bap site mutant, and triple mutant *β3Tub60D* enhancers—barcoded them, and spiked them into our enhancer library (see the “Methods” section; Additional file 1: Fig. S14; Additional file 3: Table S3). In parallel, we also created homozygous fly strains carrying reporter constructs for each of these five elements; embryos from each of these individual homozygous strains were harvested at developmental stages 11–12 for analysis of enhancer activity by imaging (Additional file 1: Fig. S15) to confirm the published effects of each mutation or by RT-qPCR.

**Fig. 2:**
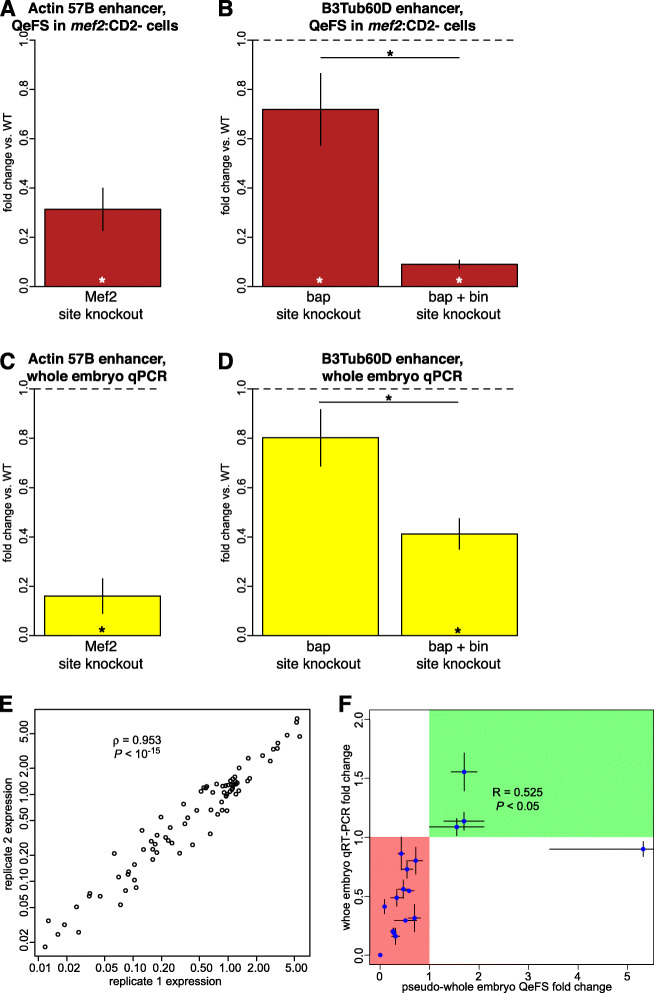
QeFS provides quantitative enhancer activity data consistent with RT-qPCR data. **A**, **B** QeFS measurements recapitulate the quantitative effects of previously published binding site knockouts in two different mesodermal enhancers. Asterisks within bars represent statistically significant differences (adjusted *P*-value < 0.05, Conover-Iman post-hoc test with Benjamini-Hochberg correction) from wildtype enhancer activity; asterisks with horizontal lines indicate significant pairwise differences between mutant constructs. **C**, **D** Reporter gene expression measured by RT-qPCR from whole embryo RNA in biological triplicates is generally concordant with QeFS measurements. Significance assessed by paired-sample t-test of Cq values. **E** Reproducibility of QeFS data. QeFS expression levels from week 1 were highly correlated with those from week 2 for enhancers in the *mef2:*CD2- context (Spearman’s *ρ* = 0.95). **F** Validation of QeFS data. Effects of mutations on QeFS-measured expression levels of enhancers in *mef2:*CD2- cells were correlated with effects of the same mutations measured by whole embryo qRT-PCR in biological triplicates (Pearson’s *R* = 0.525). Green quadrant represents mutants for which both methods found increased expression; red quadrant highlights mutants for which both methods found decreased expression relative to wild type. Error bars in all panels show s.e.m. of measured fold change

Analysis of these control enhancers revealed high concordance of enhancer activity measured by QeFS from the spike-in constructs in *mef2*:CD2- cells (Fig. 2A,B) as a proxy for measurements from whole embryos, with that determined by RT-qPCR (Fig. 2C,D) from whole embryos from the homozygous strains. These reporter assay data of enhancer activity were consistent with imaging results of the activities of these enhancers [40, 41]. For example, the single Mef2 site mutation in the *Actin 57B* enhancer led to a significant decrease in enhancer activity by QeFS and of similar magnitude as that measured by RT-qPCR (*P* = 0.0007, Welch’s two-sample, one-tailed *t*-test). Similarly, the single Bap site mutation in the *β3Tub60D* enhancer led to a detectable decrease in enhancer activity by both QeFS (*P* = 0.0258) and RT-qPCR (*P* > 0.05), while the Bin+bap triple site mutant led to a significant decrease in enhancer activity by both QeFS and RT-qPCR versus both the wildtype enhancer (*P* < 0.0001, Conover-Iman post-hoc test with Benjamini-Hochberg correction) and the single mutant (*P* = 0.0001).

To assess the reproducibility of QeFS, we compared the mean measured expression level (normalized by input abundance) across all tested elements between two independent collections of *mef2*:CD2- material. The reporter transcript levels between the two experiments were highly reproducible (Spearman’s ρ = 0.953, *P* < 10^−15^) (Fig. 2E). We also compared the results of QeFS from *mef2*:CD2- cells to RT-qPCR measurement on whole embryos for 14 additional mutant constructs recovered at random or selected for replication (Fig. 2F). Fold change measurements vs. wild type were significantly correlated by both methods (Pearson's R = 0.525, *P* < 0.05) and concordant in 16/17 cases. Imaging of antibody-stained embryos for the one discordant case (Additional file 1: Fig. S16) confirmed that it is a false positive of QeFS; all other validations by imaging broadly supported the QeFS measurements (Additional file 1: Fig. S17, S18, S19, S20).

Altogether, these results demonstrate that QeFS enables highly accurate determination of the activities of *cis*-regulatory elements from a fixed, chromosomal context in *Drosophila*, allowing comparisons of the effects of TF binding site mutations on enhancer activity.

### Loss of Deaf1 or CG12236-PB sites causes context-dependent reduction in mesodermal enhancer activity

**Fig. 3:**
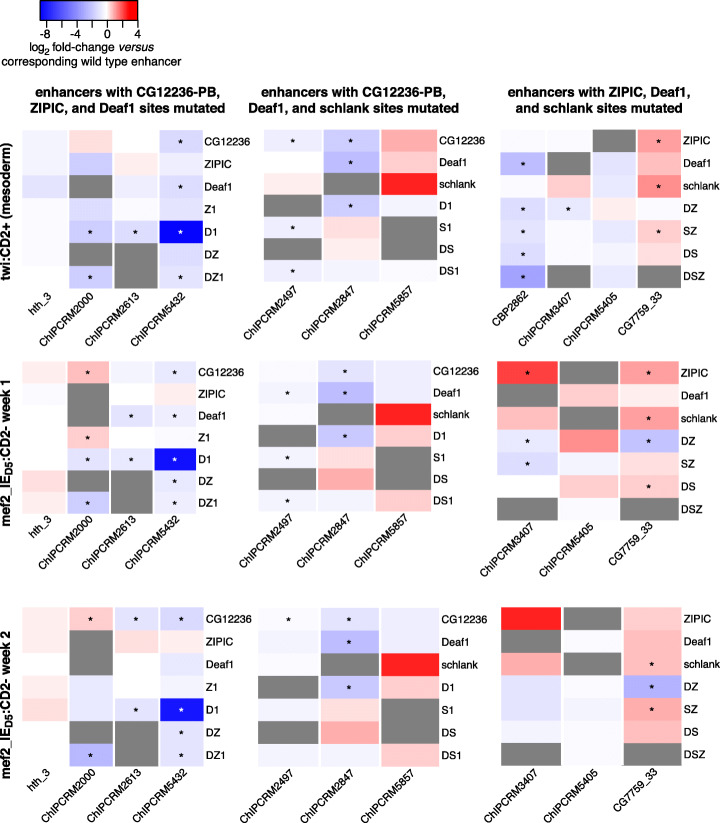
Fold change of mutant enhancer activity relative to wildtype in separate experiments. Fold change for each enhancer construct relative to wildtype is indicated by the heatmap color for each mutant construct and each enhancer. Red indicates increased enhancer activity caused by site mutations and blue indicates decreased activity. Gray boxes represent measurements which did not pass quality control filters. “D1” is the Deaf1 and CG12236-PB combinatorial mutant, “SZ” is the Schlank and ZIPIC combinatorial mutant, etc., while enhancer context is indicated at the bottom of each heatmap. Significant differences from wildtype (FDR < 0.1, Conover-Iman test with Benjamini-Hochberg correction for multiple hypothesis testing) are indicated with an asterisk. Enhancers are grouped by column according to the three motifs mutated within each allelic series; each row represents results from a separate population of parental flies and triplicate FACS collections. ChIPCRM5792-WT was not reliably detected, so that fold change measurements cannot be displayed for this enhancer

**Fig. 4:**
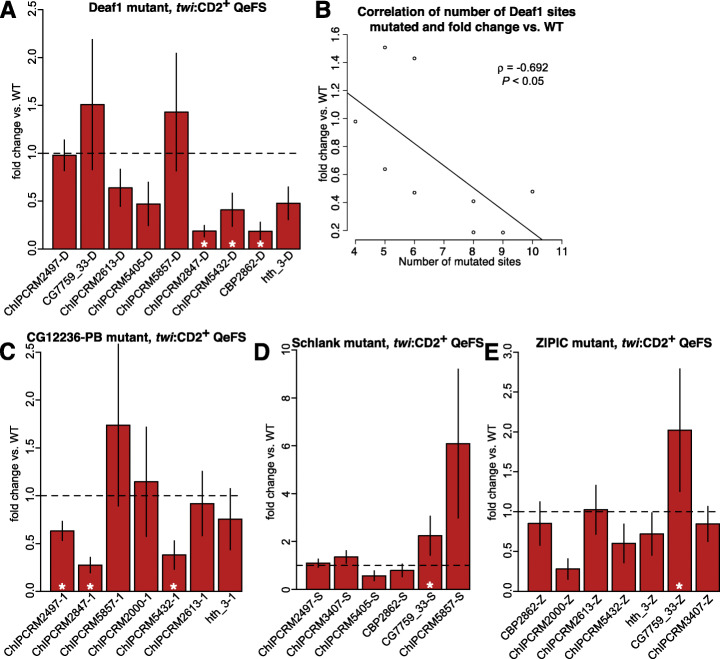
Mutation of Deaf1, Schlank, CG12236-PB, or ZIPIC binding sites affects enhancer activity in mesodermal cells. **A** Loss of enhancer activity due to Deaf1 site mutations in 3/9 mesodermal enhancers. Enhancers are ordered by increasing number of Deaf1 sites mutated. **B** Reduction in enhancer activity is correlated (Spearman's rho = − 0.69, *P* = 0.039) with the number of mutated sites in a given enhancer. **C** Loss of enhancer activity due to CG12236-PB site mutations in 3/7 mesodermal enhancers. **D** Schlank site mutations in CG7759_33 led to significantly increased enhancer activity. **E** ZIPIC site mutation in CG7759_33 led to significantly increased enhancer activity. Asterisks and error bars are as in Fig. 2 Binding site abbreviations are as shown in Fig. 1

To determine the contribution of the Deaf1, ZIPIC, CG12236-PB, and Schlank DNA binding site motifs to each enhancer’s activity in each of two different tissue types (whole mesoderm, FCMs), we compared the normalized RNA-Seq level of each mutant enhancer to that of the corresponding wildtype enhancer in each tissue. We then inspected the resulting QeFS fold change values for trends in motif activity across the analyzed panel of enhancers (Fig. 3; Additional file 4: Table S4). We observed that loss of Deaf1 sites led to decreased enhancer activity in whole mesoderm when it had any reliably detectable effect, with significant reduction in 3/9 enhancers (Fig. 4A; Additional file 1: Fig. S21, S22, S23, S24, S25, S26, S27, S28, S29), suggesting that Deaf1 generally acts as an activator in mesodermal cells at stages 11–12. Notably, different enhancers were differentially sensitive to Deaf1 binding site mutations. Five of the 12 enhancers were sensitive to mutation of Deaf1 sites in at least one cell type. For example, Deaf1 site mutation within the ChIPCRM2847 enhancer led to a significant decrease in activity in whole mesoderm (*P* < 0.0001) (Fig. 4A) and in non-FCM cells (*P* < 0.0001) (Fig. 3), which approximate whole embryo as described above. In other cases, the effect was tissue-specific; for example, Deaf1 site mutation in ChIPCRM2497 resulted in loss of enhancer activity only in non-FCM cells (*P* = 0.0079). In two enhancers (ChIPCRM5857 and CG7759_33), Deaf1 site mutations led to a measurable, but not statistically significant, increase in enhancer activity in whole mesoderm (Fig. 4A). This variability in response level and direction is consistent with a prior analysis of Deaf1 activity at a single promoter region, in which single site knockouts led to variable effects on reporter expression, with the strongest effect being loss of activity following site mutation [42], as we observed here. Interestingly, in whole mesoderm, there was a correlation (Spearman’s *ρ* = − 0.692, *P* < 0.05) between the number of sites mutated in a given enhancer and the overall reduction in enhancer activity, suggesting that homotypic clustering of Deaf1 binding sites within an enhancer contributes to larger increases in mesodermal enhancer activity (Fig. 4B).

Mutation of CG12236-PB sites also tended to reduce enhancer activity, with 3/7 tested enhancers showing significant reduction in whole mesoderm (Fig. 4C; Additional file 1: Fig. S21, S23, S25, S26, S27, S29, S30). For example, ChIPCRM2847 activity in mesodermal cells decreased upon mutation of CG12236-PB sites (*P* < 0.0001). To our knowledge, a functional activity for CG12236-PB had not been demonstrated previously. Our data suggest that CG12236-PB is a novel, sequence-specific transcriptional activator in embryonic mesoderm development.

### Effects of loss of ZIPIC or Schlank binding sites are highly context-dependent

In contrast to reductions in enhancer activity due to Deaf1 or CG12236-PB binding site mutation, the QeFS data revealed few consistent changes in enhancer activity resulting from mutation of ZIPIC or Schlank binding sites alone. We found that Schlank binding site mutations in CG7759_33 led to a significant increase in enhancer activity in whole mesoderm (*P* = 0.0048) (Fig. 4D; Additional file 1: Fig. S21, S22, S24, S25, S28, S31), consistent with a prior report that Schlank contributes to transcriptional repression of the *Drosophila* gene *lip3* [36]. A significant increase in enhancer activity was also observed for ZIPIC mutations in CG7759_33 in mesodermal cells (*P* = 0.0099) (Fig. 4E; Additional file 1: Fig. S22, S23, S27, S28, S29, S30, S31) and in ChIPCRM3407 in non-FCM cells (*P* = 0.0087) (Fig. 3). Where changes in expression are detected upon mutation of ZIPIC sites alone, they do not appear to reflect changes in the timing or pattern of expression (Additional file 1: Fig. S16, S19), suggesting an effect on the per-cell expression level.

### QeFS reveals epistatic interactions among sites within enhancers

Bolstered by the ability of QeFS to determine the effects of mutations of an individual TF’s binding sites within enhancers, we next analyzed the QeFS data for evidence of potential regulatory dependencies (also known as genetic interactions, or epistatic interactions) among motifs within the enhancers. Briefly, we examined our data for “synergistic” genetic interactions, where mutation of two motifs results in a greater change in reporter gene expression than is predicted from the product of the changes observed upon mutation of sites of the individual motifs [43], and for “alleviating” (also termed “antagonistic”) genetic interactions, where mutation of two motifs results in a less severe change in reporter gene expression than is predicted from the product of the changes observed upon mutation of sites of the individual motifs [44]. Among our panel of combinations of motif mutations across these 12 mesodermal enhancers, we observed cases of both synergistic and alleviating genetic interactions. Notably, all of the epistatic interactions that we observed between motifs were dependent on the enhancer context.

**Fig. 5:**
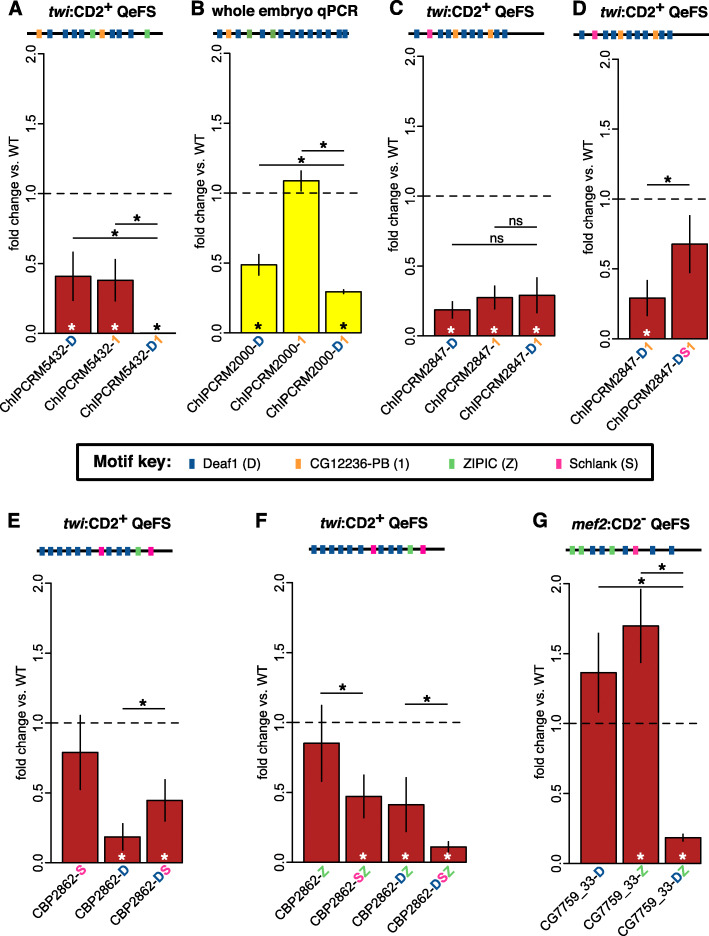
Effects of combinatorial motif mutations on enhancer activity. **A** Mutating Deaf1 and CG12236-PB sites together in the ChIPCRM5432 enhancer reduces activity more than predicted under a multiplicative model. **B** A similar epistatic interaction between these two classes of binding site is observed by whole embryo RT-qPCR in the ChIPCRM2000 enhancer. **C** Mutating Deaf1 and CG12236-PB sites together in the ChIPCRM2847 enhancer causes no additional reduction in activity as compared to mutating either class of site alone. **D** Schlank site mutation reduces the effect of mutating Deaf1 and CG12236-PB sites in this enhancer. **E** Mutating Schlank sites in the CBP2862 enhancer increases expression in the context of Deaf1 site mutations but not in the wild type enhancer. **F** Mutating Schlank sites decreases expression from the CBP2862 enhancer when the single ZIPIC site is also mutated. **G** Mutating Deaf1 and ZIPIC sites in the CG7759_33 enhancer reduces its activity, although neither class of site mutation reduces its activity alone. Asterisks and error bars are as in Figs. 2 and 3; binding site abbreviations are as in Fig. 1

In *twi*:CD2+ cells, where mutation of the 8 Deaf1 sites or of a single CG12236-PB site in the ChIPCRM5432 enhancer both led to a significant decrease in expression relative to the wildtype enhancer (*P* = 0.0147 or 0.0148, respectively) (Fig. 5A, Additional file 1: Fig. S27), mutation of both the Deaf1 and CG12236-PB sites resulted in a synergistic reduction in enhancer activity (*P* < 10^−15^). Like the effects of ZIPIC mutations described above, this epistasis appears to occur uniformly in all the cells in which this enhancer drives expression (Additional file 1: Fig. S18). We examined the other enhancers in which Deaf1 and CG12236-PB sites were mutated (Fig. 3) to see if this synergy was observed in other contexts. As QeFS data were not available for ChIPCRM2000-D, we generated transformant lines and observed synergistic effects between these two motifs in ChIPCRM2000 by whole-embryo qRT-PCR (Fig. 5B) and by imaging of stained embryos (Additional file 1: Fig. S17); mutating a single CG12236-PB site in this enhancer caused a significant reduction of activity (*P* = 0.0382) in the context of Deaf1 site knockouts but not on its own. In contrast, mutation of either Deaf1 or CG12236-PB sites in ChIPCRM2847 reduced its enhancer activity in mesodermal cells by over 70% (*P* < 0.0001), but mutation of sites for both motifs in that enhancer had no greater effect than mutation of sites for either factor alone (*P* > 0.2, Kruskal-Wallis rank sum test with Benjamini-Hochberg correction for multiple hypothesis testing) (Fig. 5C, Additional file 1: Fig. S26), suggesting that the regulatory outputs of CG12236-PB and Deaf1 for this enhancer are dependent on each other. These results demonstrate a range of context-dependent interactions between Deaf1 and CG12236-PB sites in mesodermal enhancers.

The reduction of ChIPCRM2847 enhancer activity resulting from mutation of Deaf1 and/or CG12236-PB sites was significantly abated (*P* < 0.002) when they were mutated in combination with mutation of a Schlank site (Fig. 5D, Additional file 1: Fig. S26). This result is consistent with Schlank acting primarily as a transcriptional repressor in the mesoderm, as we observed above for the CG7759_33 enhancer. Unlike other mutations for which we generated imaging data, the mutation of a Schlank site in this context appears to have somewhat cell type-specific effects, rescuing expression in only a small subset of the cells in which the wild type enhancer is active (Additional file 1: Fig. S20).

In addition to pairwise epistatic effects, we also observed higher-order interactions (Additional file 4: Table S4), such as in the case of the CBP2862 mutation series. For example, we observed an alleviating epistatic interaction (*P* = 0.017) between Schlank and Deaf1 site mutations. Even though mutation of Schlank sites on their own did not increase activity of the enhancer CBP2862, Schlank site mutations partially rescued (*P* = 0.0062) the diminished enhancer activity (*P* = 0.0001) that arose from Deaf1 site mutations in the CBP2862 enhancer (Fig. 5E, Additional file 1: Fig. S28). In contrast, when the single ZIPIC site was also mutated in this enhancer, mutation of Schlank sites decreased enhancer activity (Fig. 5F, Additional file 1: Fig. S28). The Schlank+ZIPIC double site knockout element drove significantly lower expression than did the ZIPIC alone knockout (*P* = 0.029), and the Deaf1+Schlank+ZIPIC triple site knockout also showed significantly reduced activity relative to the Deaf1+ZIPIC double knockout (*P* = 0.0169). These results demonstrate an example of “sign epistasis,” where the effect of mutating a motif on its own was in the opposite direction of the effect when it was mutated together with another motif, here suggesting that in the context of ZIPIC site mutations, Schlank can act as an activator. Such context-dependent activity of Schlank may be due to altered protein-protein interactions induced by the presence of ZIPIC. Context-dependent activity of a TF as either an activator or a repressor has been observed previously in *Drosophila* [45].

Finally, we also observed sign epistasis between Deaf1 and ZIPIC motifs. Mutation of ZIPIC sites alone in the CG7759_33 enhancer resulted in increased expression in non-FCM cells (*P* = 0.0005), while mutation of Deaf1 sites in CG7759_33 did not exert a statistically significant effect. Mutating Deaf1 and ZIPIC sites in combination, however, resulted in strongly reduced enhancer activity (*P* < 0.0001) (Fig. 5G, Additional file 1: Fig. S32).

## Discussion

In this study, we developed and applied QeFS to assay the effects of multiple TF binding site mutations on enhancer activity in the developing mesoderm in *Drosophila* embryos. QeFS provides: (1) a direct, quantitative read-out of enhancer activity by sequencing reporter transcripts; (2) a barcoding and tagging strategy that enables discrimination of reporter transcripts driven by very similar (> 99% sequence similarity) enhancer elements; (3) multiple distinct reporter constructs per enhancer element, enabling analysis of variance-based comparisons of activity driven by enhancers within a given mutation series; and (4) the use of UMI tagging of reporter constructs, reducing biases introduced during library preparation steps. As with the prior eFS technology [1], QeFS allows for parallel assays of the activities of hundreds of genomically integrated tissue-specific enhancers in the context of a whole organism. It thus provides a compromise approach between the extreme throughput of MPRAs carried out with episomal reporters in cultured cells and the exquisitely detailed information available from conventional reporter assays, for which informative transgenic lines must be made individually.

Our enhancer barcoding approach enabled the analysis of pools of enhancers that varied at only a few key nucleotide positions, and our degenerate reporter tagging strategy allowed us to use a non-parametric analysis-of-variance statistical approach to distinguish the activity level driven by different enhancers in the same cell type and developmental stage. This approach could be applied to any cell type in *Drosophila* which can be collected in sufficient numbers and for which a well-defined, cell and developmental stage-specific enhancer is available to drive CD2 expression. Additionally, this approach could be extended to other model organisms for which tools for genomic integration are available [46, 47].

The quantitative nature of the QeFS data is the key advance over the prior eFS technology. QeFS allows the identification of not just varying degrees of decreased enhancer activity, but also increased enhancer activity. The library of enhancer elements that we analyzed in this study was specifically designed to assess the contributions of the DNA binding site motifs of four TFs—Deaf1, Schlank, ZIPIC, and CG12236-PB—to the activities of 12 mesodermally active enhancers. Notably, all 4 motifs that we investigated in this study are those of poorly characterized regulators. Most TFs that have strong effects have in all likelihood already been identified from earlier genetic studies and screens in the literature; many poorly characterized TFs remain, and they likely are regulators that exert effects that are subtle and/or highly context-dependent in a manner that depends on the particular enhancer sequence and/or the particular tissue or cell type in which activity is being assessed. Examining the effects of these motifs in combination and in the context of several different enhancer sequences (all assayed at a common genomic location) in the *Drosophila* mesoderm permitted the detection of such subtle effects.

Our results provide insights into the roles of Deaf1, Schlank, ZIPIC, and CG12236-PB DNA binding site motifs in mesodermal enhancer activity. The reductions in enhancer activity that we observed upon mutation of Deaf1 or CG12236-PB binding site motifs suggest that they act as activators in mesoderm. These results are consistent with a prior study that found decreased reporter activity in the *Drosophila* SL2 cell line from a Deaf1-dependent promoter that was mutated for a Deaf1 binding site [42]. In contrast, the increases in enhancer activity that we observed upon mutation of Schlank or ZIPIC binding sites suggest that they act as repressors in mesoderm. Our results support a previous study that found Schlank to act as a transcriptional repressor in *Drosophila* S2 cells and larvae, where it regulates lipid metabolism and growth [36]. Notably, among the enhancers that we found to be sensitive to mutation of Deaf1 binding sites, are as follows: CBP2862, located ~ 150 bp upstream of *four wheel drive* (*fwd*), which encodes a Golgi-localized lipid kinase that is expressed in mesoderm, and ChIPCRM2847, located in an intron of the insulin receptor, which is also expressed in mesoderm, and which controls the growth of *Drosophila* larval skeletal muscles [48]. Both of these Deaf1 motif effects are sensitive to the presence or absence of Schlank motifs (Fig. 5 and Additional file 4: Table S4). Altogether, these results suggest a role for Schlank as a transcriptional regulator of fat metabolism and growth in the developing embryo. One prior study reported ZIPIC to act as an insulator protein [33], while another found it to support long-range genomic interactions in *Drosophila* [34]. Our results suggest that the zinc finger protein ZIPIC may also act as a transcriptional repressor and/or modulate the activity of nearby TFs. Such multifunctionality in transcriptional regulation by a DNA-binding zinc finger protein has been observed before for CTCF [49]. Finally, although we previously found the CG12236-PB DNA binding motif to be enriched among mesodermal enhancers [1], to our knowledge, our QeFS results are the first to demonstrate that the CG12236-PB motif regulates enhancer activity and suggest that CG12236-PB may act as a previously unknown transcriptional regulator in *Drosophila* embryonic mesoderm. In all cases, the effects of mutating binding sites of a TF were highly context-dependent, in that effects varied across different wild type enhancers and when different combinations of additional motifs were also mutated.

The large number of mutations that showed no detectable effect on enhancer activity is surprising in light of a recent report of densely encoded regulatory information in a *Drosophila* enhancer [50]. The *shavenbaby E3N* enhancer chosen for that study was selected for its small size and the large amount of patterning information that it integrates, and thus may not be similar to the mesodermal enhancers we mutated in larger fragments. Alternatively, the motifs that we chose to mutate, for relatively understudied factors with ubiquitous expression, might be atypical in their tolerance of mutation. Future experiments could investigate how the observed contributions of these *cis*-regulatory motifs might differ among more specific subpopulations of mesodermal cells.

**Fig. 6:**
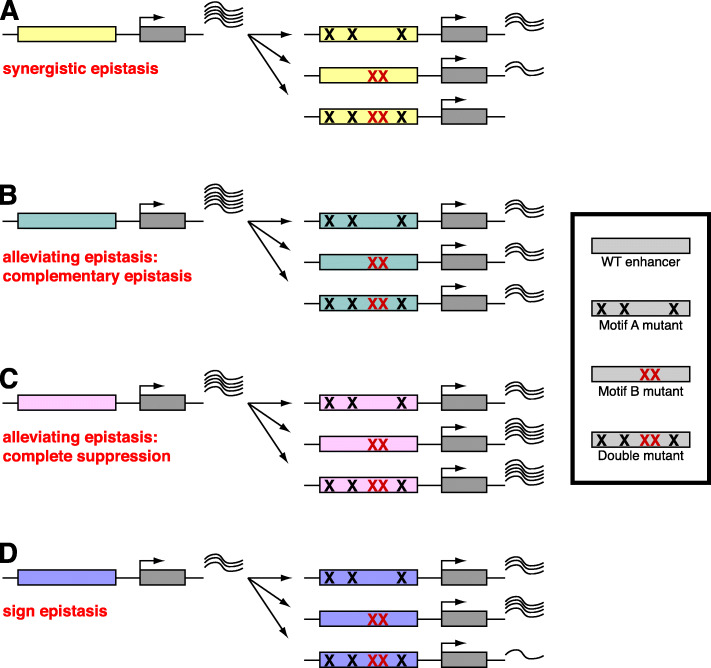
Epistatic interactions identified by QeFS. **A** Synergistic epistasis is observed when mutating two classes of sites produces a greater effect than a multiplicative model predicts from the results of single class mutants, as when Deaf1 and CG12236-PB sites are mutated in ChIPCRM5432. **B**, **C** Alleviating epistasis describes an effect smaller than that predicted by a multiplicative model, and can be subclassified [51]. In this study, we observed **B** complementary epistasis, where the severity of effects of the double mutant equals those of each of the single mutants, as when Deaf1 and CG12236-PB sites are mutated in ChIPCRM2847, and **C** complete suppression, where the effects of mutating one motif suppress the effects of mutating another motif (i.e., severity of effects of the double motif mutant A+B = severity of effects of the single motif mutant B < severity of effects of the single motif mutant A), such as Schlank site mutations do to Deaf1 site mutations in CBP2862. **D** Sign epistasis describes a situation wherein the direction of the effect of mutating a class of sites is reversed in the context of mutations in another class of sites, as we observed when mutating ZIPIC and Deaf1 sites in CG7759_33

Our results across these 12 developmental enhancers active in embryonic mesoderm development indicate that they integrate a combination of positive and negative regulatory inputs from Deaf1, Schlank, ZIPIC, and CG12236-PB. Furthermore, the use of QeFS in combination with synthesis of a specifically designed set of mutant enhancers allowed us to screen for epistatic interactions between *cis*-regulatory motifs in a highly parallel manner in a developing organism (Fig. 6). Although prior studies have systematically analyzed sequence features of enhancers that are important for their activities [21, 25], to our knowledge, our study is the first to assay the effects of combinatorial motif mutations using genomically integrated enhancers in a whole organism. Notably, a prior study found that the chromatin accessibility profiles, as determined by ATAC-Seq, of genomically integrated reporters are the same as those of the endogenous enhancers [52], suggesting that this strategy is likely to measure activity in the appropriate chromatin context. Here, we observed apparent synergy between motifs, finding in one case that Deaf1 and CG12236-PB pairwise mutations appear to potentiate each other’s individual mutations, leading to an additional loss of activity at ChIPCRM5432. Interestingly, we also found that Schlank site mutations within ChIPCRM2847 suppress the effects of Deaf1 and/or CG12236-PB mutations.

Our results support prior studies that have demonstrated synergy between TF binding motifs [19], such as long-distance cooperative interactions between the Dorsal and Twist activators’ motifs in the *rhomboid* enhancer in *Drosophila* embryos [53]. Such synergy between TF motifs was also observed in an imaging-based analysis of *eve* enhancer activity in a subset of muscle and cardiac cells in developing *Drosophila* embryos [54], where combinatorial mutations between dTCF and Twi1 sites or dTCF and ETS3 sites led to a decrease in the number of cells with reporter activity. This is a possible phenomenon underlying some of our observations of synergistic activity, although by QeFS alone we cannot distinguish between altered levels of reporter activity across all cells and altered numbers of cells exhibiting reporter activity. As the GFP reporter transcript we use here expresses a functional protein, complementary imaging studies using our backbone can be performed to test these two different hypotheses.

Our finding of sign epistasis between Deaf1 site mutations and ZIPIC site mutations in the CG7759-33 enhancer, where we found that ZIPIC putatively acts as a repressor, is consistent with a prior study of point mutations in the canonical lambda bacteriophage promoter *P*_*R*_ that affect binding by RNA Polymerase and the TF cI, where thermodynamic modeling of that system indicated that sign epistasis cannot exist in the absence of a repressor [55]. Sign epistasis may be an outcome of combinatorial regulation by multiple TFs in a cell-type-specific or condition-dependent manner, in which the regulatory output of a TF is modulated by its co-regulatory factors. For example, in the scenario of a TF that accelerates a rate-limiting step in the transcription cycle and inhibits another step, a second TF that also activates the first step to the degree that the second step now becomes rate-limiting, may result in the overall output of the first TF being switched from activation to repression [56].

## Conclusions

Epistatic interactions between TF binding sites show extreme context dependence, so that even the direction of epistasis observed between the same motifs can be different in the context of different enhancers (Fig. 3). Uncovering the logic of combinatorial regulation will thus require sensitive, quantitative methods for parallel assay of large numbers of sequences. Analysis of a broader set of motifs and enhancers may reveal the sequence context features that influence when particular combinations of motifs act synergistically. Here, we focused on investigating four TF binding site motifs in a set of 88 wildtype and mutant mesodermal enhancers. The use of degenerate sequence tags and enhancer barcodes enables QeFS to be scaled up in future studies to assay mutational effects in hundreds of enhancers. QeFS could be applied to investigate the effects of SNPs within enhancers on enhancer activity in living organisms. Application of QeFS to other cell types and species should aid in elucidating the *cis*-regulatory codes, including context-dependent epistatic interactions among *cis*-regulatory motifs, underlying cell-type-specific enhancer activity.

## Methods

### Design and synthesis of barcoded enhancer constructs for QeFS

We designed each version (WT, 3 single-motif mutants, 3 motif-pair mutants, triple motif mutant) of each enhancer to be flanked by a unique 8-mer barcode. Briefly, barcodes were chosen to reduce the introduction of potential functional DNA binding sites adjacent to the enhancer sequence and to enable distinguishing of similar enhancer sequences despite potential mutations in the sequence during cloning or errors during lookup table library sequencing. All 8-mer sequences were first filtered against a set of fly TF protein binding microarray (PBM) data [1, 57], and any 8-mer with a PBM E-score > 0.3 from any PBM experiment was eliminated as a candidate barcode. A similar filter was applied based on empirical thresholding against a set of 86 representative position weight matrices (PWMs) [1]. The remaining barcodes and their reverse complements were filtered to achieve a minimum Hamming distance of 3 between any two remaining barcodes. Filtering with these criteria yielded 101 high-stringency, 8-mer barcodes, 100 of which we used in the construction of our experimental and control libraries.

Selected enhancers were scanned with the Deaf1, ZIPIC, Schlank, and CG12236-PB PWMs [1]. Matches with a log-likelihood ratio, calculated relative to the background nucleotide distribution, greater than 6 were considered further. For each motif’s sites, occupancy scores were calculated, with an occupancy of 1 corresponding to the optimal site. Deaf1 and CG12236-PB have compact motifs with little degeneracy, making motif matches unambiguous. Schlank sites with predicted occupancy scores less than 0.5 and ZIPIC sites with predicted occupancy scores less than 0.05 were eliminated from further consideration. Mutations to selected sites were designed manually to maximally disrupt predicted binding affinity according to PWM score while avoiding creation or disruption of overlapping sites for TFs anticipated to be relevant to mesoderm development. In most cases (118 out of 135 motif instances), a single nucleotide change was sufficient to disrupt predicted binding, while the largest change involved mutation of just 3 nucleotide positions. Mutant enhancers differed from wildtype by a minimum of 1 nucleotide and a maximum of 19.

Wildtype and mutant enhancers were synthesized with flanking barcodes and Gateway Clonase recognition sequences with the following structure for cloning into our reporter constructs: Gateway attB1 site, primer binding site (SQ3), enhancer barcode, Bmt I restriction enzyme site, enhancer element (wildtype or mutant), reverse complement of the same barcode, primer binding site (rc(SQ5)), and the reverse complement of the Gateway attB2 site. All but one (the Deaf1 and ZIPIC double mutant allele for the enhancer ChIPCRM2000) of the resulting 96 sequences were successfully synthesized and cloned into the pUC19 vector (Gen9). The Act57B and β3Tub60D control enhancers described in the text were prepared by PCR from OreR genomic DNA followed by site-directed mutagenesis, with cloning sites and barcodes added as above. Sequences of all tested constructs are provided in Additional file 3: Table S3.

### Construction of enhancer-reporter library

As the original enhancer-FACS-seq approach was based upon a DNA-seq readout of abundance of the enhancer element, but QeFS is based upon paired RNA- and DNA-seq readout of reporter transcripts and templates through targeted sequencing of degenerate tags in the 3′ UTR of the reporter element, the original pEFS reporter backbone [1] needed to be modified in several ways. Ligation-free mutagenesis was used to introduce two sets of primer binding sites into the 3′ UTR of the reporter gene template. These primers facilitate later construction of the lookup table sequencing library and the RNA- and DNA-seq libraries. Additionally, conventional restriction digest cloning was used to introduce a library of degenerate 20-mer sequences into the 3′ UTR of the reporter template in a parallel fashion, thus creating a library of reporter backbones, each with a unique 20-mer tag.

Each enhancer construct was cloned independently into the pQeFS reporter backbone and individually validated by Sanger sequencing before being pooled at equimolar concentrations, creating a final enhancer-reporter library of ~ 320 unique enhancer-reporter constructs suitable for injections. The enhancer-reporter library was constructed such that there are multiple reporter tags per enhancer, but with each tag linked to a unique enhancer. We determined the uniformity of enhancer representation in this pool and generated a lookup table that associates enhancer barcodes with reporter tags, by a custom high-throughput sequencing approach.

### Generation and sequencing of lookup table

To generate the lookup table, a portion of the library pool was first digested with BmtI, which cleaves (1) in the 3’ UTR of the reporter transcript, directly adjacent to the degenerate 20-mer tag, and (2) directly adjacent to the enhancer barcode that is most distal to the reporter promoter, and then re-circularized by intramolecular ligation. The re-circularized product juxtaposes the enhancer barcode in close proximity to the degenerate 20-mer. The ligation product was purified by a Zymo DNA Clean and Concentrator column. PCR was then used to amplify the product of interest and label the product with adapters (Illumina P5 and P7, a custom sequencing primer, and the Illumina index read 1 primer) necessary for Illumina sequencing. Following amplification, the library was size-selected with SPRI beads at a ratio of 0.8x beads:library products.

A custom sequencing primer (“LUT_sequencing”) was designed and used to sequence the lookup table library on an Illumina MiSeq instrument with 70 cycles of read 1 sequencing and a 6 cycle index 1 read. A total of 35,247,345 raw reads were sequenced, and 33,876,153 Passed Filter reads were identified, a rate of 96%. A total of 33,322,759 reads had the expected Illumina index.

Enhancer barcode-tag pairs obtained by lookup table sequencing were compared to expected barcode-tag pairs obtained by Sanger sequencing during library cloning to define true positive pairs. In seven cases, additional tags were defined as positive following empiric thresholding of barcode-tag sequencing read counts for individual enhancer barcodes. This final list of barcode-tag pairs was used to build a pseudo-genome for mapping QeFS sequencing reads.

### FACS and preparation of QeFS sequencing libraries

The enhancer-reporter library was injected into flies (Rainbow Transgenic Flies) from the strain *w- nos-ΦC31; attP40*. Progeny of injected flies, each of which contained a single genomically integrated enhancer-reporter, were crossed to CD2 driver lines expressing rat CD2 under the control of a cell type-specific driver (the *twi* promoter for whole mesoderm, or the *Mef2-I-E*_*D5*_ enhancer and the *Hsp70* TATA box for fusion-competent myoblasts (referred to as *mef2*)). Embryos from these crosses were harvested, aged to the desired stage, and processed to prepare single-cell suspensions suitable for FACS. We performed 2 weeks of collections of both CD2+ and CD2- populations, with three collections per week, for *twi*:CD2 crosses and for *mef2*:CD2 crosses (Additional file 2: Table S2).

We used a custom high-throughput sequencing library preparation method to generate two libraries (paired RNA- and DNA-seq) for both CD2+ and CD2- collections for each day. For the QeFS collections, each week’s libraries were indexed, pooled, and sequenced on an Illumina NextSeq instrument, yielding ~ 5 million to ~ 25 million reads per library.

### Processing of sequencing reads

Processing of sequencing data began with recovery of the UMI sequence from each Illumina sequencing read. We wrote a custom Python script to process individual fastq file entries, parsing the first 8 bases off the beginning of each read and appending this sequence to the read name. The utility cutadapt was then used to trim off the ~ 20-nt constant region from the new 5′ end of each read (example command: cutadapt -g CGCGGGATGCTAGCACGCGG -e 0.3 -o CTW30OutFive.fq.gz --format fastq CTW30.out.gz). Cutadapt was then used to trim the constant 3′ end of each read off as well, leaving the ~ 20-nt sequence that should be unique to each reporter element (example command: cutadapt -a ACATATAGGACCAG -e 0.3 -m 10 -o CTW30OutFiveThree.fq CTW30OutFive.fq.gz). In preparation for mapping the degenerate reporter tags back to their corresponding enhancer, the bowtie-build command was used to build a custom “genome” index of the barcode-tag pairs obtained from the lookup table, where each pseudo-chromosome was a particular enhancer, and each pseudo-chromosome sequence was the degenerate tag sequence. Thus, the pre-processed FASTQ file containing the trimmed degenerate tags could be aligned to this custom index with bowtie allowing 2 mismatches along the ~ 20-nt sequence. The output was then parsed using a second custom Python script to generate a count of per-tag UMI reads, which are the final read-out of enhancer activity.

### Analysis of QeFS sequencing data

The sequencing library preparation incorporated a UMI label for each cDNA or DNA template that enabled quantification of starting RNA or DNA abundance, respectively, to minimize effects of potential amplification bias. Sequencing libraries were processed to obtain a final per-reporter UMI count for each tag in both the DNA- and RNA-seq libraries. Each UMI count was scaled by the total number of processed tags in a given sequencing library, thereby scaling for overall read depth of the library. To scale each RNA count (corresponding to enhancer activity) to the abundance of a given enhancer in the underlying fly population, per-tag UMI totals for RNA-seq libraries were scaled by corresponding per-tag UMI totals for paired DNA-seq libraries from the same collection, scaling the reporter signal to the underlying abundance of a given reporter in the fly population (Eq1).

1 $$ {Expression}_{tag_A}=\frac{\frac{UMI_{RNA,{tag}_A}}{\sum {UMI}_{RNA,{tag}_n}}}{\frac{UMI_{DNA,{tag}_A}}{\sum {UMI}_{DNA,{tag}_n}}} $$

Tags that did not meet a minimum empirical threshold of 65 DNA read counts per tag per day were removed; this type of thresholding is consistent with other quality control thresholds used in a prior MPRA study [24]. In addition, some enhancers were represented by only a small number of tags, either per day or across all three days of collections for a given week. A minimum of at least 4 tags per enhancer per week of aggregated data was required for inclusion of that enhancer construct in the final dataset. Overall, UMI read counts and DNA- or RNA-seq read counts correlated well (e.g., Spearman’s *ρ* = 0.997 and Pearson’s *r* = 0.997 for DNA and Spearman’s *ρ* = 0.999 and Pearson’s *r* = 0.951 for RNA), indicating that there was minimal amplification bias during library preparation (Additional file 1: Figure S12).

### Statistical analysis

Data were aggregated by treating all observations for a given enhancer in a given week as independent biological replicates. Thus, if an enhancer was represented by three different tags on three consecutive days, there would be nine total observations for this enhancer. Differences in expression within libraries due to site mutations were determined through nonparametric one-way analysis of variance testing using the Kruskal-Wallis (KW) test. This test was applied across each series of enhancer mutant constructs (WT and individual and combinatorial mutants) following aggregation within each week to determine if there were significant differences in expression across the series. Post-hoc pairwise testing within an allelic series was performed using the Conover-Iman test [58], which is valid if and only if the null hypothesis of no difference between groups is rejected following the KW test, and preserves the ranks used in the KW omnibus test. Benjamini-Hochberg multiple comparison adjustment was applied to calculated *P*-values, and an alpha value of 0.1 was used for calling significantly different expression levels. All results from this test are provided in Additional file 4: Table S4. All *P*-values reported for QeFS pairwise comparisons between constructs were obtained by this method.

For display purposes, mean enhancer activity was calculated for each enhancer over all replicate measurements, and normalized to “Fold Change” by dividing by the activity of the corresponding wildtype enhancer. Standard error of the mean was also calculated, and a propagated standard error of the mean was calculated for the Fold Change measurement as described previously [24].

To test for epistasis, here understood as statistically significant deviation from the prediction of a multiplicative model [44] for combining the effects of separately mutating individual classes of sites, all replicate expression measurements for WT, motif A mutant, motif B mutant, and motifs A+B mutant constructs were log-transformed and modeled with the lm function in R. All *P*-values reported for such effects represent the *P*-values associated with the interaction term.

### Additional method details

For additional methods, please see Additional file 2: Supplemental Methods [59].

## Supplementary information

**Additional file 1: **Supplementary Figures. **Figures S1–S32**.

**Additional file 2: **Supplemental Methods and **Tables S1** and **S2**.

**Additional file 3: Table S3.** Sequences of tested elements.

**Additional file 4: Table S4.** QeFS processed data.

**Additional file 5:** Peer review history.

## Data Availability

Sequences of the tested enhancers described in this study are available in Additional file 3: Table S3. The normalized expression measurements and results of all pairwise statistical tests appear in Additional file 4: Table S4. The raw sequencing data from this study have been submitted to the NCBI Gene Expression Omnibus under accession number GSE149908 [60].
