# Supplementary material for: Quantitative-enhancer-FACS-seq (QeFS) reveals epistatic interactions among motifs within transcriptional enhancers in developing Drosophila tissue
Source: Genome Biol. 2021 Dec 20;22:348. doi: 10.1186/s13059-021-02574-x (PMC8686523; doi:10.1186/s13059-021-02574-x)
Supplement: Supplementary file 1 — Additional file 1: Supplementary Figures. Figures S1–S32. [file 13059_2021_2574_MOESM1_ESM.pdf]

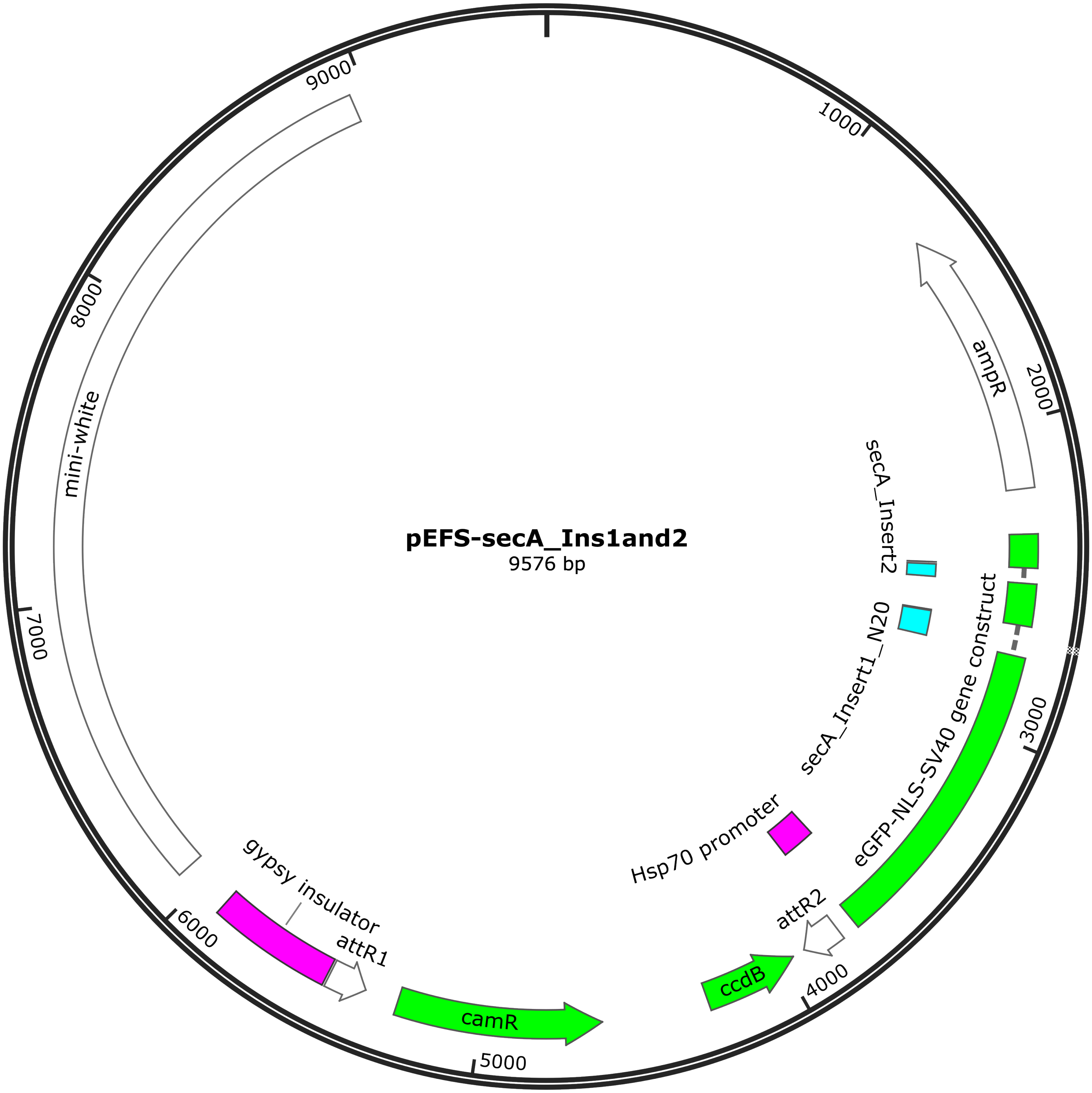

Fig. S1. Plasmid map of the final pQeFS construct. pQeFS was built using the original pEFS backbone, with the addition of inserts in the 3' UTR of the eGFP-NLS-SV40 construct. The inserts are indicated in teal. The secA_Insert1_N20 insert contained the degenerate N20 tag region, and the secA_Insert2 contained the reverse transcription primer binding site and an additional primer binding site. The GFP reporter is transcribed counter-clockwise from the Hsp70 minimal promoter. Enhancers were cloned using the two attR sites, swapping out the ccdB positive selection marker and the chloramphenicol resistance (camR) cassette. The backbone also contains the Drosophila mini-white gene, and an ampicillin resistance (ampR) gene.

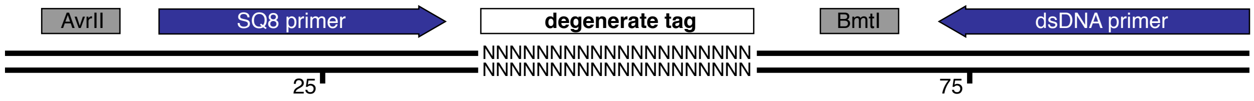

Fig. S2. Schematic of dsN20 oligo cloned into the 3' UTR of the reporter transcript. This element was cloned into the double-digested reporter element using the Avr II and Bmt I cut sites (grey). The dsDNA_primer was used to double-strand the single-stranded oligo (IDT), but was cleaved off by digestion with BmtI and AvrII prior to cloning into the corresponding site in pQeFS.

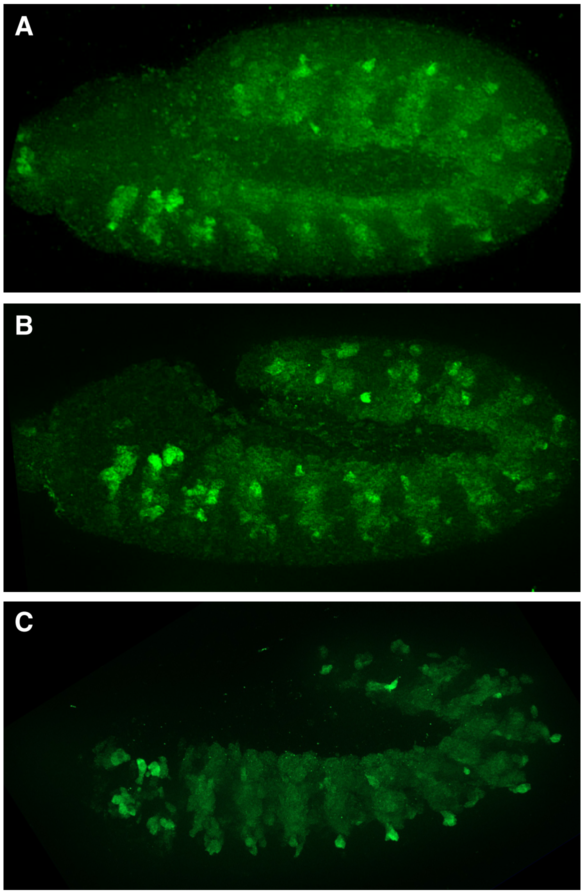

Fig. S3: Validating that modifications to the pQeFS vector do not significantly influence reporter expression. Candidate CRMs in either the pEFS or pQeFS backbone drove similar levels and locations of reporter gene activity. Embryos were stained for reporter GFP expression (green) driven by the CG7759_33 CRM. Maximum intensity projections show that CG7759_33 drives strong mesoderm-specific expression in pEFS (**A**), which is not detectably altered by the inclusion of degenerate tag and primer sequences in the reporter gene 3' UTR in pQEFS (**B**), or by the inclusion of a barcode sequence at the ends of the CRM (**C**). (Panel **A** adapted from Gisselbrecht *et al.*, *Nature Methods* (2013).)

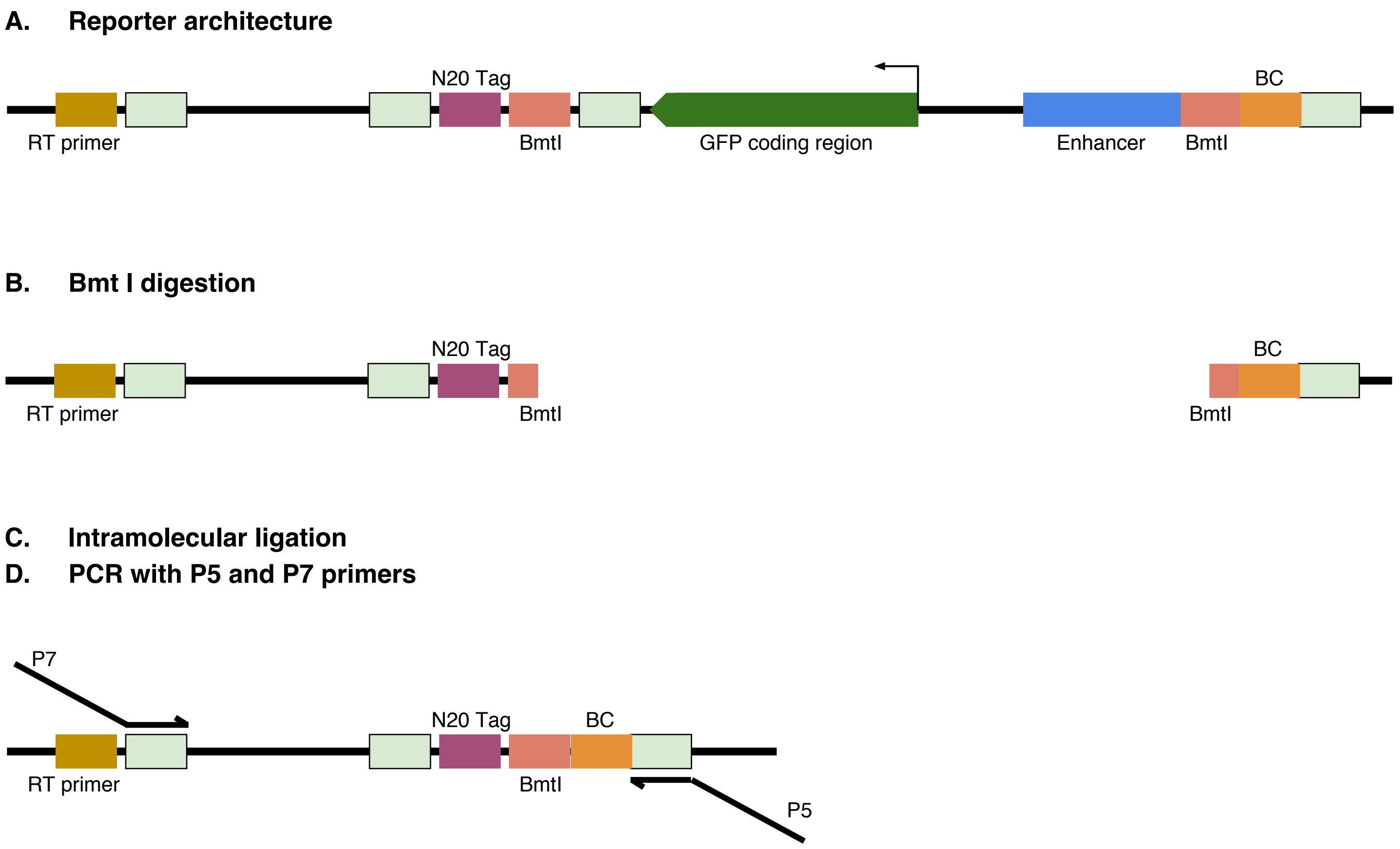

Fig. S4. Overview of lookup table library preparation. (**A**) Architecture of the relevant region of the enhancer-reporter construct. From left to right, in the 3' UTR of the reporter template was a reverse transcription primer binding site (mustard), two primer binding sites (light green), the degenerate 20-mer Tag (purple), the BmtI digestion site (pink), an additional primer binding site (light green), and then the GFP coding region (green). The two regions that were introduced using ligation-free cloning were the RT primer and adjacent primer, and the Tag, Bmt I site, and adjacent primers. Upstream of the coding region was the enhancer (blue), a Bmt I digestion site (pink), the enhancer barcode (orange), and a primer binding site (light green). (**B**) Bmt I digestion excises the GFP coding region and the enhancer sequence. (**C**) Intramolecular ligation results in circularization of the digested plasmid (juxtaposing N20 Tag) and the enhancer barcode ("BC"), and (**D**) PCR with Illumina P5 and P7 cluster generation primers resulted in the construction of a product suitable for Illumina high-throughput sequencing.

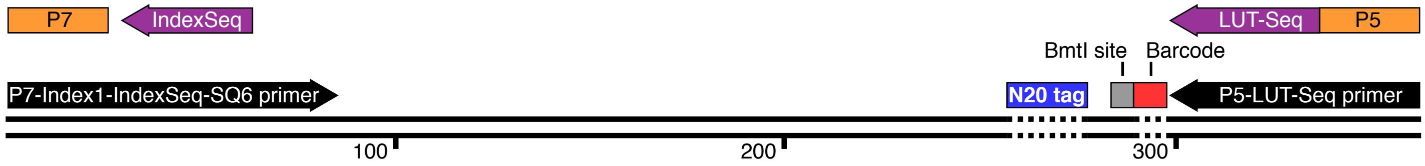

Fig. S5. Schematic of look-up table library product following recircularization and PCR addition of primer sequences. The region of interest was the juxtaposed dsN20 element (blue) and the enhancer barcode (red). These were brought into proximity through the intramolecular ligation at the Bmt I site. PCR was used to add on the P7_Index1_IndexSeq_SQ6 sequence and the P5_SQ3 sequence using homology between these primers and the SQ6 and SQ3 regions present in the reporter element, respectively. The Illumina cluster generation primers P5 and P7 are indicated in orange, and the sequencing primers for read 1 (the custom sequencing primer LUT_seq) and the index read (the standard Illumina index sequencing primer IndexSeq) are indicated in purple. The overall amplicon is 361 bp and was designed to facilitate efficient cluster generation on the Illumina chip.

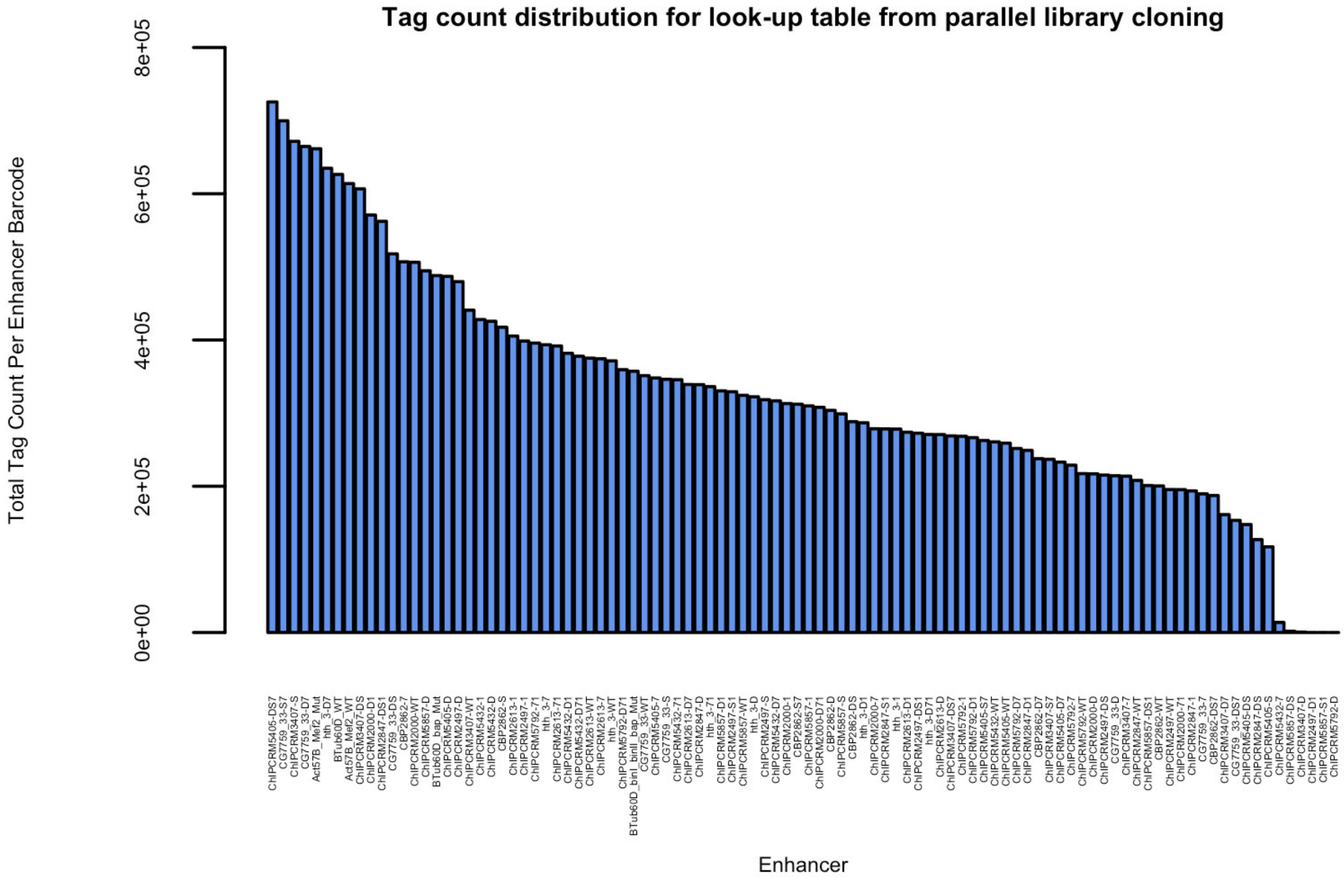

Fig. S6. Distribution of tag counts for the lookup table. For each enhancer barcode, the total tag counts per barcode were summed and plotted.

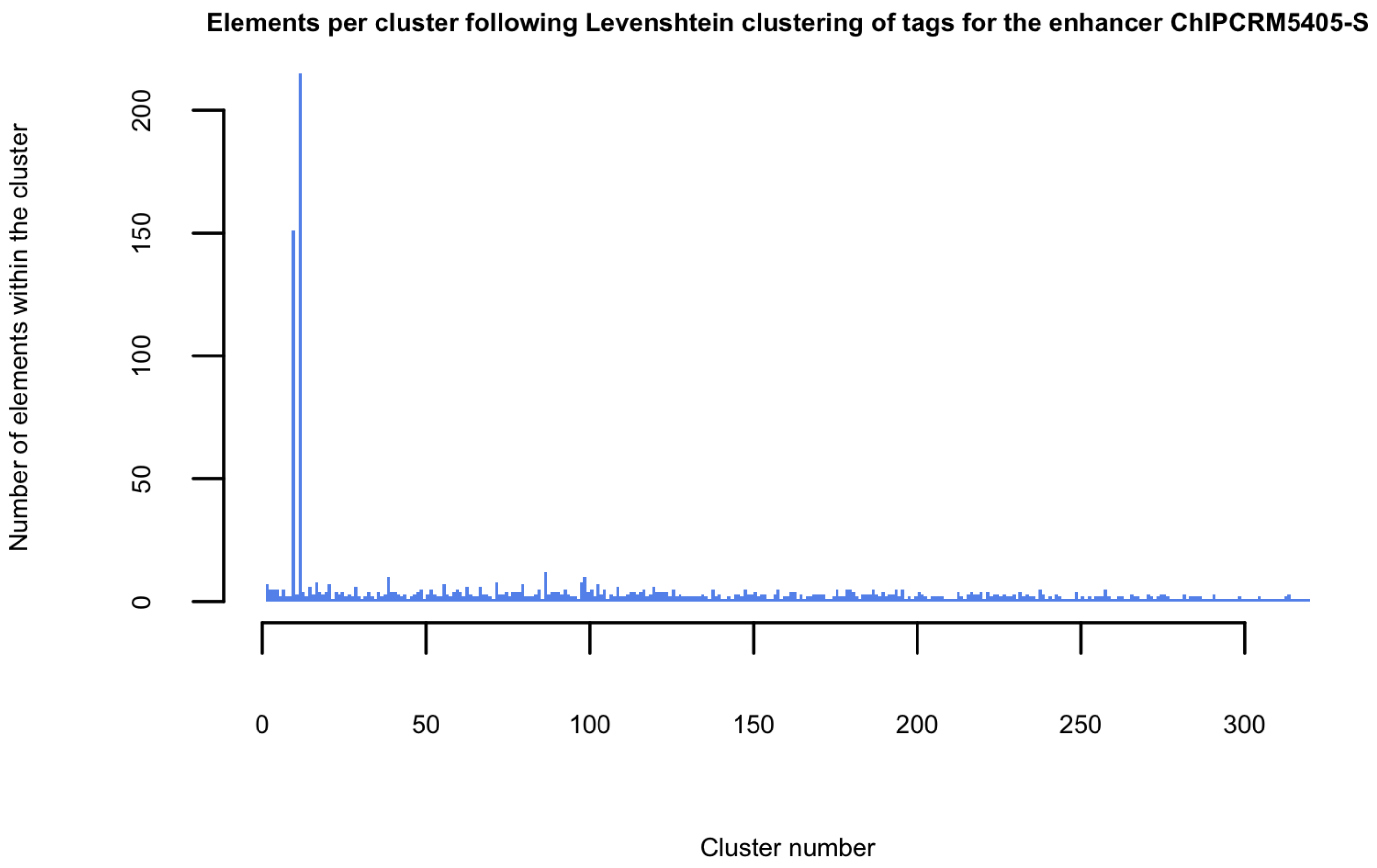

Fig. S7. Number of elements per cluster following Levenshtein clustering and tree cutting to 320 groups for the enhancer ChIPCRM5405-S. The two predominant clusters corresponded to variants of the expected tag for the ChIPCRM5405-S barcode. The low abundance clusters arise from spurious intermolecular ligations between different enhancer-reporter constructs.

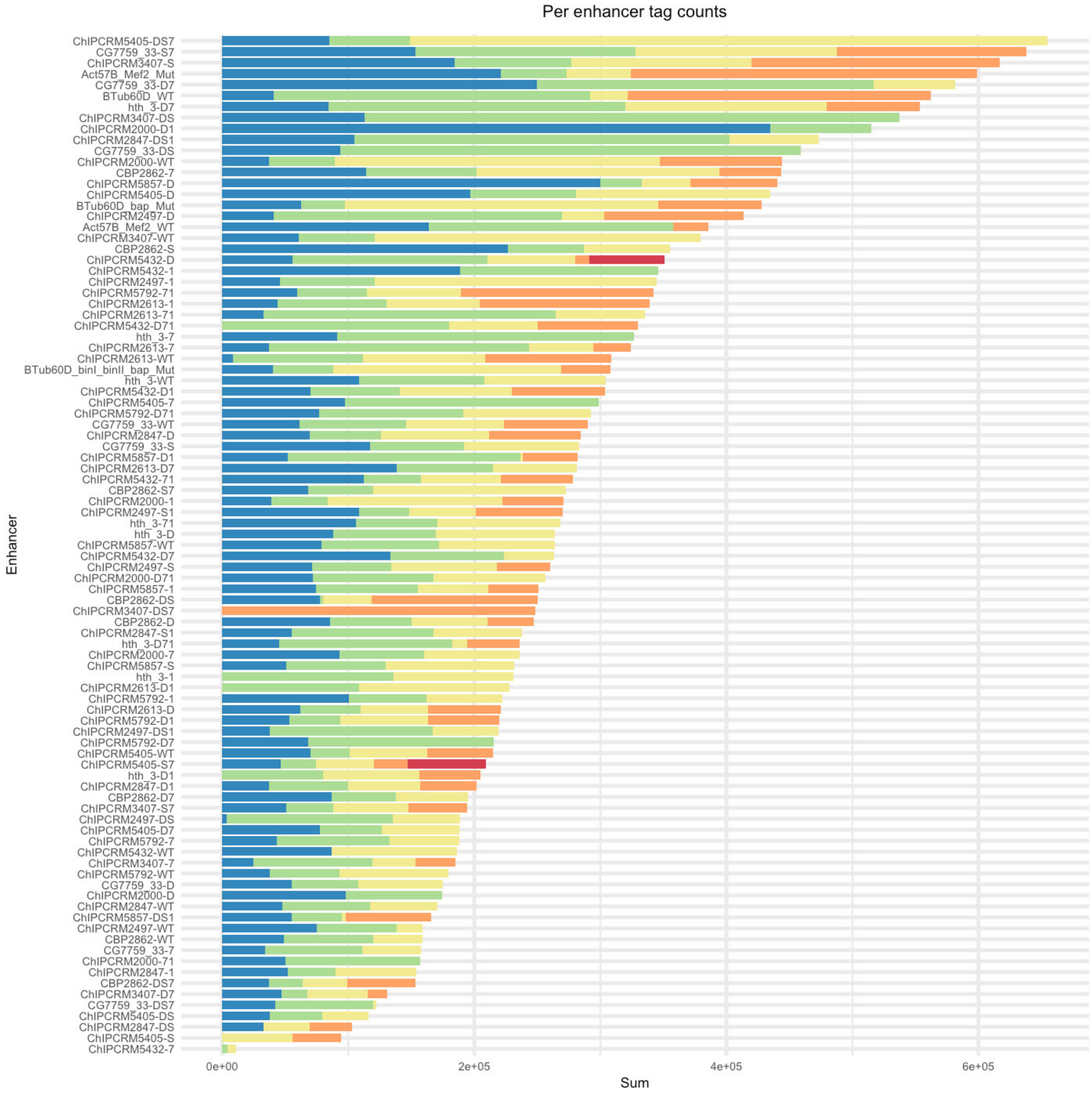

Fig. S8. Tag count totals for each enhancer. For each expected enhancer barcode, the total tag counts for its expected linked tags are displayed, summing to the total tag counts for the enhancer across all tags. Data are displayed as a stacked bar plot, thus each color of each bar corresponds to the abundance of a given tag for a given enhancer, summing to the total abundance of the individual enhancer in the library.

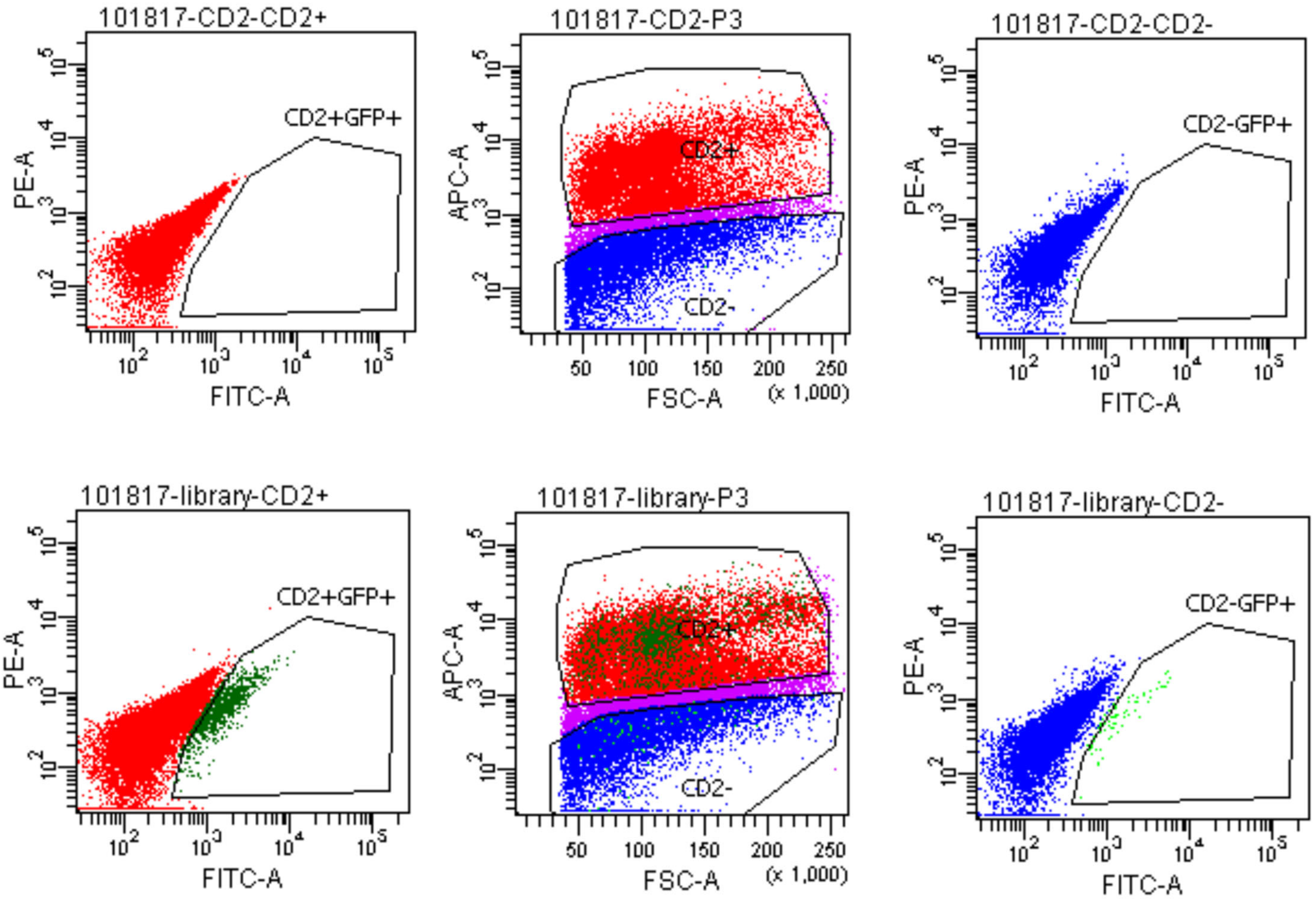

Tube: library

Population	#Events	%Parent	%Total
All Events	100,000	####	100.0
P1	35,019	35.0	35.0
P2	33,028	94.3	33.0
P3	29,597	89.6	29.6
CD2+	17,041	57.6	17.0
CD2+GFP+	1,146	6.7	1.1
CD2-	8,561	28.9	8.6
CD2-GFP+	75	0.9	0.1

Fig. S9. Representative FACS plots for *twi*:CD2 sorting. Plots in the top row are for the *twi*:CD2 parental population. The center plot is the population of single live cells (following gating on FSC, SSC, and DAPI) and displays gates for the CD2+ and CD2- populations. The left plot is the CD2+ population, with a gate indicated for GFP+ cells. The right plot is the CD2- population with a gate indicated for GFP+ cells. The plots in the middle row are for the QeFS experimental population, with the same gates as outlined for the *twi*:CD2 sort. Note that the GFP gates were only used for sorting of the eFS-like population, and during QeFS simply provided quantitation of the fraction of GFP positive cells present in the population. The table in the bottom row provides a breakdown of the population of cells following an analytical sort of 100,000 cells from the QeFS experimental population. Approximately 1.1% of cells were CD2+ and GFP+ in the experimental population vs. 0.001% in the *twi*:CD2 parental line.

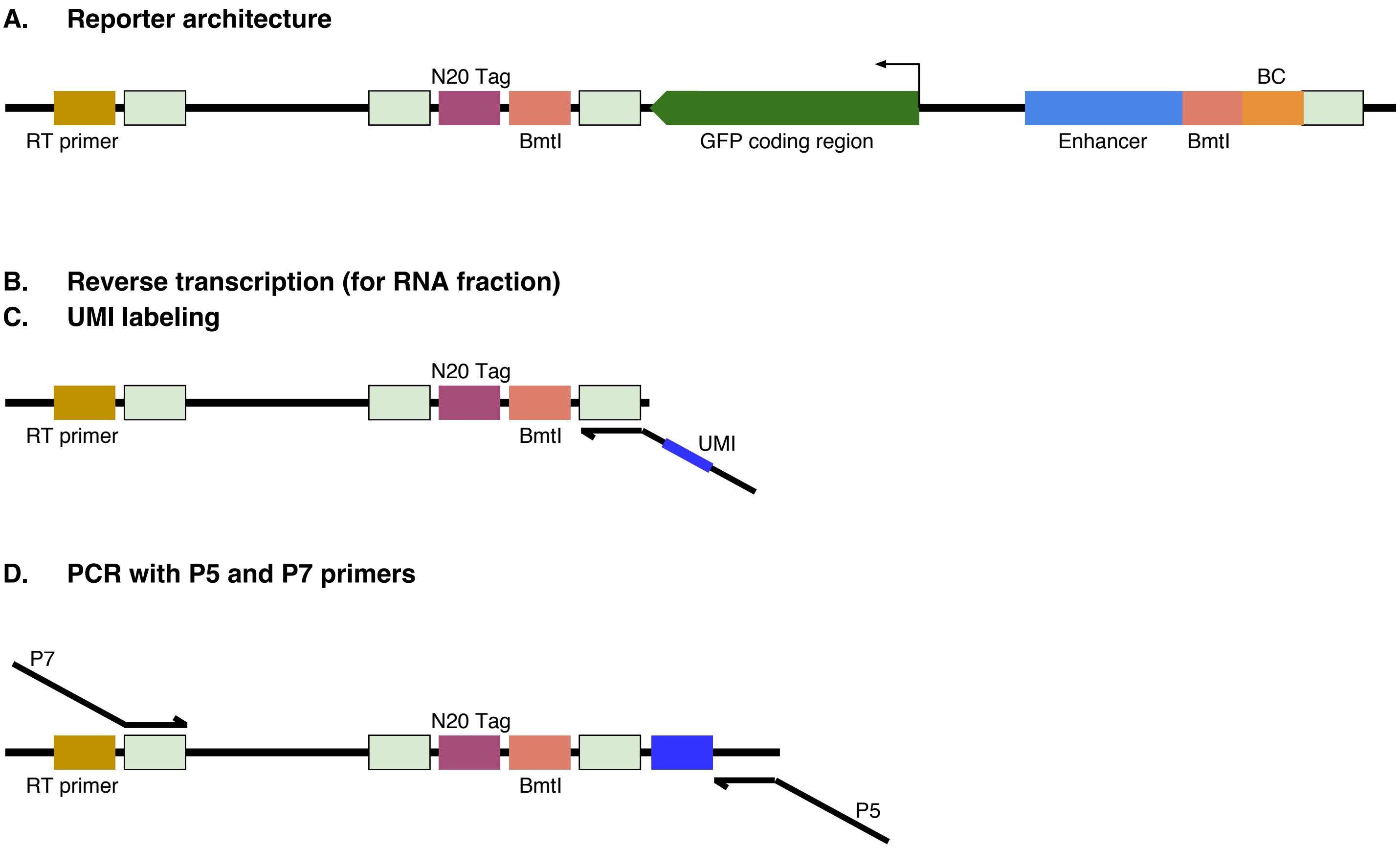

Fig. S10. Overview of QeFS library preparation approach. (**A**) Schematic of the reporter architecture, with particular emphasis on the 3' UTR of the reporter template. From left to right, in the 3' UTR of the reporter template was a reverse transcription primer binding site (mustard), two primer binding sites (light green), the degenerate 20-mer Tag (purple), the Bmt I digestion site (pink), an additional primer binding site (light green), and then the GFP coding region (green). (**B**) RNA transcripts produced from this template were reverse transcribed using a primer that binds to the RT primer site (mustard). (**C**) DNA from genomically integrated transcripts or cDNA from the RT reaction were UMI-labeled in a primer extension reaction and then purified. (**D**) PCR was then performed with a P7- containing primer and a P5-containing universal primer that introduced elements necessary for library indexing, cluster generation, and sequencing.

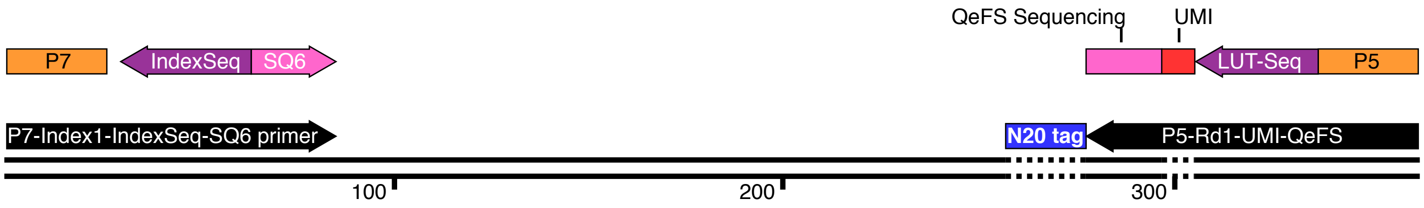

Fig. S11. Schematic of the QeFS RNA- or DNA-seq library amplicon. This schematic displays the generic structure of the final RNA- or DNA-seq library amplicon. The dsN20 region is indicated in blue and was the region of interest from the 3’ UTR of the reporter transcript or template. Additional sequencing elements were added by annealing the black P5- (P5-Rd1-UMI-QeFS) or P7- (P7-Index1-IndexSeq-SQ6) containing primers to sequences embedded in the 3’ UTR and indicated in pink, either the “QeFS_Sequencing” or “SQ6” element, respectively. The P7- containing primer added on the P7 cluster generation sequence, an Illumina TruSeq 6-base index sequence, and a standard Illumina index sequencing primer. The P5-containing primer added on the P5 cluster generation sequence, a standard Illumina read 1 sequencing primer, and a degenerate 8-mer UMI tag. Thus, the first 8 bases that were sequenced originate from the UMI region. The final amplicon was ~363 bp, with some variability due to variability in the degenerate 20-mer region.

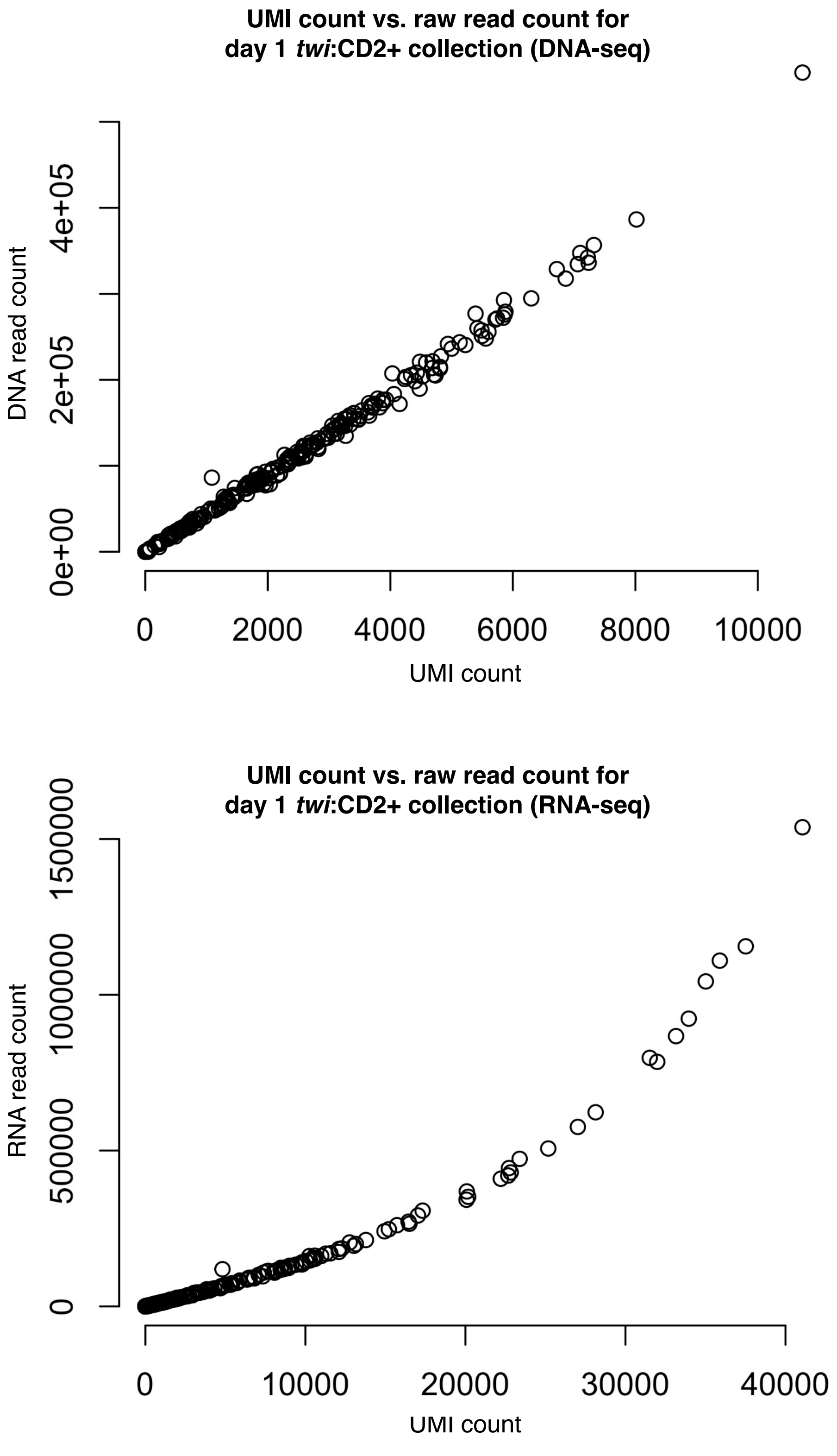

Fig. S12. Concordance between UMI reads and DNA- or RNA-seq reads for the first day of collections of *twi*:CD2+ libraries. Raw UMI counts and raw DNA (upper panel) or RNA (lower panel) read counts are plotted for corresponding DNA- or RNA-seq libraries. DNA UMI and read counts correlate with Spearman's ρ = 0.997 and Pearson's r = 0.997 and RNA UMI and read counts correlate with Spearman's ρ = 0.999 and Pearson's r = 0.951.

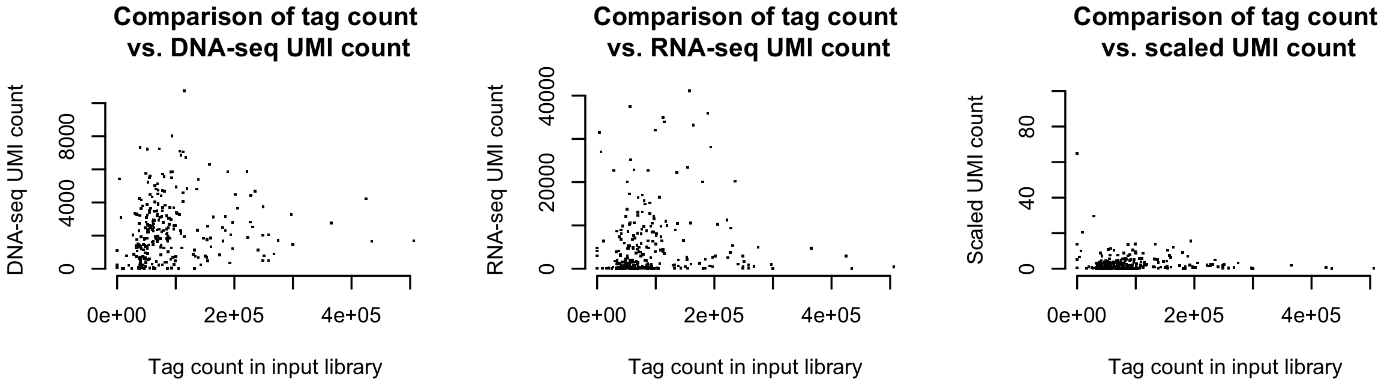

Fig. S13. Correlation of tag counts in input library and UMI counts for DNA and RNA libraries or scaled UMI values. (Left panel) Plot of input tag abundance vs. UMI count in the DNA-seq library (Pearson's r = 0.09). (Middle panel) Plot of input tag abundance vs. UMI count in the RNA-seq library (Pearson's r = 0.06). (Right panel) Plot of input tag abundance vs. scaled UMI expression measurement (Pearson's r = -0.07). Note that the y-axis of the scaled expression measurement plot was adjusted to remove an outlier filtered out in a later analysis.

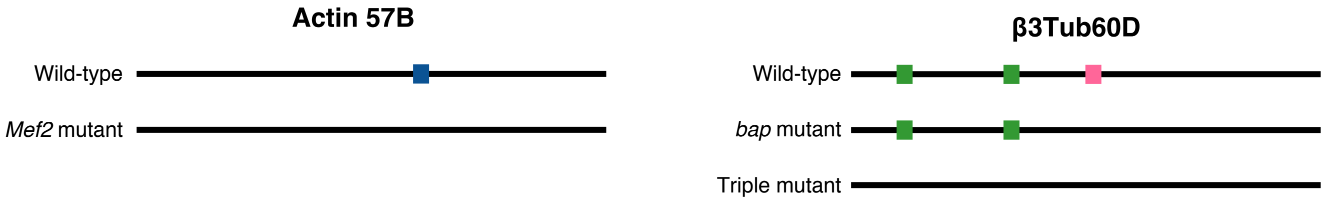

Fig. S14. Schematic of control enhancer sequences. The left panel shows the *Actin 57B* enhancer and the relative position of the Mef2 binding site (blue) that was mutated using PCR mutagenesis. The right panel shows the *β3Tub60D* enhancer with the two mutated bin sites (green) and the mutated bap site (pink).

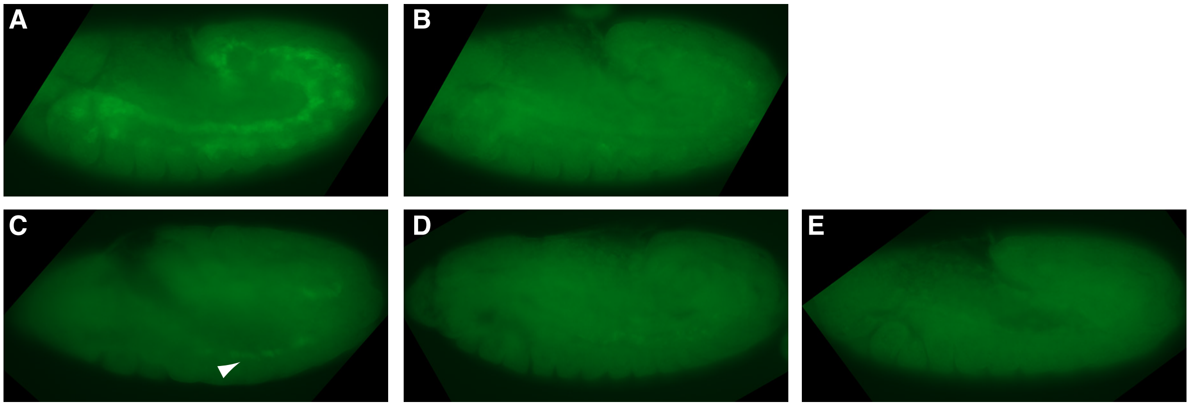

Fig. S15. Imaging of control enhancers in stage 12 embryos. (**A**) Actin 57B WT and (**B**) Mef2 mutant with nearly complete loss of broad mesodermal activity. (**C**) β3Tub60D WT with visceral mesoderm-specific expression (arrowhead). (**D**) Activity was decreased following bap site mutation. (**E**) Activity is lost in the triple mutant.

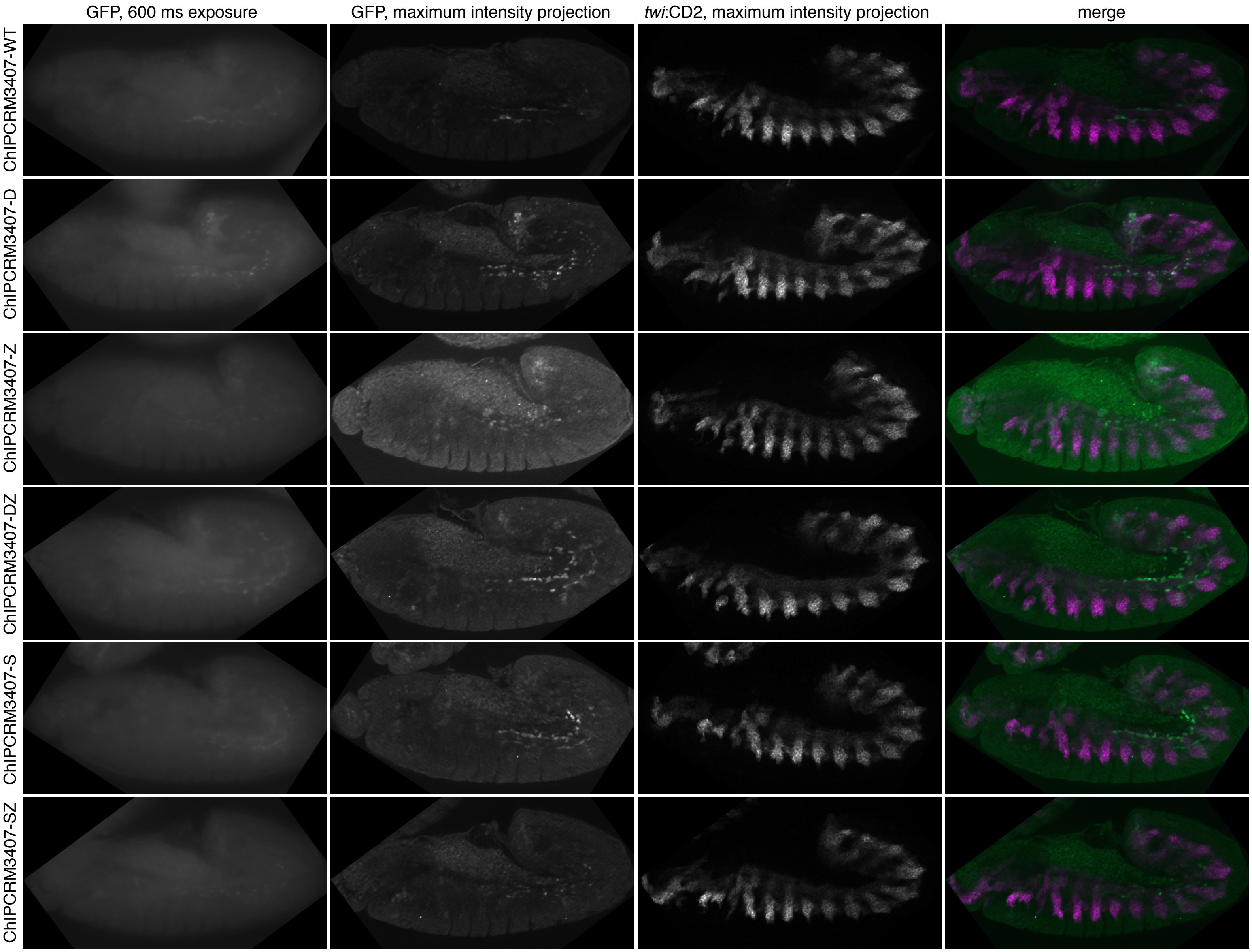

Fig. S16. Imaging of representative embryos from the ChIPCRM3407 series. The left column shows representative age-matched embryos, fixed, stained, and imaged in parallel, with identical exposure and with no adjustment to brightness or contrast, so that intensity can be compared. The remaining columns show reporter GFP expression, *twi*:CD2 pattern, and merge of maximum intensity projections from a Z-series of structured illumination optical sections from the identical embryo, to permit the careful inspection of GFP expression pattern.

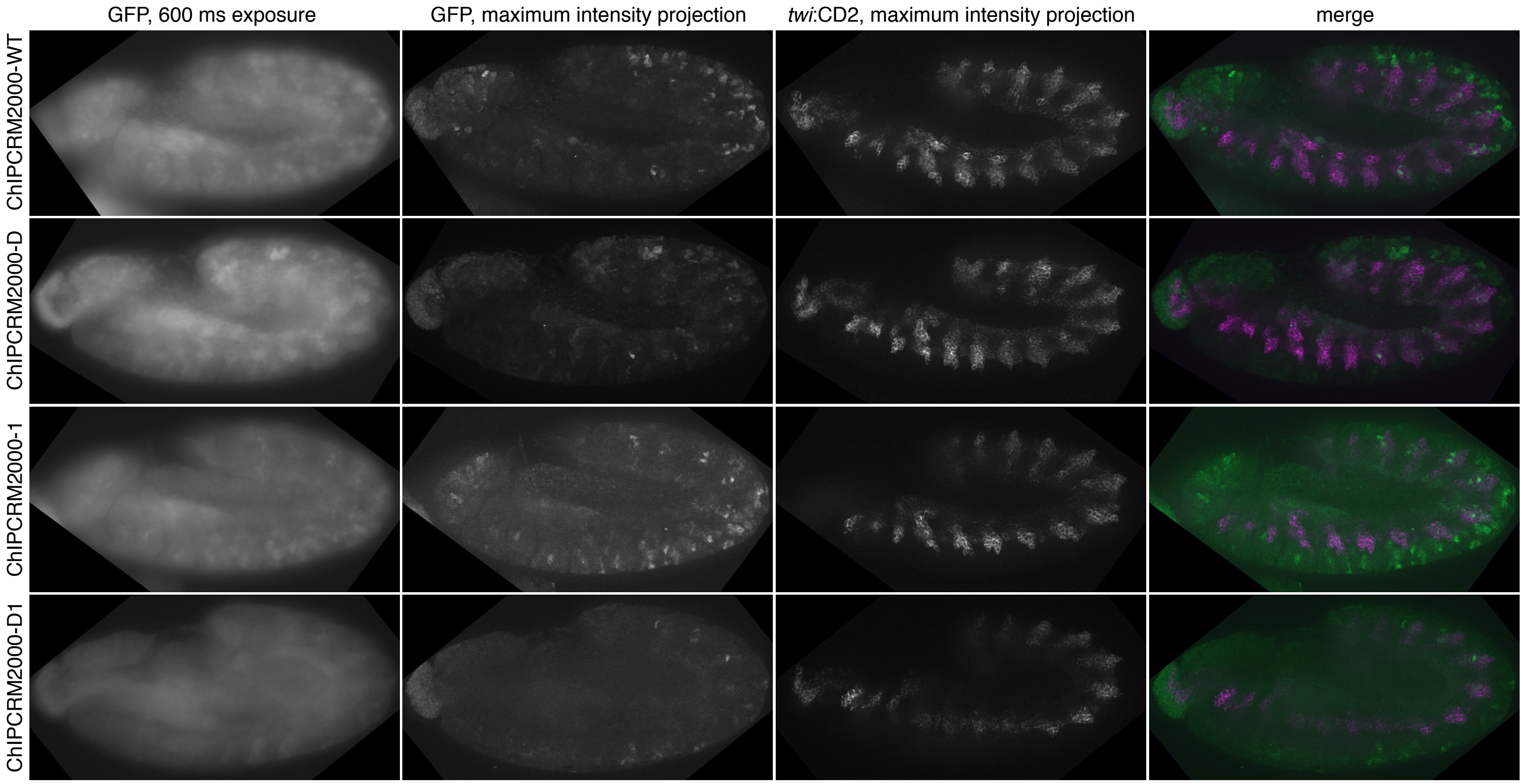

Fig. S17. Imaging of representative embryos from the ChIPCRM2000 series. The left column shows representative age-matched embryos, fixed, stained, and imaged in parallel, with identical exposure and with no adjustment to brightness or contrast, so that intensity can be compared. The remaining columns show reporter GFP expression, *twi*:CD2 pattern, and merge of maximum intensity projections from a Z-series of structured illumination optical sections from the identical embryo, to permit the careful inspection of GFP expression pattern.

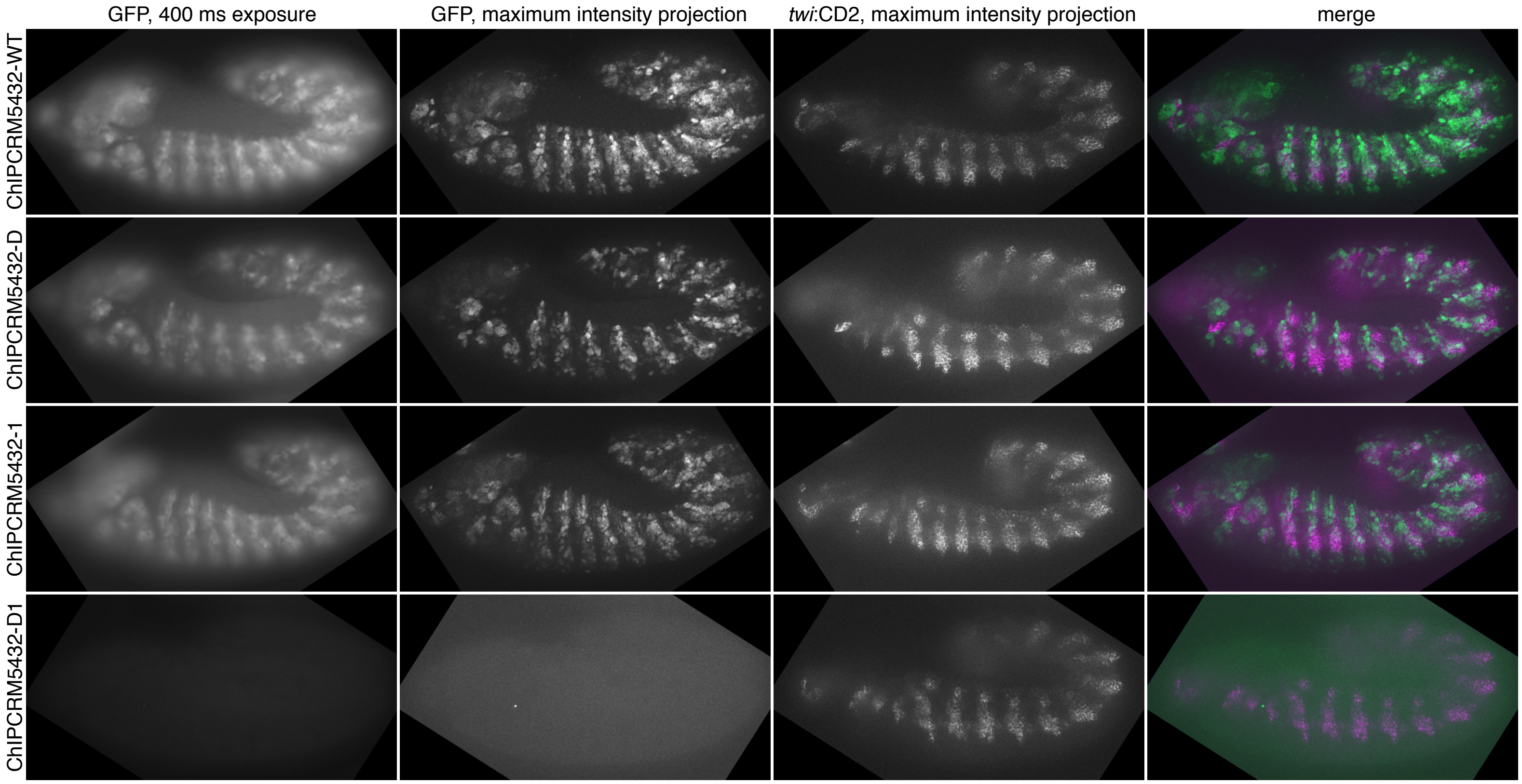

Fig. S18. Imaging of representative embryos from the ChIPCRM5432 series. The left column shows representative age-matched embryos, fixed, stained, and imaged in parallel, with identical exposure and with no adjustment to brightness or contrast, so that intensity can be compared. The remaining columns show reporter GFP expression, *twi*:CD2 pattern, and merge of maximum intensity projections from a Z-series of structured illumination optical sections from the identical embryo, to permit the careful inspection of GFP expression pattern.

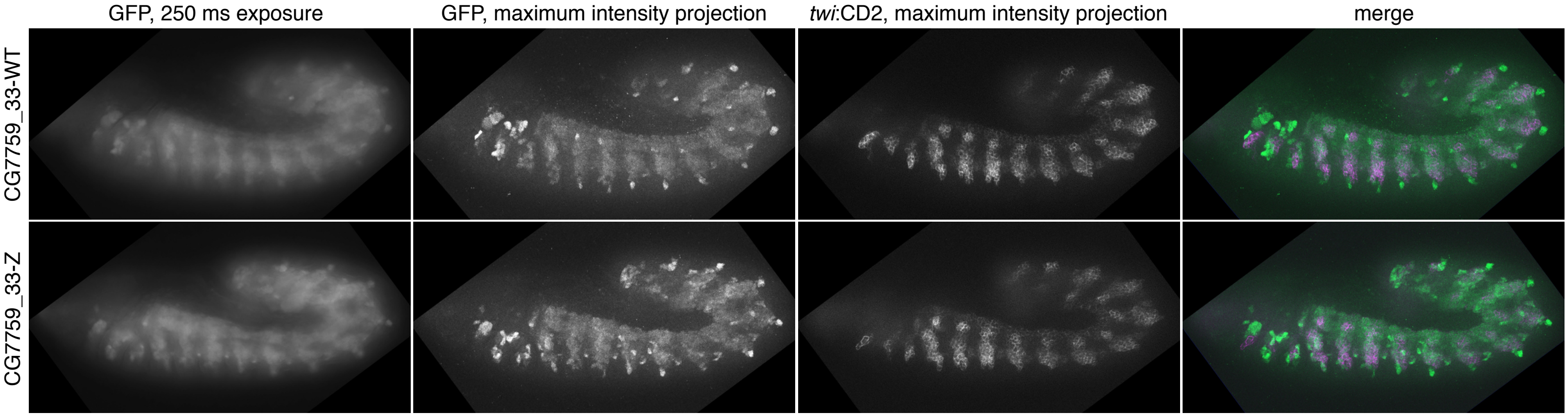

Fig. S19. Imaging of representative embryos from the CG7759_33 series. The left column shows representative age-matched embryos, fixed, stained, and imaged in parallel, with identical exposure and with no adjustment to brightness or contrast, so that intensity can be compared. The remaining columns show reporter GFP expression, *twi*:CD2 pattern, and merge of maximum intensity projections from a Z-series of structured illumination optical sections from the identical embryo, to permit the careful inspection of GFP expression pattern.

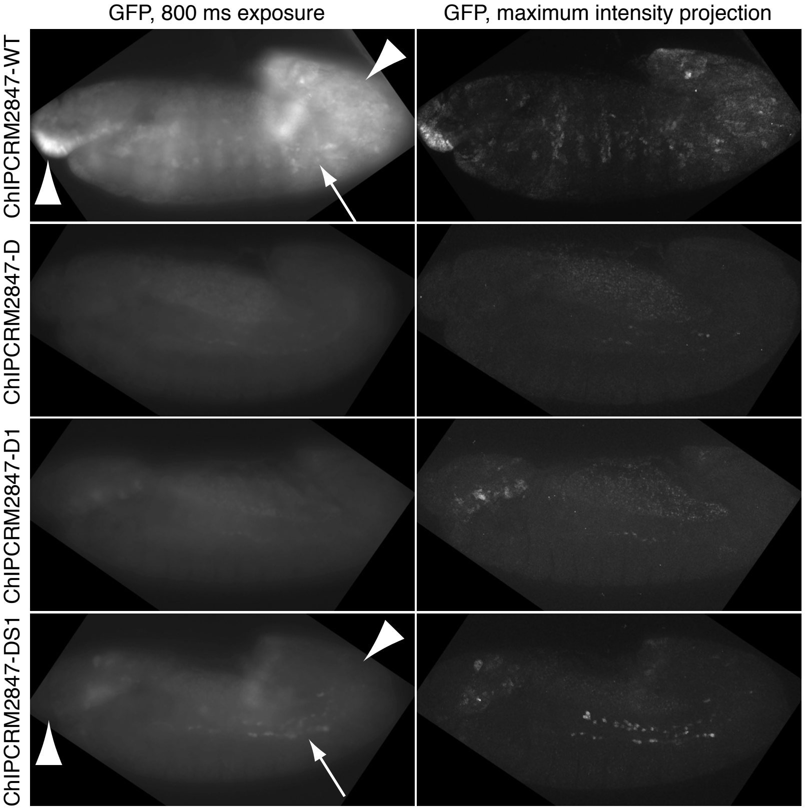

Fig. S20. Imaging of representative embryos from the ChIPCRM2847 series. The left column shows representative age-matched embryos, fixed, stained, and imaged in parallel, with identical exposure and with no adjustment to brightness or contrast, so that intensity can be compared. The right column shows reporter GFP expression in maximum intensity projections from a Z-series of structured illumination optical sections from the identical embryo, to permit the careful inspection of GFP expression pattern. Note that mutation of the single Schlank site substantially rescues the reduction of reporter expression caused by Deaf1 and CG12236-PB site mutations in one population of cells (arrows) but not in others (arrowheads).

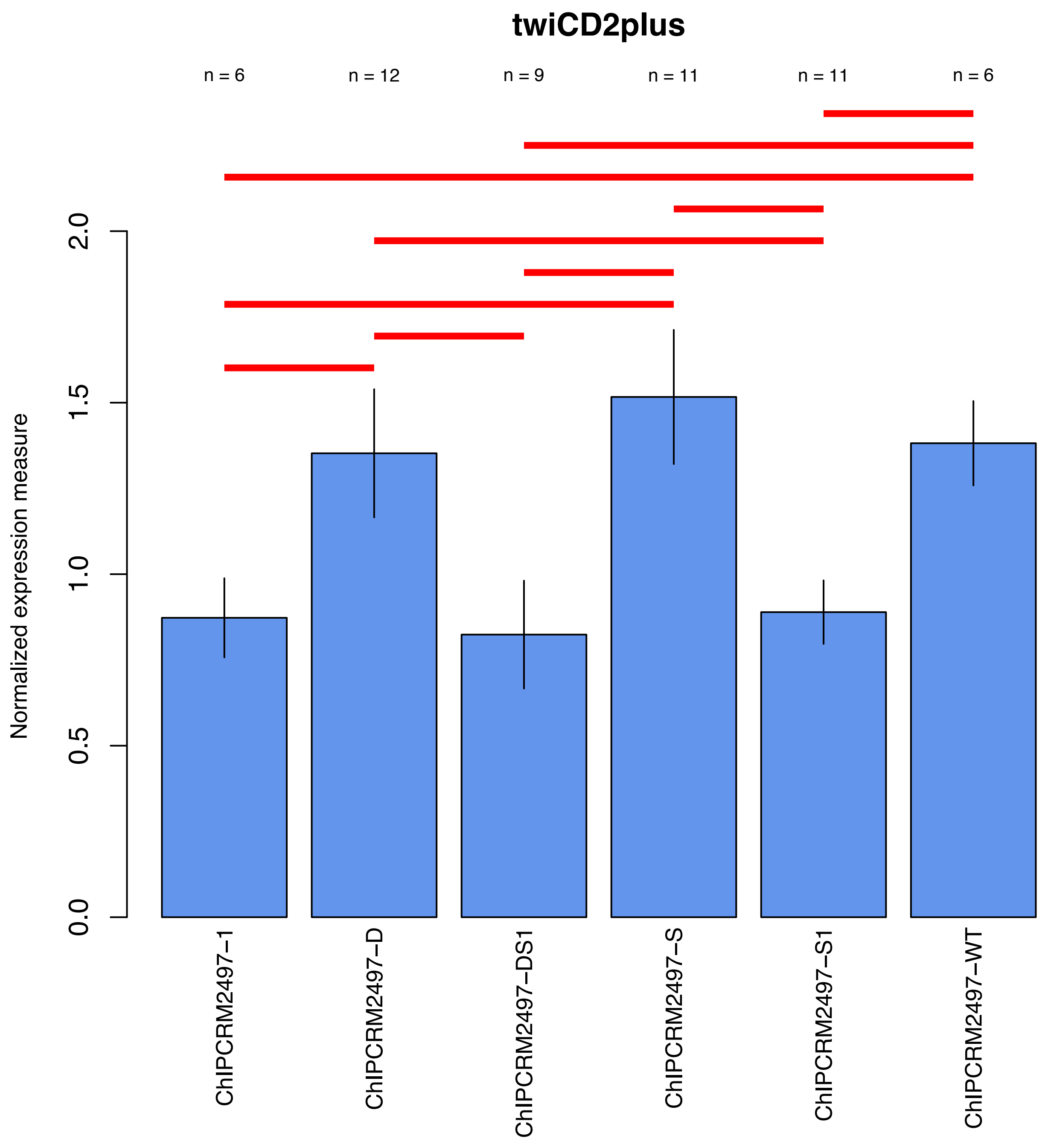

Fig. S21. Absolute expression measurements for ChIPCRM2497 series in twiCD2+ cells. Blue bars and error bars represent mean ± s.e.m. of the normalized expression measure (RNA UMI count / DNA UMI count) in all replicates (from independent collections and/or independent tagged constructs; number of replicates is provided above) for each enhancer. Red bars represent statistically significant differences (adjusted P-value < 0.05, Conover-Iman post-hoc test with Benjamini-Hochberg correction) between enhancer constructs.

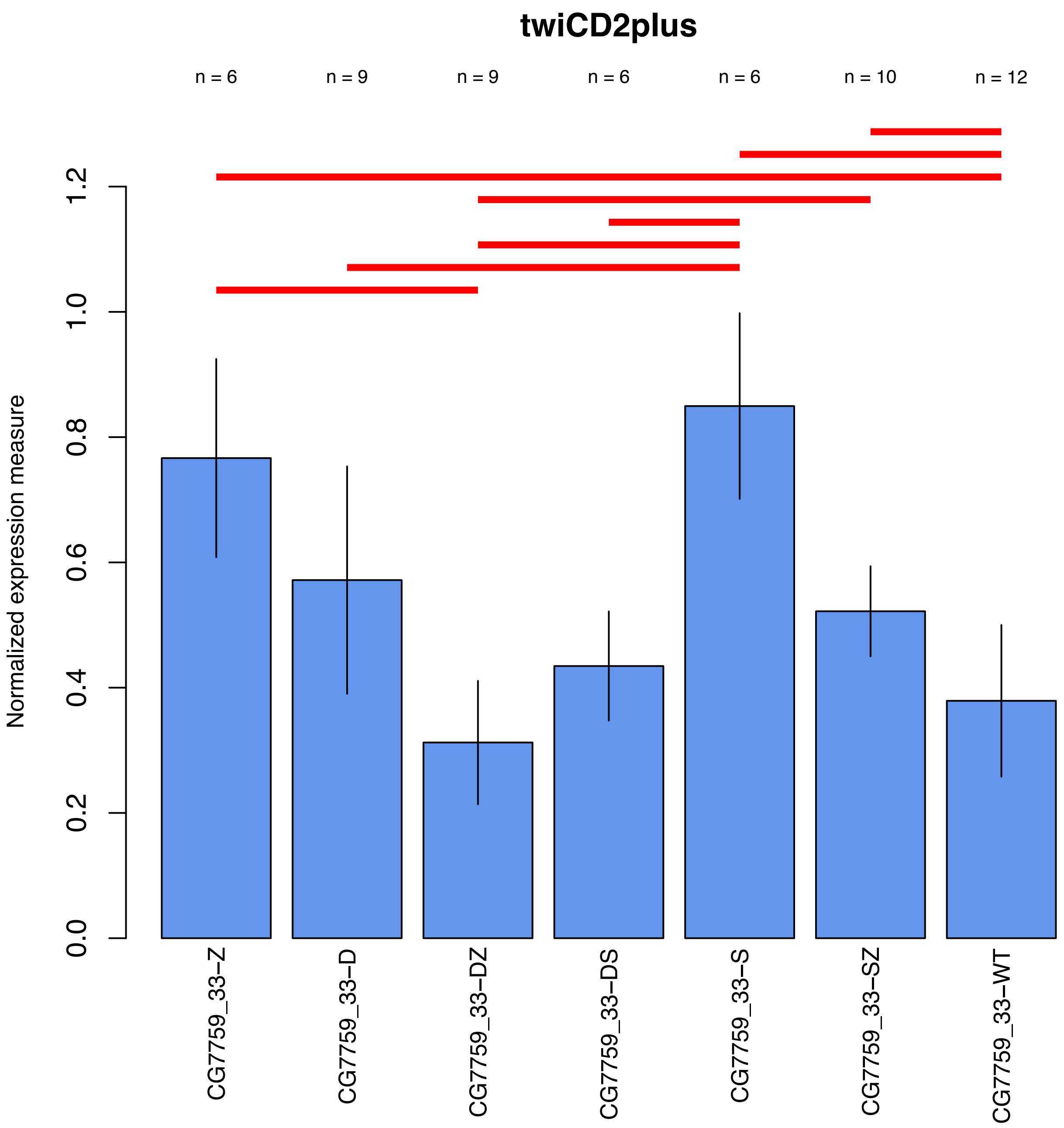

Fig. S22. Absolute expression measurements for CG7759_33 series in twiCD2+ cells. Blue bars and error bars represent mean ± s.e.m. of the normalized expression measure (RNA UMI count / DNA UMI count) in all replicates (from independent collections and/or independent tagged constructs; number of replicates is provided above) for each enhancer. Red bars represent statistically significant differences (adjusted P-value < 0.05, Conover-Iman post-hoc test with Benjamini-Hochberg correction) between enhancer constructs.

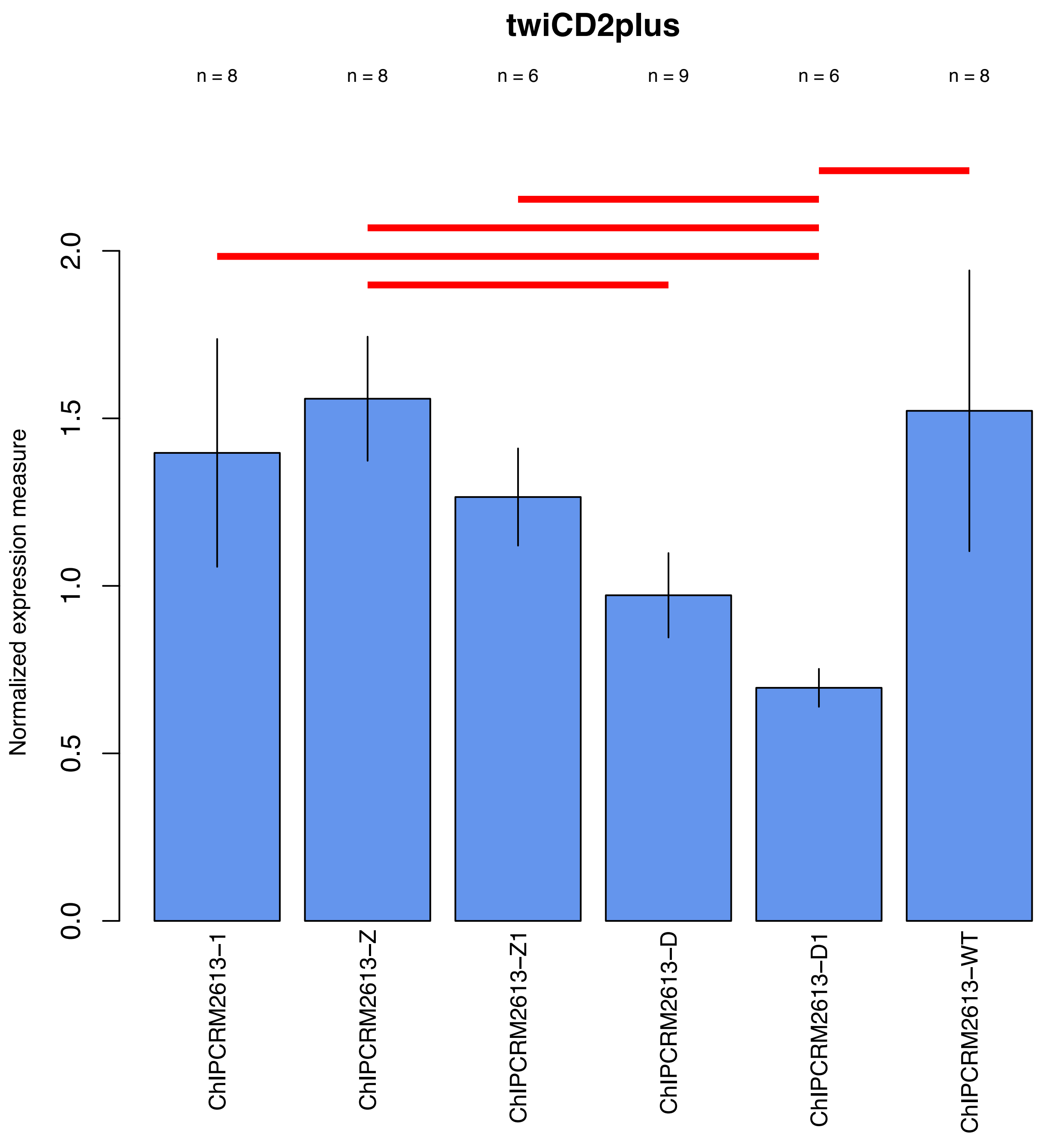

Fig. S23. Absolute expression measurements for ChIPCRM2613 series in twiCD2+ cells. Blue bars and error bars represent mean ± s.e.m. of the normalized expression measure (RNA UMI count / DNA UMI count) in all replicates (from independent collections and/or independent tagged constructs; number of replicates is provided above) for each enhancer. Red bars represent statistically significant differences (adjusted P-value < 0.05, Conover-Iman post-hoc test with Benjamini-Hochberg correction) between enhancer constructs.

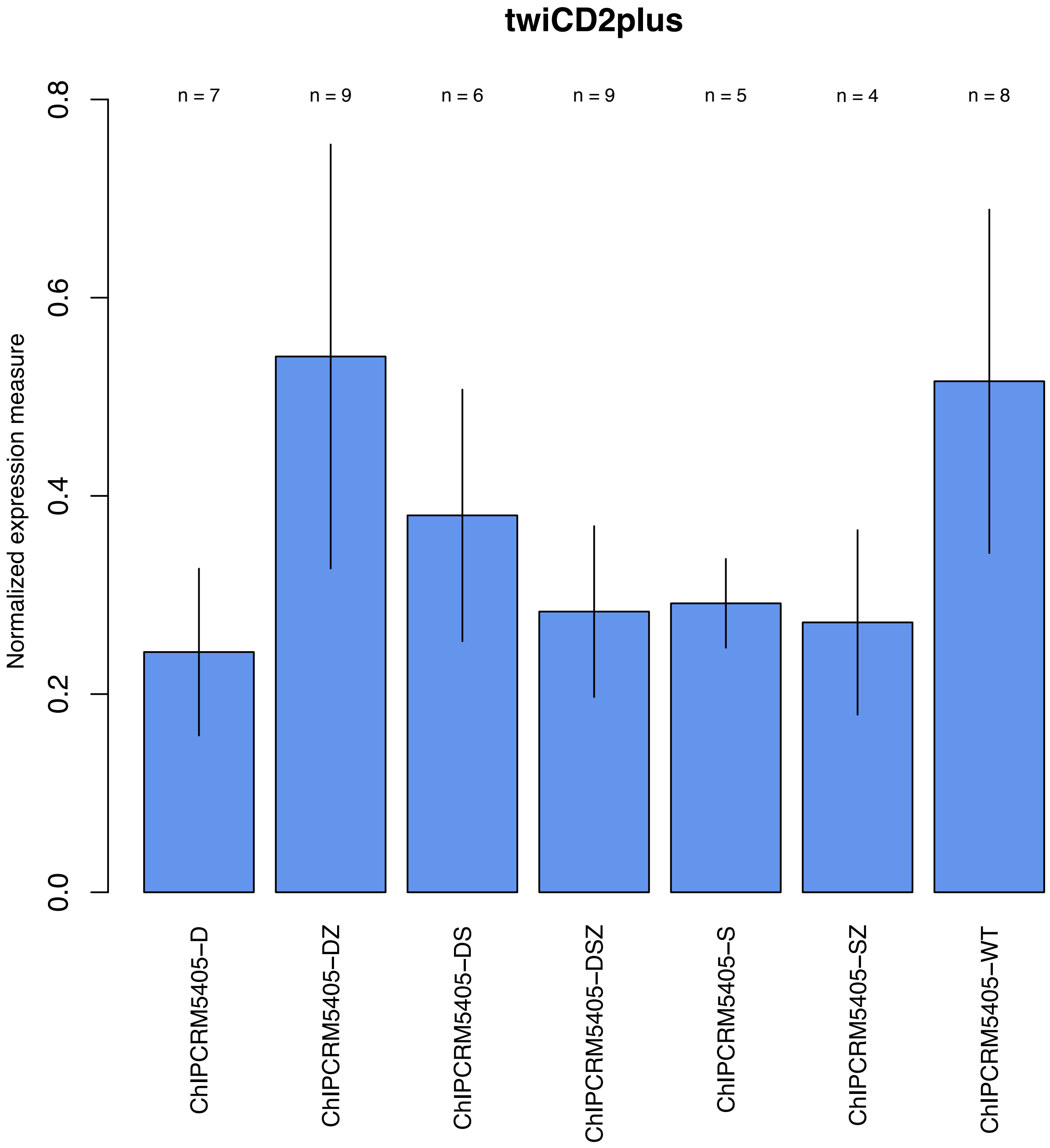

Fig. S24. Absolute expression measurements for ChIPCRM5405 series in twiCD2+ cells. Blue bars and error bars represent mean ± s.e.m. of the normalized expression measure (RNA UMI count / DNA UMI count) in all replicates (from independent collections and/or independent tagged constructs; number of replicates is provided above) for each enhancer. No statistically significant differences (adjusted P-value < 0.05, Conover-Iman post-hoc test with Benjamini-Hochberg correction) between enhancer constructs were detected.

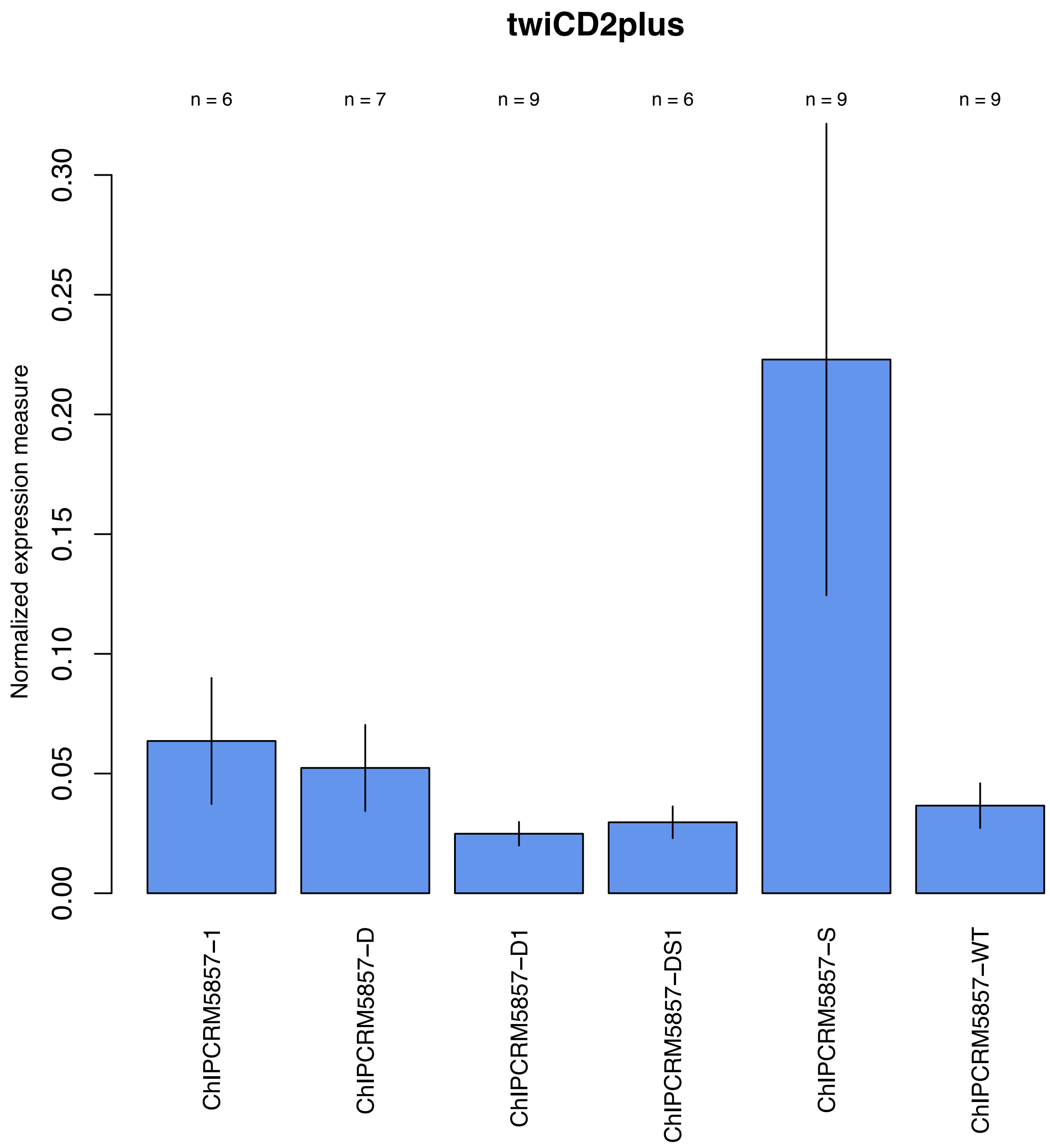

Fig. S25. Absolute expression measurements for ChIPCRM5857 series in twiCD2+ cells. Blue bars and error bars represent mean ± s.e.m. of the normalized expression measure (RNA UMI count / DNA UMI count) in all replicates (from independent collections and/or independent tagged constructs; number of replicates is provided above) for each enhancer. No statistically significant differences (adjusted P-value < 0.05, Conover-Iman post-hoc test with Benjamini-Hochberg correction) between enhancer constructs were detected.

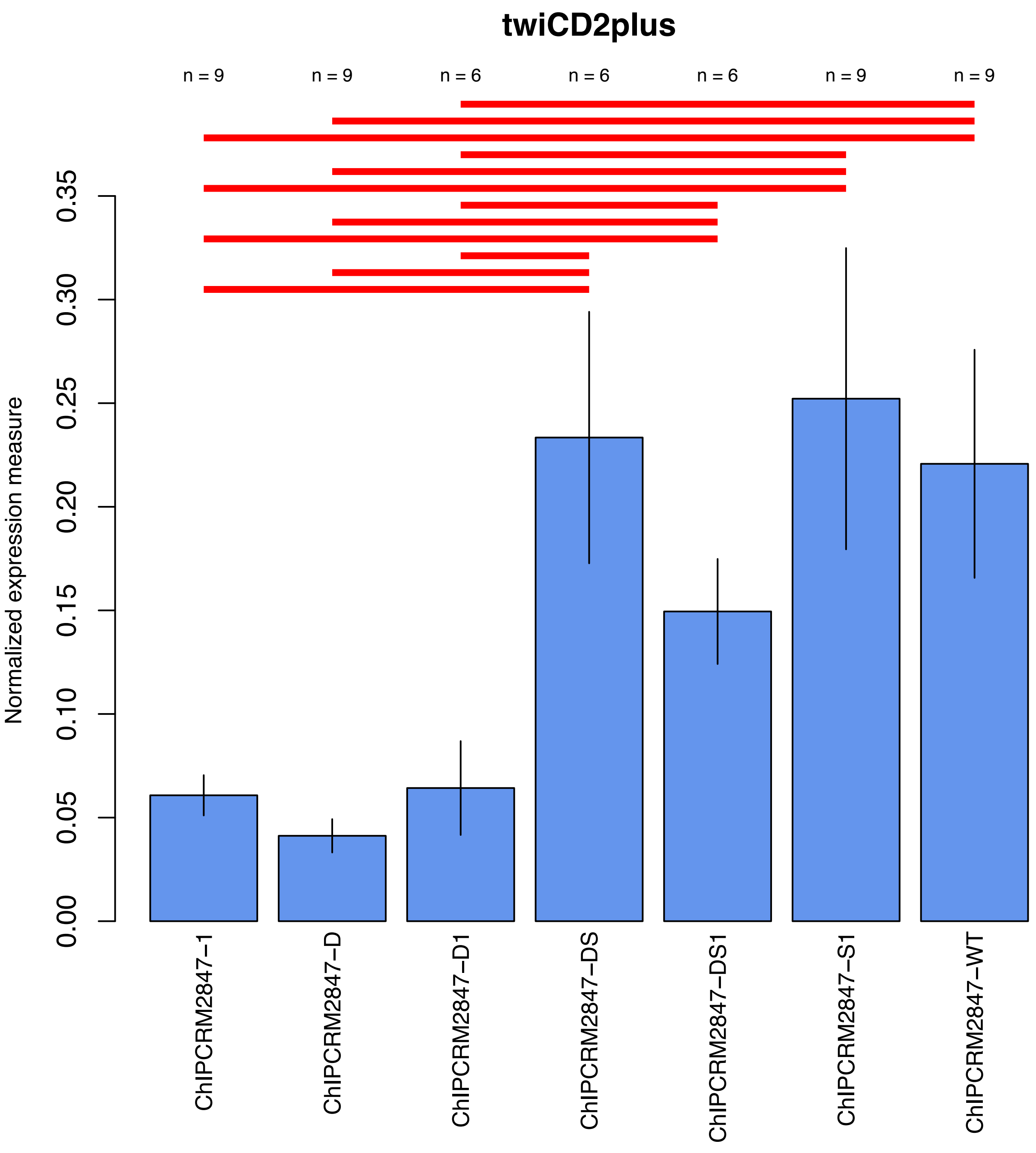

Fig. S26. Absolute expression measurements for ChIPCRM2847 series in twiCD2+ cells. Blue bars and error bars represent mean ± s.e.m. of the normalized expression measure (RNA UMI count / DNA UMI count) in all replicates (from independent collections and/or independent tagged constructs; number of replicates is provided above) for each enhancer. Red bars represent statistically significant differences (adjusted P-value < 0.05, Conover-Iman post-hoc test with Benjamini-Hochberg correction) between enhancer constructs.

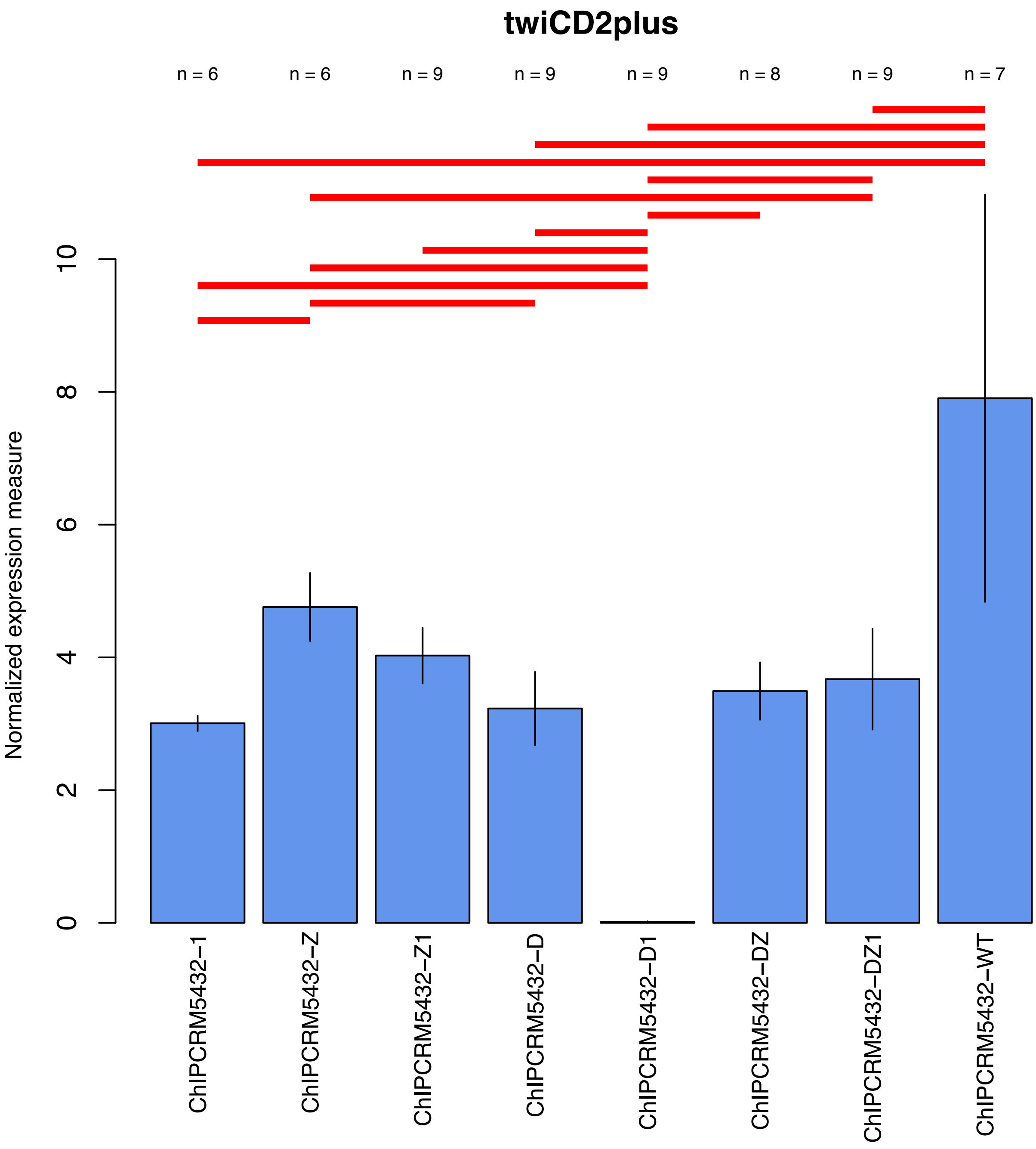

Fig. S27. Absolute expression measurements for ChIPCRM5432 series in twiCD2+ cells. Blue bars and error bars represent mean ± s.e.m. of the normalized expression measure (RNA UMI count / DNA UMI count) in all replicates (from independent collections and/or independent tagged constructs; number of replicates is provided above) for each enhancer. Red bars represent statistically significant differences (adjusted P-value < 0.05, Conover-Iman post-hoc test with Benjamini-Hochberg correction) between enhancer constructs.

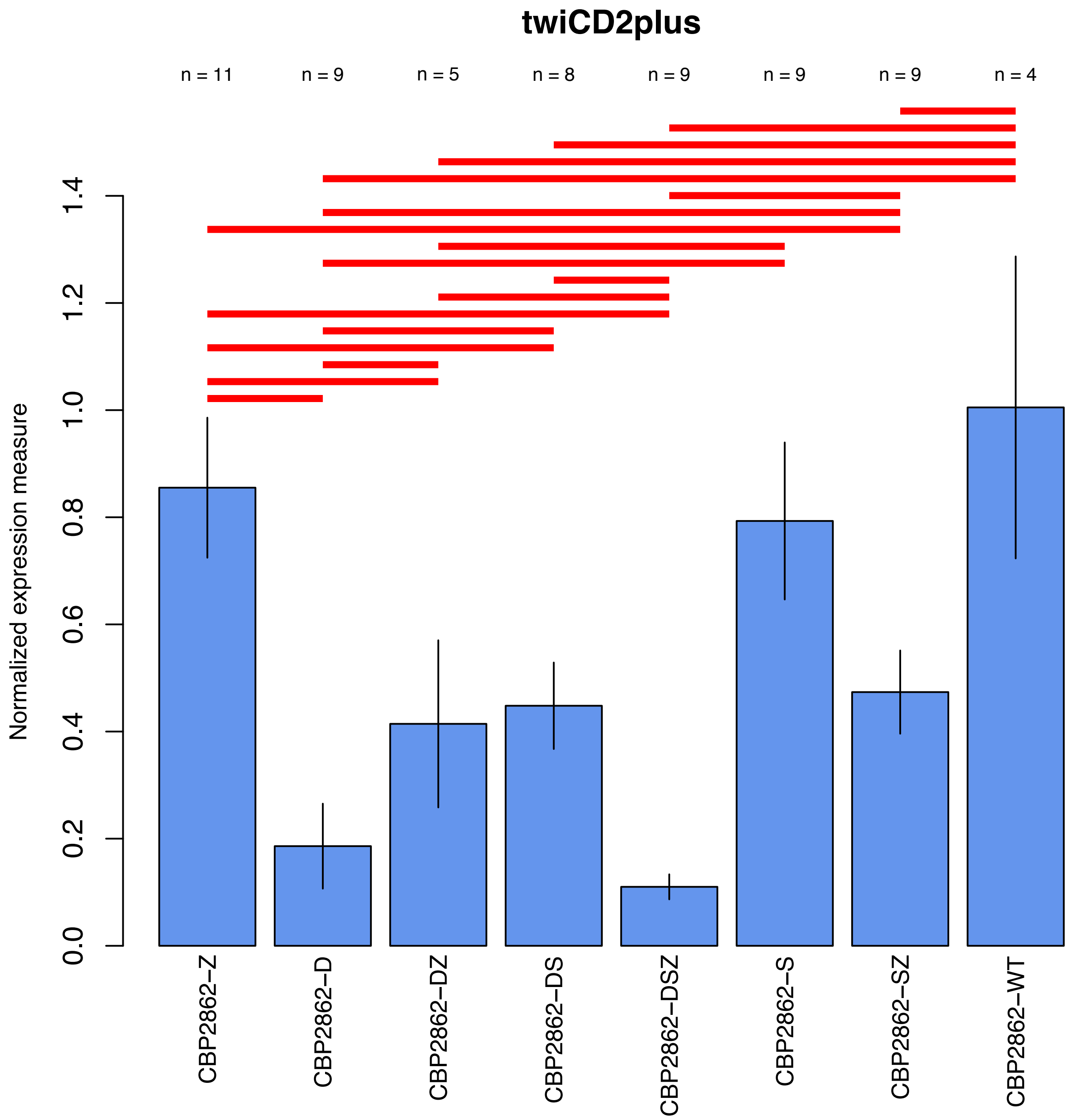

Fig. S28. Absolute expression measurements for CBP2862 series in twiCD2+ cells. Blue bars and error bars represent mean ± s.e.m. of the normalized expression measure (RNA UMI count / DNA UMI count) in all replicates (from independent collections and/or independent tagged constructs; number of replicates is provided above) for each enhancer. Red bars represent statistically significant differences (adjusted P-value < 0.05, Conover-Iman post-hoc test with Benjamini-Hochberg correction) between enhancer constructs.

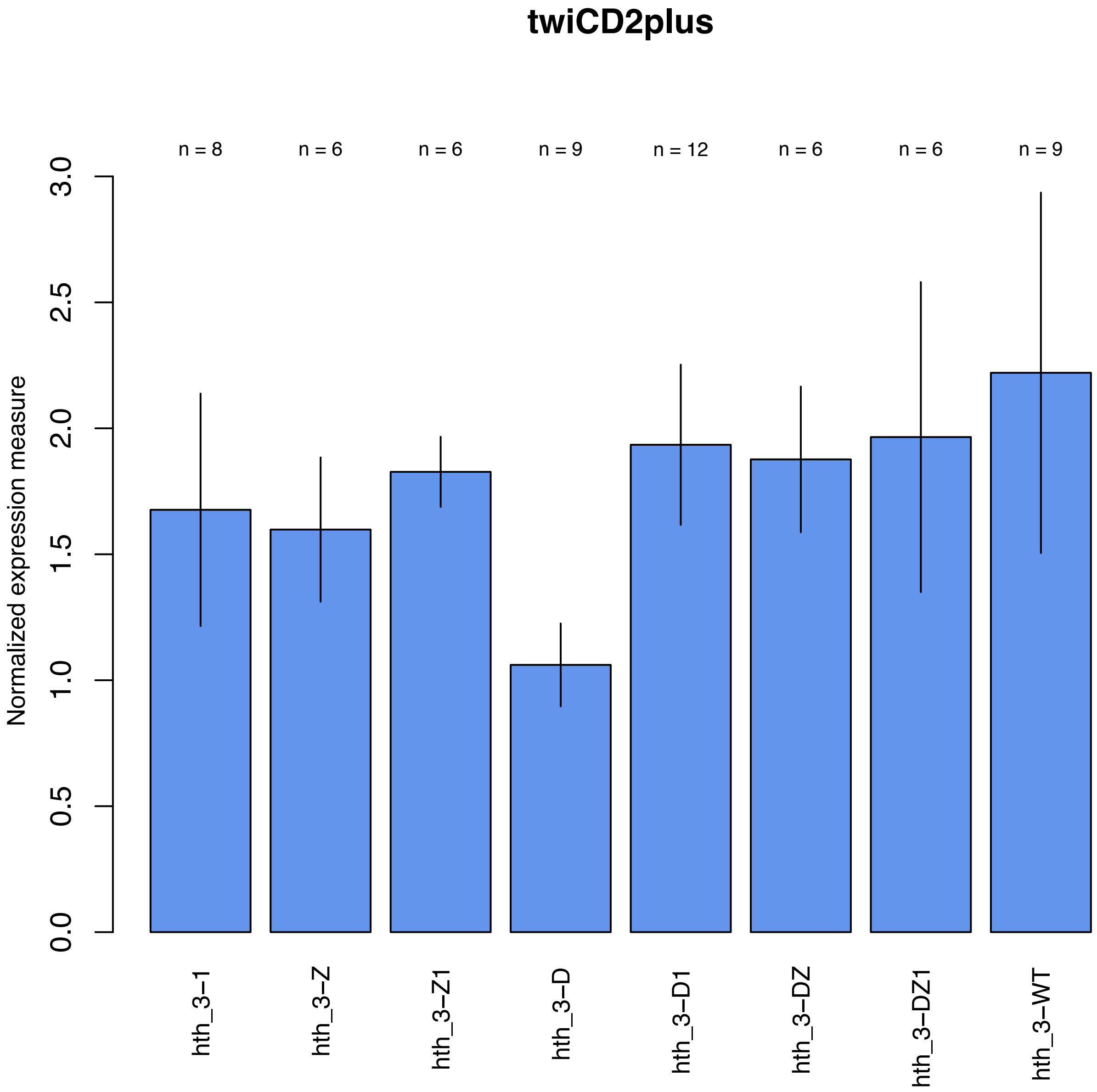

Fig. S29. Absolute expression measurements for hth_3 series in twiCD2+ cells. Blue bars and error bars represent mean ± s.e.m. of the normalized expression measure (RNA UMI count / DNA UMI count) in all replicates (from independent collections and/or independent tagged constructs; number of replicates is provided above) for each enhancer. No statistically significant differences (adjusted P-value < 0.05, Conover-Iman post-hoc test with Benjamini-Hochberg correction) between enhancer constructs were detected.

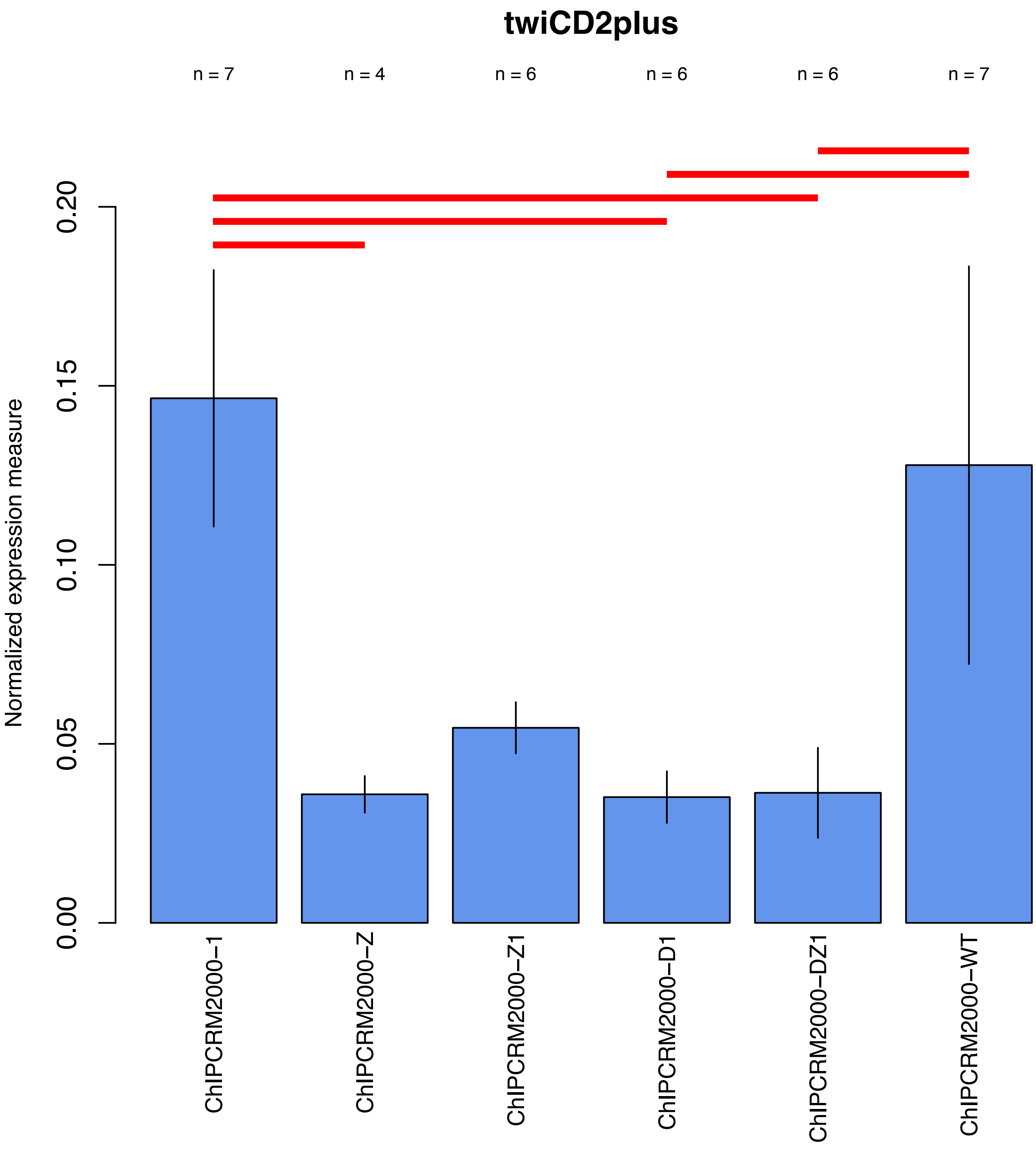

Fig. S30. Absolute expression measurements for ChIPCRM2000 series in twiCD2+ cells. Blue bars and error bars represent mean ± s.e.m. of the normalized expression measure (RNA UMI count / DNA UMI count) in all replicates (from independent collections and/or independent tagged constructs; number of replicates is provided above) for each enhancer. Red bars represent statistically significant differences (adjusted P-value < 0.05, Conover-Iman post-hoc test with Benjamini-Hochberg correction) between enhancer constructs.

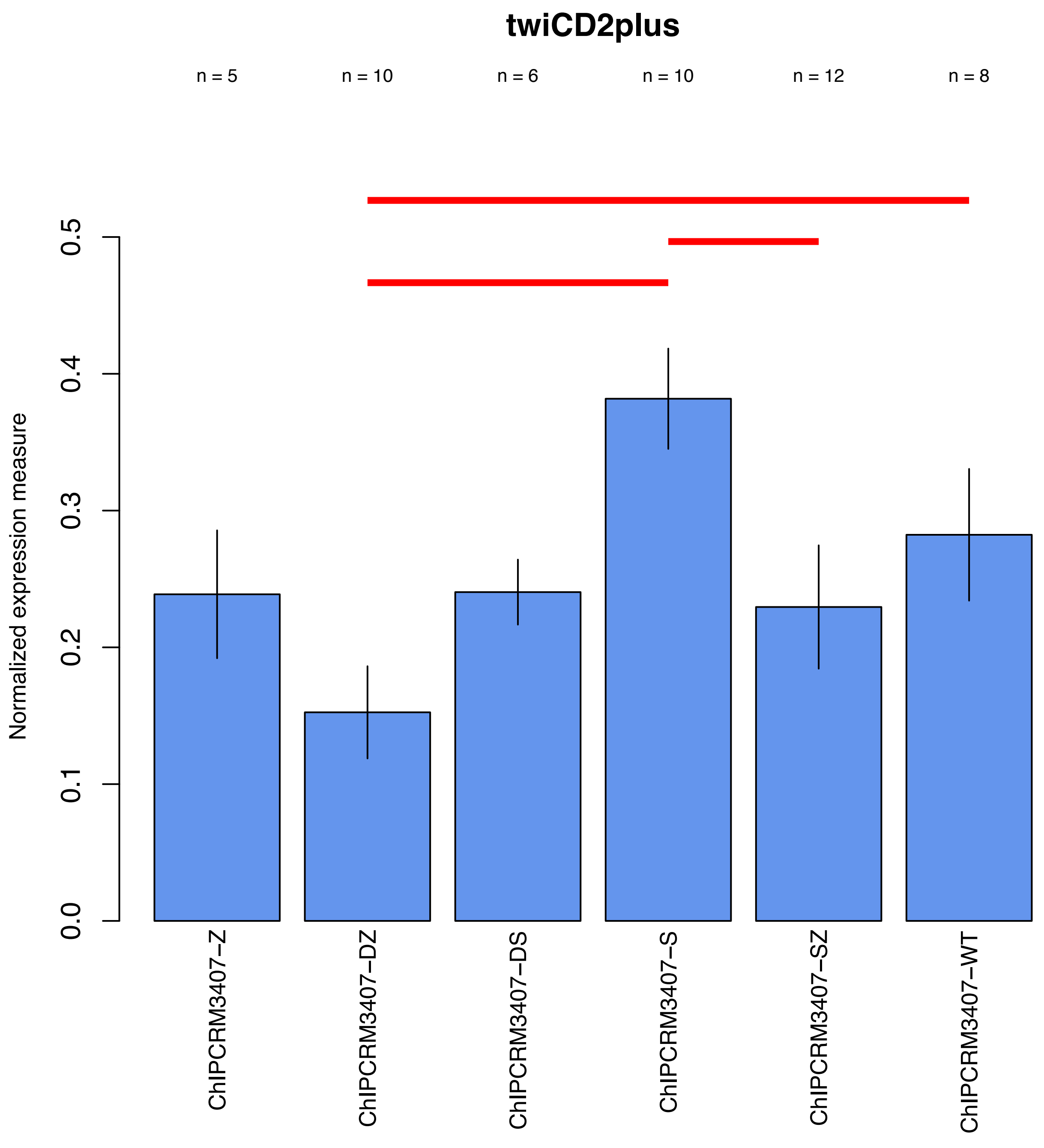

Fig. S31. Absolute expression measurements for ChIPCRM3407 series in twiCD2+ cells. Blue bars and error bars represent mean ± s.e.m. of the normalized expression measure (RNA UMI count / DNA UMI count) in all replicates (from independent collections and/or independent tagged constructs; number of replicates is provided above) for each enhancer. Red bars represent statistically significant differences (adjusted P-value < 0.05, Conover-Iman post-hoc test with Benjamini-Hochberg correction) between enhancer constructs.

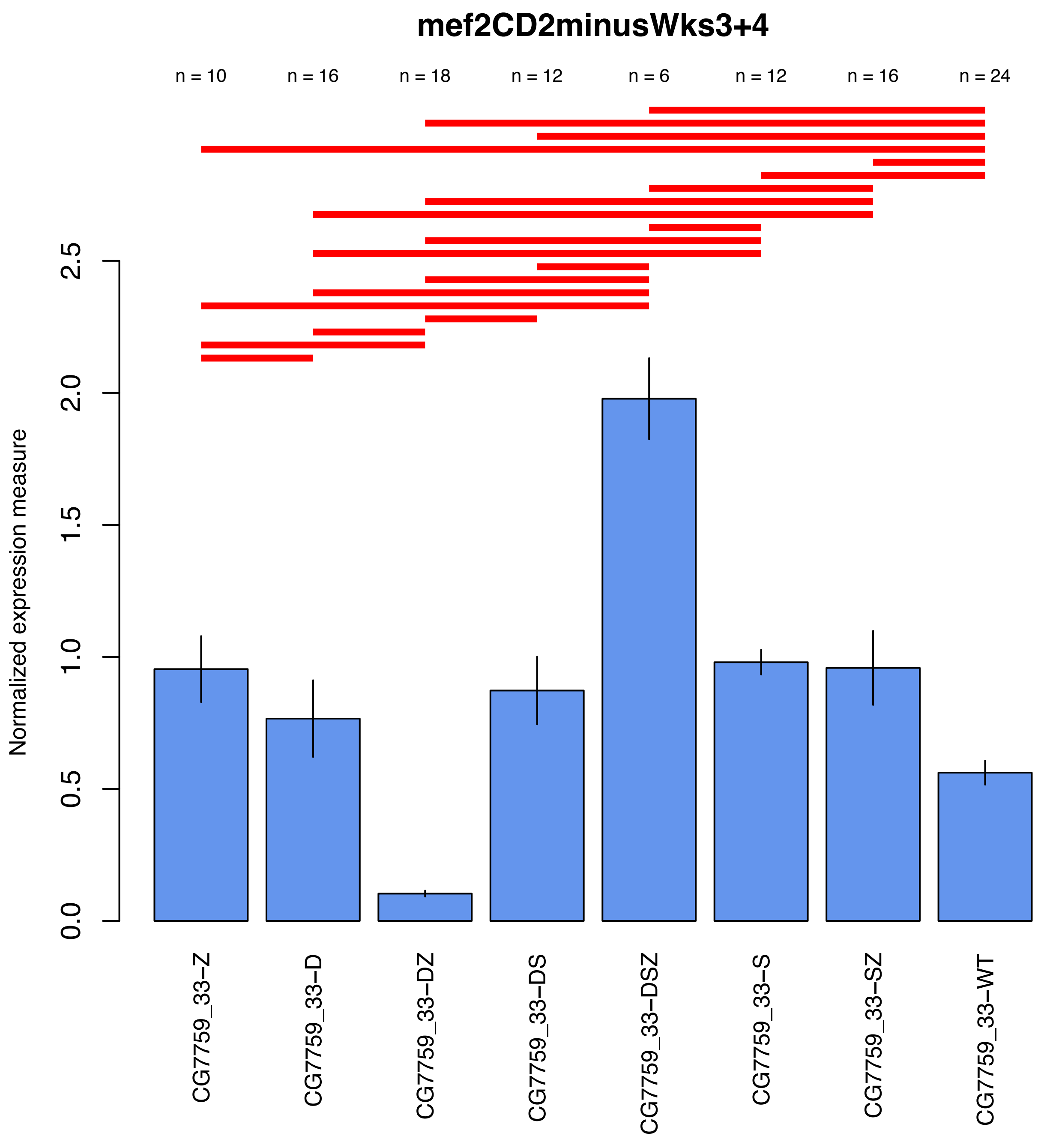

Fig. S32. Absolute expression measurements for CG7759_33 series in mef2CD2- cells. Blue bars and error bars represent mean ± s.e.m. of the normalized expression measure (RNA UMI count / DNA UMI count) in all replicates (from independent collections and/or independent tagged constructs; number of replicates is provided above) for each enhancer. Red bars represent statistically significant differences (adjusted P-value < 0.05, Conover-Iman post-hoc test with Benjamini-Hochberg correction) between enhancer constructs.
