# Supplementary material for: Quantitative-enhancer-FACS-seq (QeFS) reveals epistatic interactions among motifs within transcriptional enhancers in developing Drosophila tissue
Source: Genome Biol. 2021 Dec 20;22:348. doi: 10.1186/s13059-021-02574-x (PMC8686523; doi:10.1186/s13059-021-02574-x)
Supplement: Supplementary file 2 — Additional file 2: Supplemental Methods and Tables S1 and S2. [file 13059_2021_2574_MOESM2_ESM.docx]

**Supplemental Material** for “Quantitative-enhancer-FACS-seq (QeFS) reveals epistatic interactions among motifs within transcriptional enhancers in developing *Drosophila* tissue”

Colin T. Waters^1,2,6^, Stephen S. Gisselbrecht^1,6^, Yuliya A. Sytnikova^1^, Tiziana M. Cafarelli^3,4^, David E. Hill^3,4^, Martha L. Bulyk^1-3,5^

^1^ Division of Genetics, Department of Medicine, Brigham and Women’s Hospital and Harvard Medical School, Boston, MA 02115, USA.

^2^ Program in Biological and Biomedical Sciences, Harvard University, Cambridge, MA 02138, USA.

^3^ Center for Cancer Systems Biology, Dana-Farber Cancer Institute, Boston, MA 02215, USA.

^4^ Department of Cancer Biology, Dana-Farber Cancer Institute, Boston, MA 02215, USA and Department of Genetics, Harvard Medical School, Boston, MA 02115, USA.

^5^ Department of Pathology, Brigham and Women’s Hospital and Harvard Medical School, Boston, MA 02115, USA.

^6^ These authors contributed equally to this work.

Corresponding author: mlbulyk@genetics.med.harvard.edu

**Table of Contents**

**Supplemental Methods p. 3-12**

**Table S1 p. 6**

**Table S2 p. 10**

**Supplemental Methods**

**pQeFS vector construction**

Several modifications to the original pEFS vector were necessary to enable pQeFS. To enable efficient vector modification, a ligation-free, PCR-based cloning strategy known as FastCloning [59] was used. Using this method, a pair of primers were designed with homology to the 3’ UTR of the GFP reporter. Upstream of the 3’ UTR homology region of the primer is the desired insert (~20 to 120 bp). Thus, the forward primer is structured (5’ to 3’): insert, 3’ UTR homology and the reverse primer is structured (5’ to 3’): reverse complement of the insert, 3’ UTR homology. PCR with these primers amplifies the entire >10 kb plasmid, adding the short insert onto the 5’ ends of each amplicon. PCR reactions were treated with DpnI to digest any template plasmid, and the linear products with complementary ends were cloned into *E. coli* competent cells for propagation. Recircularization of the plasmid was not necessary prior to *E. coli* transformation.

This approach was used to introduce a short insert in the upstream region of the 3’ UTR (**Fig. S2**) that contained (5’ to 3’): a primer binding site (primer SQ7), a BmtI restriction enzyme site, a short spacer DNA, an AvrII restriction enzyme site, and a four base “handle.” Following FastCloning insertion, the plasmid was double-digested to remove the short spacer, and a long (~2.5 kb) region of *E. coli* DNA was ligated into its place. This recircularized vector was purified and propagated in *E. coli*. This region later served as the insertion site for the degenerate 20-mer tag that was used to distinguish reporter transcripts driven by different enhancers. The large insert was necessary to enable gel purification of the final ligation product from the precursor plasmid, as the degenerate 20-mer insert is substantially smaller than the 2.5 kb region of *E. coli* DNA, but would have been indistinguishable from the insert introduced in the earlier FastCloning PCR step. The precursor pQeFS vector (**Fig. S1**) was transformed into *E. coli*, midiprepped, and eluted at a final concentration of 0.64 ug/uL.

To ensure that the modifications to the pEFS backbone did not affect reporter activity, a pair of enhancers were cloned into pQeFS, and fly strains were created for each. These fly strains were crossed to the *twi*:CD2 fly line and embryonic expression in the progeny was assessed using fluorescence microscopy. A comparison to previously obtained images for these reporters in the pEFS backbone did not reveal any major differences in expression pattern (**Fig. S3**, panel A, C vs. B, D, respectively).

**Parallel cloning of enhancers into reporter backbones**

To prepare the library of enhancer-reporters, all enhancers were first cloned into pDONR223 in parallel, and then subsequently cloned into the pQeFS entry vector and screened individually. To obtain sufficient starting material for BP cloning, and to obviate the need for linearization of the pUC19 vectors, all enhancers were amplified out of pUC19 using Phusion polymerase, a standard three-step protocol, and universal M13R and M13F primers. Half-scale BP reactions for each enhancer amplicon were then performed in 96-well round-bottom reaction plates. pDONR223 was used instead of pDONR221 to enable spectinomycin selection and prevent persistence of Amp/Carb resistant pUC19 plasmids at later stages. Two microliters of each reaction was combined with 8 uL of lab-prepped DH5 alpha competent cells, incubated on ice for 30 minutes, heat shocked at 42 °C for 45 seconds, allowed to recover with the addition of 80 uL SOC medium, and incubated at 37 °C for 60 minutes. A QPix colony picking robot was then used to plate 20 uL of each transformation per chamber of a 48-chamber LB agar-spectinomycin plate, and plates were incubated at 37 °C overnight. The remaining transformation mix was stored at 4 °C overnight.

Following transformation, 81 of 100 cloning reactions yielded colonies. Two colonies from each transformation were picked and each was used to inoculate 150 uL of liquid TB with spectinomycin. Liquid cultures were arrayed in 96-well deep-well blocks, covered with a permeable plate seal and incubated at 37 °C overnight with shaking at 800 rpm. For the transformations which did not have overnight growth, the previous day’s transformation mix was spun at 3000 rpm for 15 minutes, and all but 15 uL of the supernatant was aspirated. Cell pellets were resuspended in the remaining medium and the entire transformation mix was plated in one chamber of an agar spectinomycin plate. These plates were incubated overnight at 37 °C, and all of the reactions yielded colonies. Colonies were chosen from these replated samples, and as above, used to inoculate liquid TB spectinomycin medium and incubated overnight. Following overnight growth, a Qiagen liquid handling robot was used to miniprep these samples in parallel. PCR screening of miniprepped material was performed, using KAPA2G or KOD polymerase, standard protocols, and M13R and M13F to identify successful transformants. This sequence of BP cloning and PCR screening was performed iteratively and all but one desired enhancer was successfully cloned, as determined by PCR screening.

In parallel with cloning of the enhancer elements, a large scale prep of the ligated pQeFS-dsN20 reporter was prepared. Five parallel transformations of pQeFS-dsN20 into ccdB survival cells were performed according to manufacturer’s instructions. Each transformation reaction was split into two following recovery in 2x SOC medium. Each half reaction was plated on a 15-cm LB carbenicillin plate (total of 10 plates), and the plates were incubated at 37 °C overnight. Colonies were scraped off the plates using a cell lifter in 1 mL TE pH 8.0 per plate, pooled, and processed with a Qiagen MaxiPrep kit. The MaxiPrepped material was eluted in a ~250 uL final volume at a concentration of ~2 ug/uL. Note that the solid culture approach was chosen to restrict the likelihood of runaway growth of individual dsN20-pQEFS elements in an attempt to maintain library complexity.

LR reactions were performed with BP reaction PCR products and a fraction of the dsN20-pQEFS maxiprep pool. As before, 2 uL of each reaction was used to transform 10 uL of DH5 alpha chemically competent cells. Transformation reactions were plated on LB carbenicillin plates and 5 colonies from overnight growth were chosen (where possible) for overnight growth in liquid culture. 96-well minipreps were performed using a Qiagen liquid-handling robot. Miniprepped material was then screened using the GFP-qPCR-F primer to assess the presence of a degenerate 20-mer tag, and the nestF primer to assess for expected enhancer presence. DNA yield from the liquid handling robot was variable, and often below the concentration required for Sanger sequencing, thus sequencing results were somewhat variable, and failure to yield results indicating unsuccessful cloning was confounded by unsuccessful sequencing. Nevertheless, screening of ~1000 miniprepped constructs identified 360 potential library elements with positive results for enhancers and tags. This required several iterations of cloning and screening, as we wanted to obtain coverage of each enhancer element with at least 3 unique degenerate barcodes. Overall, we obtained the complexity presented in **Table S1**.

**Table S1: Number of tags obtained for enhancers in the screen.**

| Number of tags | Number of constructs |
| --- | --- |
| 0 | 8 (1 Gen9, 1 BP, 6 LR failures) |
| 2 | 7 |
| 3 | 38 |
| 4 | 23 |
| 5 | 15 |
| 6 | 5 |
| 7 | 5 |

All constructs were quantified by NanoDrop, and pooled such that each enhancer would be present in the library at equal concentration scaled to the number of unique tagged elements present in the library. Library elements were pooled manually, attempting to work with volumes greater than 2 uL to reduce pipetting error. The final pooled library (volume ~6 mL) was concentrated on a rotary evaporator, extracted, precipitated, and resuspended in 300 uL of 1X fly embryo injection buffer.

**Preparation of the barcode-tag lookup table library**

1.5 ug of precipitated, washed library in 1X injection buffer was removed for construction of the look-up table library linking degenerate tags to enhancer barcodes (**Fig. S4, S5**). This sample was Bmt I digested, excising the bulk of the GFP reporter and the enhancer region, and then recircularized by ligation to juxtapose the reporter tag and enhancer barcode. To limit runaway amplification of the library, a trial PCR was performed using a dilution series of input material, and 13 total cycles of amplification were chosen for the final amplification step with primers P5_SQ3 (AATGATACGGCGACCACCGAGATCTGTACAAAAAAGCAGGCTCAAGACGAGGCTATGCTCTAGC) and P7_IndexN_IndexSeq_SQ6 (CAAGCAGAAGACGGCATACGAGAT-index-GTGACTGGAGTTCAGACGTGTGCTCTTCCGATCTTAAGGCTAGAGTTTGCCTGCTC). The library was then purified using a single-sided SPRI selection with a ratio of 0.8x beads:sample. The library was sequenced on an Illumina MiSeq with a 70 base read 1 run and a 6 base index 1 read with the LUT_sequencing custom sequencing primer (GTACAAAAAAGCAGGCTCAAGACGAGGCTATGCTCTAGC). A total of 35,247,345 raw reads were sequenced, and 33,876,153 Passed Filter reads were identified, a rate of 96%. A total of 33,322,759 reads had the expected Illumina index.

Following lookup table library preparation and sequencing, a total of 31,898,661 tag counts were obtained, distributed across 221,158 barcode-tag matches for 98 enhancers. Although, as desired, the distribution of enhancer frequency in this library was fairly uniform (**Fig. S6**), we also observed redundant degenerate tags matched to multiple enhancer barcodes. In addition to the desired intramolecular ligations during look-up table library prep, there are apparent instances of intermolecular ligation, leading to spurious barcode-tag matches. These spurious pairs did not affect our eventual experimental results, as individual reporter tags are physically linked to the enhancers that drive their expression, but they did affect the creation of the lookup table, potentially impacting our ability to map reporter tags back to the enhancers that drive their expression in an unambiguous manner. To investigate the origin of these spurious barcode-tag pairs in more detail, tags for a given enhancer were clustered according to Levenshtein distance, which accounts for insertions, deletions, and substitutions between two strings. For example, for the enhancer ChIPCRM5405-S, clustering of all the observed degenerate tags followed by tree-cutting to 320 groups (based on the number of expected barcode-tag pairs) revealed two predominant clusters and 318 other low-frequency groups (**Fig. S7**). Each of the two predominant clusters contained variants of one or the other of the expected tags for this enhancer. Thus, they represented potential sequencing errors, or mutations introduced during the library preparation step. The other clusters correspond to expected tags for other enhancer barcodes and were assumed to be derived from spurious intermolecular ligations. In general, we expect that clusters with many members correspond to a true barcode-tag pair, while minority clusters are spurious pairings. Thus, for each enhancer, a consensus sequence can be generated for each predominant tag cluster to obtain the barcode-tag pair. Mismatches that may be present in tags in the actual enhancer-reporter library can be dealt with at later analysis stages by allowing mismatches when mapping reporter RNA-seq reads back to tags in the look-up table, as described below. By virtue of having cloned this library in parallel, we also had a set of expected barcode-tag pairs. In this filtered, expected set, 26,891,515 tag counts were observed, distributed across 320 barcode-tag matches for 94 enhancers.

Using the expected barcode-tag lookup table, we sought to determine the per-enhancer tag distribution; in other words: how evenly distributed was a barcode’s total abundance across its constituent tags? Overall the distribution was fairly even (**Fig. S8**), although there are examples where a single tag dominates, likely due to a combination of quantification and pipetting errors during library pooling.

**Injection details**

For injections, 275 uL of 1.43 ug/uL plasmid library were submitted to Rainbow Transgenic Flies for injections. Thirty rounds of injection were performed over a tight time-window of approximately 10 days, and 6375 injections were performed, of which 3951 larvae hatched, for a viability rate of 62%. This viability rate is consistent with previous commercial injections.

**Fly husbandry and FACS**

The QeFS library was injected into flies from the strain *w- nos-ΦC31; attP40*. This strain drives the ΦC31 integrase under control of the *nanos* promoter leading to expression during oogenesis, resulting in mRNA deposition in the egg prior to fertilization. This strain also contains an attP integration site (recognized by the ΦC31 integrase) on the second chromosome. Recombination between the attP site in the fly genome and the attB site present in the pQeFS vector leads to stable integration of the enhancer-reporter into the fly genome and conversion of the recognition sites into attL and attR sites, thus preventing further recombination events. Note that the attB/P and attL/R sites for Gateway cloning are separate and distinct from the ΦC31 integrase recognition sites. Thus, in the germline of the injected embryo, two integration events are possible in each primordial germ cell, and the haploid gametes will contain a single integrated enhancer-reporter (if any), thus the progeny of each gamete will have a single enhancer-reporter element in the genome of every cell, and successful transformant progeny will be marked by *w+*.

Viable larvae from injected embryos were reared to adulthood, and male flies were crossed with *yw* virgin females. Transformed, *w+* male progeny from this cross were then crossed to virgin females from a strain with cell type-specific expression of a rat CD2 cell-surface marker. This cell type-specific expression of the cell-surface marker enables purification of a given cell type of interest through the use of FACS. In principle, any fly population of interest could be purified using this method, as long as a cell type-specific enhancer is available. For our experiments, we used the promoter region for the TF *twist* (*twi)*, which enables isolation of whole mesoderm at embryonic stage 11 and 12. We also used the *Mef2-I-E_D5_* enhancer for purification of fusion-competent myoblasts, a mesodermal subset from the same developmental stage. This cross between virgin females from the CD2 driver line and male progeny from the injected population was performed in 10-cm diameter population cages. Flies were allowed to lay eggs on molasses plates for two 2 hour collections. After each laying period, plates were aged for 10-11 hours at 18 °C or 5.5 hours at 25 °C, to obtain embryos in developmental stage 11-12. After aging, embryos were collected off plates, dechorionated, and single cell-suspensions were prepared for FACS. Collections were performed each morning on Wednesday, Thursday, and Friday. Two weeks of collections were performed for *twi:*CD2 crosses followed by two weeks for *mef2:*CD2 crosses.

Single cell suspensions were stained with Alexa647 conjugated anti-Rat CD2 antibody (AbDSerotec. #MCA154A647) diluted 1:40 in Schneider medium with 2 ug/mL DAPI stain, and washed once in Schneider’s medium with 8% FBS. Cell suspensions for embryos from *yw* and *twi:*CD2 or *mef2:*CD2 flies were prepared at the same time to serve as FACS controls. During FACS, gates were first set using *yw* cells to account for auto-fluorescence and to identify live and dead cells, and gates were then set on the CD2 driver line to distinguish CD2+ and CD2- populations. Cells were then sorted using these gates (**Figure S9**). This yielded approximately 2 to 4 million cells per collection for *twi*:CD2+ cells (**Table S2**). Both collections for each day were pooled, spun down gently, resuspended in 500 uL TRIzol, and flash frozen in liquid nitrogen before storage at -20 C.

**Table S2. Cell counts for FACS collections.**

| Date (CD2 driver) | Early collection |  | Later collection |  |
| --- | --- | --- | --- | --- |
|  | CD2+ | CD2- | CD2+ | CD2- |
| 10-25-17 (*twi*) | 2609K | 546K | 2465K | 444K |
| 10-26-17 (*twi*) | 2131K | 530K | 1848K | 476K |
| 10-27-17 (*twi*) | 1651K | 477K | 1587K | 463K |
|  |  |  |  |  |
| 11-01-17 (*mef2*) | 78K | 4282K | 78K | 5034K |
| 11-02-17 (*mef2*) | 50K | 3121K | 46K | 3261k |
| 11-03-17 (*mef2*) | 132K | 6934K | 95K | 5478K |
|  |  |  |  |  |
| 11-08-17 (*mef2*) | 78K | 5847K | 52K | 5256K |
| 11-09-17 (*mef2*) | 62K | 5422K | 71K | 5227K |
| 11-10-17 (*mef2*) | 77K | 4580K | 91K | 5247K |

**Library preparation**

Since we believed there would be a range of enhancer-driven expression, including the possibility of enhancers with either no activity or undetectable activity, we sought to simultaneously purify reporter transcript RNAs (giving a read-out of activity) and the reporter DNA elements (giving a read-out of the relative abundance of each enhancer in the population of cells). With these data, reporter transcripts could be scaled by overall reporter abundance, providing a normalization factor for the signal and enabling the identification of enhancers which were inactive or undetectable. For each sample in TRIzol, RNA was purified using standard acid phenol:chloroform extraction of the aqueous phase, followed by concentration on a ZYMO RNA Clean and Concentrator 5 column. DNA was purified by double back extraction of the organic phase using TNES-6U buffer (10 mM Tris-Cl, pH 7.5, 125 mM NaCl, 10 mM EDTA pH 8.0, 1% SDS, 6 M Urea), followed by phenol:chloroform extraction and ethanol precipitation.

An important consideration in the design of custom sequencing approaches is the ability to successfully distinguish clusters on the Illumina sequencing chip. If there is insufficient nucleotide complexity within the first ~6 cycles of sequencing, cluster identification by the Illumina processing software fails, and overall yield can suffer dramatically or the run can fail entirely. To address this, we incorporated a UMI tag into the library preparation primers, which would not only introduce complexity into the first few bases sequenced, but also enable direct quantification of RNA transcripts, thus controlling for the potential introduction of bias that can occur through runaway amplification during library construction.

The overall strategy was as follows (**Fig. S10**): simultaneously purify RNA and DNA from the samples in TRIzol using the back extraction method for DNA, TURBO DNase treat the RNA fraction to eliminate any contaminating DNA, purify on a Zymo RNA Clean and Concentrator 5 column, and reverse transcribe the RNA using a nested primer (QeFS_RT_oligo, GTTCAGAGTTCTACAGTCCGA). After reverse transcription and cDNA purification, a single round of primer extension was performed to label the second strand with the UMI tag, this was performed with KAPA polymerase and a primer that runs (5’ to 3’): Illumina P5 cluster generation primer, Illumina read 1 sequencing primer, 8-base degenerate UMI sequence, QeFS complement (P5_Rd1_UMI_QeFS, AATGATACGGCGACCACCGAGATCTACACTCTTTCCCTACACGACGCTCTTCCGATCTNNNNNNNNCGCGGGATGCTAGCACGCGG). This primer incorporated both the Illumina P5 cluster generation primer and the standard Illumina sequencing primer. Sequencing begins with the 8-base UMI sequence, then runs through a 20-base constant sequence, and finally into the 20-base degenerate tag. Following primer extension, the second-strand DNA was purified using a 0.9x beads:sample SPRI purification, and PCR was performed using P7_IndexN_IndexSeq_SQ6, which binds internally to the QeFS_RT_oligo, and a universal primer that contains the Illumina P5 sequence and the Illumina read 1 sequencing primer (AATGATACGGCGACCACCGAGATCTACACTCTTTCCCTACACGACGCTCTTCCGATCT). This universal primer maintained the UMI tag present on each second strand DNA and in combination with the 0.9x SPRI purification, prevented rewriting of UMI tags or retagging of cDNAs. The structure of the resulting amplicon is shown in **Fig. S11**.

UMI counts for DNA and RNA, or the final scaled expression measurement were not heavily biased by the distribution of tags in the initial input library (**Fig. S13**). Correlating the tag counts from the lookup table with any of these three measures returns a Pearson’s r of -0.07 to 0.09 for the first day of the *twi*:CD2+ collections. Although we did not see an overall correlation of enhancer representation in the experimental library with the input (injected) library, we also did not see full representation of the input library in the experimental library. For instance, our lookup table library (a proxy for the injected library) contained 320 tags, but the largest number of tags present on any given day in the experimental library is 287, suggesting that there were dropout events that occured due to variation in the fly population over the month-long experiment.

Although the trends are largely consistent between the two weeks, there was some variability in which changes are called as significant, likely due to variability in tag representation between the two weeks (due to changes in the fly population over time) and to differences in sequencing depth. For example, in the week 1 collection, a significant loss in activity was observed following Deaf1 site mutations in ChIPCRM5432 (*P* = 0.028), while a loss of activity was also observed in the week 2 collection, it was not called significant by the Conover-Iman test.
