# Supplementary material for: Quantitative-enhancer-FACS-seq (QeFS) reveals epistatic interactions among motifs within transcriptional enhancers in developing Drosophila tissue
Source: Genome Biol. 2021 Dec 20;22:348. doi: 10.1186/s13059-021-02574-x (PMC8686523; doi:10.1186/s13059-021-02574-x)
Supplement: Supplementary file 5 — Additional file 5: Peer review history. [file 13059_2021_2574_MOESM5_ESM.docx]

**Review history**

**First round of review**

**Reviewer 1**

Summary
In this manuscript Waters and colleagues develop Quantitative enhancer-FACS-Seq (QeFS), a quantitative variant of their previous eFS method (Gisselbrecht, Nat Methods 2013). They then use QeFS for the parallel quantification of enhancer activities from a genomically integrated reporter in Drosophila embryos to uncover the genetic interactions among TF motifs present within enhancers. Specifically, they characterise the contribution of the motifs of 4 TFs individually and in combination to the activity of 12 embryonic mesodermal enhancers, using a series of enhancer variants with motif mutations. The authors put forward a range of epistatic interactions among the motifs and propose that understanding the contribution of TF motifs to enhancer activity requires that they be investigated both individually and in combination across different contexts.
Quantifying enhancer activities in the context of whole organisms and specific tissues has been difficult due to the lack of quantitative techniques. For example, enhancer activity in Drosophila has been traditionally assessed using in situ hybridization (Erceg, PLoS Genetics 2014), while quantitative methods (like STARR-seq and other MPRAs) are usually performed in cell lines (Arnold, Science 2013; Melnikov, Nat Biot 2012). MPRAs have already been performed in the mouse liver, using tail vein injection (Patwardhan, Nat Biot 2012; Smith, Nat Gen 2013) and in Ciona (Farley, Science 2015), but this approach is not easily applicable to other tissues or whole organisms.
Despite the importance of the question and the need for such technologies, the current manuscript provides weak evidence for the robustness, quantitativeness and cell type specificity of QeFS and the corresponding analyses and validations have to be substantially extended. In addition, the results on motif-mutant variants of 12 enhancers are misrepresented as the data typically don't show consistent results for individual mutations of TF motifs and only anecdotical evidence of different epistatic interactions between TFs (see below). The authors need to substantially revise the discussion of their results and extend the validations to a more systematic set of tests.

Main comments

1 - The authors use only whole-embryo qRT-PCR for the validation of the mesodermal enhancer activities and the putative mesoderm-specific mutation effects. This might be ok for wildtype enhancers if their strictly mesoderm activity could be ensured (e.g. based on the literature). However, the non-cell type specificity of the assay could invalidate the results of the motif mutations as they can change enhancer activities in the mesoderm but also elsewhere (see e.g. Farley, Science 2015). For example, the increase in activity shown in Fig. 3 for some mutations could be due to increased enhancer activity in a non-mesodermal tissue and no changes in mesoderm.

Similarly, the results presented in Fig. 2 (pages 8-9) do not "assess the sensitivity and cell-type specificity of QeFS", as the authors claim, because QeFS and qRT-PCR data are not performed on the same cells and QeFS seems to have been performed in mef2:CD2- cells (as a proxy for measurements from whole embryos) but not in mesoderm-specific cells. For a correct validation and cell-type specificity assessment, the QeFS and qRT-PCR data should be compared for the same cells, e.g. in twi:CD2+ and twi:CD2- cells for the 2 positive control enhancers (Fig. 2).

2 - It is difficult to assess the quantitativeness of the method since the authors only show "fold change vs. WT" without providing a sense of the actual enhancer activity levels behind the fold-changes (i.e. the enhancer activities of WT and mutant enhancer sequences). This is even more important for interpreting the effect of combinatorial motif mutations. For example, it is possible that a 0.2 fold-change of a motif mutation (Fig. 4D) results in very low absolute enhancer activity levels such that the impact of mutating an additional motif cannot be assessed. This could provide an alternative explanation for the apparent redundancy of effects in Fig. 4D and invalidate the authors' claim. Therefore, the authors should show the absolute enhancer activities of WT and mutant enhancer sequences.

3 - Due to the low number (12) of enhancers studied and because there are no consistent results for individual mutations of TF motifs and only anecdotical evidence of different epistatic interactions between TFs, the main scientific claims of the paper appear overstated (see also point 4). Since the authors selected enhancers containing always 3 out of the 4 TF motifs, systematically comparing enhancers with the same trio of TF motifs might reveal if there are consistent effects or not.

We therefore suggest comparing the results (from both individual and combinatorial mutations) between enhancers with similar motif composition (containing the same TF motif types) to assess how consistent the findings are. For example, can the motif composition of the enhancers explain why only some are dependent on loss of Deaf1 or CG12236-PB? Is there any interaction/epistasis between specific TF partners that is consistent between more than one enhancer? Without such a comparison that might explain the variability of the outcomes, the results appear stochastic.

4 - Fig. 3: The statements describing the results of individual motif mutations in the text are misleading as they typically only represent a minority of enhancers. The authors should add the number of enhancers with significant effects and revise the sentences according with the results.
- Page 10: "Loss of Deaf1 sites typically led to decreased enhancer activity in whole mesoderm". This is misleading since only 3 out of 9 enhancers showed significant reductions (Fig 3A).
- Page 10: "Mutation of CG12236-PB sites also reduced enhancer activity". Add to the text "3 out of 7 enhancers".
- Page 11: "the QeFS data revealed increases in enhancer activity resulting from mutation of ZIPIC or Schlank binding sites". Again, this sentence is misleading since only one enhancer is significantly affected for each (out of 7 for ZIPIC and of 6 for Schlank), actually being the same enhancer for both. The authors argue that "Loss of ZIPIC or Schlank binding sites results in context-dependent increases in enhancer" (section title), but this is based on a single outlier out of 7 tested enhancers. Please revise the sentences and the title of the section.

5 - Fig.3: The authors only test the QeFS results for 4 significant mutations by whole-embryo qRT-PCR (of which 3 validate). For example, in Fig. 3A/B only 2 out of 9 results are validated and in Figs. 3D/E and G/H only 1 out of 7. Given the variable motif mutation effects across enhancers per TF, the manuscript would benefit from the validation of more mutation effects. This should include mutations with no impact on enhancer activity to validate these are not false-negatives of the QeFS method (see Fig. 3 A, D and G).


Other comments

1 - The Figure legends miss important information to interpret the figures. Please add to all legends the number of measurements used in each quantification and what the error bars represent.

2 - Page 7: it would be useful for the reader to mention the lengths of the 12 selected enhancers, since the enhancer length was an argument used by the authors when motivating the new technique.

3 - Page 10: A reference to Fig 3E is missing and should be included, ideally with a brief discussion as the qPCR result does not validate the QeFS result.

4 - Fig 2 and 3: to clarify from which cell type or embryo (for qPCR) is the data from, please add to figure titles the cell type, similar to Fig. 4.
- For example, in Fig 2A,B, show in figure that the QeFS data is from mef2:CD2-, and therefore a proxy of whole embryo. Otherwise, it can be misled as mesodermal-specific.
- Fig 2C,D and 3B,E,H: show in figure that the qPCR data is from whole embryo, otherwise it can be misled as mesodermal-specific validation data.

5 - Fig. S16: why are only 11 enhancers shown and not 12?

6 - Fig. S16: The colour legend is not correct. It does not include all the colours shown in the heatmaps (e.g. blue) neither negative fold-changes.

**Reviewer 2**

Article summary:

Waters and Gisselbrecht et al. modified a previous technique known as eFS to provide an RNA-Seq readout for a tested enhancer of interest. The group carried out this modified technique: Quantitative enhancer-FACS-Seq (QeFS) to decipher the enhancer logic of mesodermal enhancers containing binding sites for Deaf1, Schlank, ZIPIC, and CG12236-PB. These binding sites were previously identified to be involved in mesoderm development using eFS. As a proof of concept, the scientists tested 88 mesodermal enhancers with these motifs using QeFS, mutated the sites combinatorially, and studied the enhancer readout. Waters and Gisselbrecht et al. found different forms of previously described epistatic interactions between these Transcription Factors, proposing that TF binding sites should be analyzed combinatorially.

Major comments:

One of the goals for this study is to increase the experimental throughput for studying enhancers. It is great to see many groups working on addressing this problem from different perspectives. Using QeFS, many barcoded enhancer lines can be integrated and tested directly in the Drosophila genome (and not as episomes) and screened based on RNA-Seq levels. Compared to eFS, QeFS allows the quantification of RNA levels and has an advantage over cell populations. The enhancer readout is now in the proper cellular context, and entire fields of cells could be quantified and expression patterns observed. Additionally, QeFS was used to explore how four TFs that are not well characterized control mesodermal development.

However, recent studies have screened much more than 88 enhancer lines using standard techniques (i.e., insertion and antibody staining / in-situ hybridization) (Kvon et al., Cell 2020 and Le Poul et al., Science Advances 2020) or other higher-throughput techniques (Fuqua et al., 2020). These studies additionally provide patterning information in addition to enhancer activity levels. From this perspective, the advantage QeFS has over other techniques is the RNA quantification, which is much more quantitative than the other methods introduced. I believe the "high-throughput" component of QeFS is plausible but not demonstrated in this manuscript and should be toned-down.

I was disappointed to see that the authors do not test the patterning of these enhancers. In my opinion, there is not as much insight gained from doing QeFS compared to standard techniques or using cell lines. I believe the study would become much more informative, strengthen its argument if the expression patterns were imaged - perhaps a subset from each epistatic category (synergistic, alleviating complementary, alleviating complete, and sign epistasis). This way, the authors could relate the mutant phenotypes to see why these different forms of epistasis are occurring.
Based on the current data, I found the epistasis argument hard to just. For example, it could be possible to have variable effects of modifying one site that would be cell-type specific in the embryo. At the same time, the other "epistatic" site may have variable effects in other cell-types. It would benefit from having staining for the inputs -- what cells are they expressed - and a subset of their collection imaged to explore the effects of the mutations. This approach would more directly address epistasis by controlling for cell-type-specific changes, pleiotropy, etc.

Overall, it is clear that the authors put much work into this technique and were diligent with their statistics and data analysis. The technique, QeFS, is an improvement upon a previous protocol, and it was used to identify TF interactions, which is (in my opinion) the more important part of this story. However, it would benefit from more imaging to of (at least) a subset of the reporter constructs from each category to potentially understand why the different forms of epistasis are observed.

Minor comments:

"Such epistatic interactions, where the regulatory output of a particular combination of motifs is different from that expected if the motifs are treated as acting independently, is important to identify since rules learned for individual motifs in one enhancer context might not be informative of those motifs' regulatory consequences in a different enhancer context." - This sentence is convoluted. Consider rewriting or splitting it into two.

"Massively parallel reporter assays (MPRAs) [21] and STARR-Seq [22] are powerful in their ability to assay tens to hundreds of thousands of short elements (typically less than 200 bp in length) and for their ability to drive expression of a reporter in cultured cells."

"To assay cis-regulatory elements for enhancer activity in a quantitative, highly parallel fashion in a chromosomal context in the context of particular tissues in whole Drosophila embryos, we developed quantitative enhancer-FACS-Seq (QeFS). In QeFS, each cis-regulatory element was cloned upstream of a reporter gene, where a in whose 3' UTR a degenerate sequence was inserted into the 3' UTR and used as an identifying tag for the RNA-Seq readout of enhancer activity (Additional file 1: Fig. S1, S2, S3)."

It would be great for the authors to add a comment on how the effects may extend to the native locus.

The authors should comment on how their results compare to the recent work by Fuqua et al. (Nature, 2020)?

In figure 3C, this is hard to interpret. It would be great if the authors could note what enhancer they came from - color code?

**Authors’ response to reviewers**

Reviewer #1: Summary
In this manuscript Waters and colleagues develop Quantitative enhancer-FACS-Seq (QeFS), a quantitative variant of their previous eFS method (Gisselbrecht, Nat Methods 2013). They then use QeFS for the parallel quantification of enhancer activities from a genomically integrated reporter in Drosophila embryos to uncover the genetic interactions among TF motifs present within enhancers. Specifically, they characterise the contribution of the motifs of 4 TFs individually and in combination to the activity of 12 embryonic mesodermal enhancers, using a series of enhancer variants with motif mutations. The authors put forward a range of epistatic interactions among the motifs and propose that understanding the contribution of TF motifs to enhancer activity requires that they be investigated both individually and in combination across different contexts.
Quantifying enhancer activities in the context of whole organisms and specific tissues has been difficult due to the lack of quantitative techniques. For example, enhancer activity in Drosophila has been traditionally assessed using in situ hybridization (Erceg, PLoS Genetics 2014), while quantitative methods (like STARR-seq and other MPRAs) are usually performed in cell lines (Arnold, Science 2013; Melnikov, Nat Biot 2012). MPRAs have already been performed in the mouse liver, using tail vein injection (Patwardhan, Nat Biot 2012; Smith, Nat Gen 2013) and in Ciona (Farley, Science 2015), but this approach is not easily applicable to other tissues or whole organisms.
Despite the importance of the question and the need for such technologies, the current manuscript provides weak evidence for the robustness, quantitativeness and cell type specificity of QeFS and the corresponding analyses and validations have to be substantially extended. In addition, the results on motif-mutant variants of 12 enhancers are misrepresented as the data typically don't show consistent results for individual mutations of TF motifs and only anecdotical evidence of different epistatic interactions between TFs (see below). The authors need to substantially revise the discussion of their results and extend the validations to a more systematic set of tests.

Main comments
 
1 - The authors use only whole-embryo qRT-PCR for the validation of the mesodermal enhancer activities and the putative mesoderm-specific mutation effects. This might be ok for wildtype enhancers if their strictly mesoderm activity could be ensured (e.g. based on the literature). However, the non-cell type specificity of the assay could invalidate the results of the motif mutations as they can change enhancer activities in the mesoderm but also elsewhere (see e.g. Farley, Science 2015). For example, the increase in activity shown in Fig. 3 for some mutations could be due to increased enhancer activity in a non-mesodermal tissue and no changes in mesoderm.

We recognize that the mixing of cell types is inherently a limitation of our method and have taken various steps to address it, including several in response to the helpful suggestions of the reviewers. In Figure 3, only data from sorted mesoderm is now shown, so that all comparisons are identical.

Similarly, the results presented in Fig. 2 (pages 8-9) do not "assess the sensitivity and cell-type specificity of QeFS", as the authors claim, because QeFS and qRT-PCR data are not performed on the same cells and QeFS seems to have been performed in mef2:CD2- cells (as a proxy for measurements from whole embryos) but not in mesoderm-specific cells. For a correct validation and cell-type specificity assessment, the QeFS and qRT-PCR data should be compared for the same cells, e.g. in twi:CD2+ and twi:CD2- cells for the 2 positive control enhancers (Fig. 2).

In the interest of brevity, we omitted any extensive discussion of the criteria by which we selected controls for inclusion in the library; we apologize for the resulting lack of clarity. These enhancers were chosen because they have previously been shown to drive expression only in the mesoderm, and because these exact site mutations do not create any ectopic expression domains, so that whole embryo qRT-PCR could be compared directly to mesodermal QeFS. We have added text to clarify this point.

Thereafter, all validation by whole embryo qRT-PCR is compared to data from *mef2*:CD2- cells. Moreover, we have attempted to address questions of cell type-specific effects in part by adding imaging of GFP expression patterns in representative embryos in Figures S16-S20, as suggested by Reviewer 2; we saw very few instances (now highlighted in the main body text) where different cell types responded differently to mutation of the same site.

2 - It is difficult to assess the quantitativeness of the method since the authors only show "fold change vs. WT" without providing a sense of the actual enhancer activity levels behind the fold-changes (i.e. the enhancer activities of WT and mutant enhancer sequences). This is even more important for interpreting the effect of combinatorial motif mutations. For example, it is possible that a 0.2 fold-change of a motif mutation (Fig. 4D) results in very low absolute enhancer activity levels such that the impact of mutating an additional motif cannot be assessed. This could provide an alternative explanation for the apparent redundancy of effects in Fig. 4D and invalidate the authors' claim. Therefore, the authors should show the absolute enhancer activities of WT and mutant enhancer sequences.

In our experience (we have shown versions of such figures to test-audiences), showing all WT and mutant expression values for multiple enhancers in the same main body figure makes a very confusing presentation; we have therefore added supplemental figures with this information for every main body figure panel in which fold-change bargraphs are shown (Figures S21-S32). Complete data for all comparisons, including expression level and number of replicates, are provided in Table S4. Notably, absolute expression level (as measured by QeFS) did not explain the failure to observe significant changes in some series; compare for example figures S24 and S29 to S30.

3 - Due to the low number (12) of enhancers studied and because there are no consistent results for individual mutations of TF motifs and only anecdotical evidence of different epistatic interactions between TFs, the main scientific claims of the paper appear overstated (see also point 4). Since the authors selected enhancers containing always 3 out of the 4 TF motifs, systematically comparing enhancers with the same trio of TF motifs might reveal if there are consistent effects or not.

We therefore suggest comparing the results (from both individual and combinatorial mutations) between enhancers with similar motif composition (containing the same TF motif types) to assess how consistent the findings are. For example, can the motif composition of the enhancers explain why only some are dependent on loss of Deaf1 or CG12236-PB? Is there any interaction/epistasis between specific TF partners that is consistent between more than one enhancer? Without such a comparison that might explain the variability of the outcomes, the results appear stochastic.

Such comparisons within groups were previously displayed in Figure S16. As the reviewer found this information poorly displayed in the previous draft, we have moved this to main body Figure 3 to highlight the extent to which such effects are and, more to the point, are not consistent between enhancer contexts. Each effect we show is supported by ≥ 4 replicate measurements of each enhancer construct, as detailed in new Figures S21-S32. We have added text on page 12 highlighting our reasons for assaying the Deaf1+CG12236-PB interaction in ChIPCRM2000, as shown in Figure 5B (originally 4C) and now also in Figure S17. More broadly, the fact that modes of epistasis even between the same two motifs tend not to be consistent across our sample of enhancer contexts is interesting to us, and we emphasize in our conclusions section that it challenges us as a field to gather more data and try to learn when we can and cannot generalize.

4 - Fig. 3: The statements describing the results of individual motif mutations in the text are misleading as they typically only represent a minority of enhancers. The authors should add the number of enhancers with significant effects and revise the sentences according with the results.
-       Page 10: "Loss of Deaf1 sites typically led to decreased enhancer activity in whole mesoderm".  This is misleading since only 3 out of 9 enhancers showed significant reductions (Fig 3A).
-       Page 10: "Mutation of CG12236-PB sites also reduced enhancer activity". Add to the text "3 out of 7 enhancers".
-       Page 11: "the QeFS data revealed increases in enhancer activity resulting from mutation of ZIPIC or Schlank binding sites". Again, this sentence is misleading since only one enhancer is significantly affected for each (out of 7 for ZIPIC and of 6 for Schlank), actually being the same enhancer for both. The authors argue that "Loss of ZIPIC or Schlank binding sites results in context-dependent increases in enhancer" (section title), but this is based on a single outlier out of 7 tested enhancers. Please revise the sentences and the title of the section.

Agreed. We have made all of these revisions in the revised manuscript.

5 - Fig.3: The authors only test the QeFS results for 4 significant mutations by whole-embryo qRT-PCR (of which 3 validate). For example, in Fig. 3A/B only 2 out of 9 results are validated and in Figs. 3D/E and G/H only 1 out of 7. Given the variable motif mutation effects across enhancers per TF, the manuscript would benefit from the validation of more mutation effects. This should include mutations with no impact on enhancer activity to validate these are not false-negatives of the QeFS method (see Fig. 3 A, D and G).

We apologize that the organization of our initial submission spread qRT-PCR validation across three different figures. In total, we have tested 17 mutant-vs-WT comparisons by this method. We have moved all qRT-PCR validations to a new Figure panel 2F to make this more clear, and to highlight the reliability and limitations of the method as compared to qRT-PCR.


Other comments
 
1 - The Figure legends miss important information to interpret the figures. Please add to all legends the number of measurements used in each quantification and what the error bars represent.

We have added information about the error bars and qRT-PCR replicates to main body figures, and information about QeFS replicates now appears in Figures S21-S32.

2 - Page 7: it would be useful for the reader to mention the lengths of the 12 selected enhancers, since the enhancer length was an argument used by the authors when motivating the new technique.

We have added this information on page 8.

3 - Page 10: A reference to Fig 3E is missing and should be included, ideally with a brief discussion as the qPCR result does not validate the QeFS result.

All qRT-PCR validations have now been collected in Figure 2.

4 - Fig 2 and 3: to clarify from which cell type or embryo (for qPCR) is the data from, please add to figure titles the cell type, similar to Fig. 4.
- For example, in Fig 2A,B, show in figure that the QeFS data is from mef2:CD2-, and therefore a proxy of whole embryo. Otherwise, it can be misled as mesodermal-specific.
- Fig 2C,D and 3B,E,H: show in figure that the qPCR data is from whole embryo, otherwise it can be misled as mesodermal-specific validation data.

Agreed. We have made all of these revisions in the revised figures.

5 - Fig. S16: why are only 11 enhancers shown and not 12?

This figure shows fold-change measurements vs. wild type, and no data passed QC on 1/12 of the wild type enhancer constructs, so no fold-changes could be calculated. This information has been added to the legend of what is now main body Figure 3.

6 - Fig. S16: The colour legend is not correct. It does not include all the colours shown in the heatmaps (e.g. blue) neither negative fold-changes.

Thank you. This was a weird artifact of moving between R and Illustrator and has now been corrected.


Reviewer #2:
Article summary:

Waters and Gisselbrecht et al. modified a previous technique known as eFS to provide an RNA-Seq readout for a tested enhancer of interest. The group carried out this modified technique: Quantitative enhancer-FACS-Seq (QeFS) to decipher the enhancer logic of mesodermal enhancers containing binding sites for Deaf1, Schlank, ZIPIC, and CG12236-PB. These binding sites were previously identified to be involved in mesoderm development using eFS. As a proof of concept, the scientists tested 88 mesodermal enhancers with these motifs using QeFS, mutated the sites combinatorially, and studied the enhancer readout. Waters and Gisselbrecht et al. found different forms of previously described epistatic interactions between these Transcription Factors, proposing that TF binding sites should be analyzed combinatorially.

Major comments:

One of the goals for this study is to increase the experimental throughput for studying enhancers. It is great to see many groups working on addressing this problem from different perspectives. Using QeFS, many barcoded enhancer lines can be integrated and tested directly in the Drosophila genome (and not as episomes) and screened based on RNA-Seq levels. Compared to eFS, QeFS allows the quantification of RNA levels and has an advantage over cell populations. The enhancer readout is now in the proper cellular context, and entire fields of cells could be quantified and expression patterns observed. Additionally, QeFS was used to explore how four TFs that are not well characterized control mesodermal development.

YES. THANK YOU.

However, recent studies have screened much more than 88 enhancer lines using standard techniques (i.e., insertion and antibody staining / in-situ hybridization) (Kvon et al., Cell 2020 and Le Poul et al., Science Advances 2020) or other higher-throughput techniques (Fuqua et al., 2020). These studies additionally provide patterning information in addition to enhancer activity levels. From this perspective, the advantage QeFS has over other techniques is the RNA quantification, which is much more quantitative than the other methods introduced. I believe the "high-throughput" component of QeFS is plausible but not demonstrated in this manuscript and should be toned-down.

We appreciate the honest feedback and apologize for any salesmanship that may have crept into our writing. We have modified our text accordingly.

I was disappointed to see that the authors do not test the patterning of these enhancers. In my opinion, there is not as much insight gained from doing QeFS compared to standard techniques or using cell lines. I believe the study would become much more informative, strengthen its argument if the expression patterns were imaged - perhaps a subset from each epistatic category (synergistic, alleviating complementary, alleviating complete, and sign epistasis). This way, the authors could relate the mutant phenotypes to see why these different forms of epistasis are occurring.
Based on the current data, I found the epistasis argument hard to just. For example, it could be possible to have variable effects of modifying one site that would be cell-type specific in the embryo. At the same time, the other "epistatic" site may have variable effects in other cell-types. It would benefit from having staining for the inputs -- what cells are they expressed - and a subset of their collection imaged to explore the effects of the mutations. This approach would more directly address epistasis by controlling for cell-type-specific changes, pleiotropy, etc.

Overall, it is clear that the authors put much work into this technique and were diligent with their statistics and data analysis. The technique, QeFS, is an improvement upon a previous protocol, and it was used to identify TF interactions, which is (in my opinion) the more important part of this story. However, it would benefit from more imaging to of (at least) a subset of the reporter constructs from each category to potentially understand why the different forms of epistasis are observed.

We appreciate the suggestion. We have generated new imaging data for 20 enhancers (15 mutants and 5 corresponding wild type) for which transformant lines were available. (We apologize that the generation of new lines is beyond the current scope of this project.) Surprisingly, very few of these mutations (now highlighted in the main body text) appear to have cell type-specific effects on expression. Representative images are now presented in Figures S16-S20 that we have added in the revised manuscript.

Minor comments:

"Such epistatic interactions, where the regulatory output of a particular combination of motifs is different from that expected if the motifs are treated as acting independently, is important to identify since rules learned for individual motifs in one enhancer context might not be informative of those motifs' regulatory consequences in a different enhancer context." - This sentence is convoluted. Consider rewriting or splitting it into two.

We agree, and the grammatical error that snuck through didn't help readability either. We have lightly edited this sentence.

"Massively parallel reporter assays (MPRAs) [21] and STARR-Seq [22] are powerful in their ability to assay tens to hundreds of thousands of short elements (typically less than 200 bp in length) and for their ability to drive expression of a reporter in cultured cells."

"To assay cis-regulatory elements for enhancer activity in a quantitative, highly parallel fashion in a chromosomal context in the context of particular tissues in whole Drosophila embryos, we developed quantitative enhancer-FACS-Seq (QeFS). In QeFS, each cis-regulatory element was cloned upstream of a reporter gene, where a in whose 3' UTR a degenerate sequence was inserted into the 3' UTR and used as an identifying tag for the RNA-Seq readout of enhancer activity (Additional file 1: Fig. S1, S2, S3)."

It would be great for the authors to add a comment on how the effects may extend to the native locus.

While we obviously cannot know what effect precise genome editing of native enhancers would have on endogenous gene expression without experiments that are well beyond the scope of this study, we do point out on page 16 that "Notably, a prior study found that the chromatin accessibility profiles, as determined by ATAC-Seq, of genomically integrated reporters are the same as those of the endogenous enhancers [51], suggesting that this strategy is likely to measure activity in the appropriate chromatin context."

The authors should comment on how their results compare to the recent work by Fuqua et al. (Nature, 2020)?

We have briefly highlighted the contrasting results and speculated as to which differences between our study systems might be significant on page 16.

In figure 3C, this is hard to interpret. It would be great if the authors could note what enhancer they came from - color code?

We have added information to the relevant figure legend describing the relationship between the order in which enhancers are shown and the data points in what are now shown in Figure 4 A and B.

**Second round of review**

**Reviewer 1**

The authors have responded to our concerns with additional analyses and figure panels and with changes to the text. However, our original concern regarding the anecdotal nature of the observations remains valid and the main claims of the manuscript are still not supported by the data. Moreover, some of the newly added information suggests that the method is not very reproducible such that its quantitativeness is questionable. In light of this, we cannot recommend publication of this manuscript.

1. Reproducibility and quantitativeness of the data, data display and statistical analysis
The display and statistical evaluation of the data is inadequate and does not allow the assessment of experimental reproducibility. Moreover, we are concerned that the effect sizes are low and the variance between replicates are high. These concerns are based on several shortcomings of the data presentation:
- Figure 3 uses an inadequate color scale: much of the color scale is dedicated towards assessing gain of activity (values >1, bright green and yellow) when in fact loss of activity is more important. However, values that indicate loss of activity (<1, dark green and blue) are compressed into a very narrow spectrum that does not really allow the assessment of change. Such fold-changes must be assessed on a log scale and a color scale that allows the clear distinction of no change (e.g. white), upregulation and downregulation.
- Many of the statistical tests are performed with a rank-based statistics (Conover-Iman test) when quantitative changes should be assessed. Moreover, very lenient thresholds of 0.1 after Benjamini-Hochberg correction are partly used (e.g. in Fig. 3)
- Throughout the manuscript, error bars are now defined as requested yet represent SEM without stating N. As SEM is scaled down SD (SEM=SD/square root (N)), SEM without N does not allow the assessment of variability. Moreover, provided the size of the error bars (e.g. in Figures S21-32), the variance is likely very high, suggesting poor reproducibility.
- Fig. S21-32 that display 'absolute expression measurements' also indicate that QeFS has a very low dynamic range and that different tests show very small changes with high variance (note that s.e.m. values are shown!). Furthermore, the y-axis values suggest that the on-state of some enhancers are less active compared to the mutant or off state of some other enhancers or that QeFS is not quantitative as claimed. These newly added figures confirm our original major concern 2 regarding quantitativeness of method and reported changes.

2. Strong claims based on anecdotal data (original concerns 3 and 4)
Even though the authors acknowledged that their data regarding the effects of motif mutation is anecdotal (see their responses to concerns 3 & 4), they are repeating the same claims based on the same data with only minor modifications. The abstract still reads "Deaf1 and CG12236-PB sites lead to context-dependent increases, while Schlank and ZIPIC sites lead to context-dependent decreases, in mesodermal enhancer activity." And on page 12 for example, the authors state "Loss of Deaf1 or CG12236-PB sites causes context-dependent reduction in mesodermal enhancer activity" and "Loss of Deaf1 sites typically led to decreased enhancer activity in whole mesoderm, with significant reduction in 3/9 enhancers". While the latter statement now correctly indicates that the desired result is only obtained in 3 out of 9 cases, it is still presented as a typical (!) change, even though a trend observed in only 3 out of 9 cases is rarely typical. The same concern is true for other examples of motif mutagenesis.

**Reviewer 2**

The authors have addressed my concerns and have strengthened the text and overall conclusions.

**Additional** **Reviewer 3**

My postdoc have now gone through the paper, Reviewer #1’s comments, and the authors’ response. We generally agree with the points originally raised by Reviewer #1, but we think they have been adequately addressed during revision. Specifically:

1.            Reproducibility and quantitativeness: new Figure 3 shows good reproducibility between two replicate datasets. We agree using a log scale is more appropriate but still think Figure 3 could be made more accessible overall, especially by labelling the axes and maybe using less obscure notation.

2.            Statistical tests: we can’t comment on whether the statistical tests used are appropriate in this context or not, but they seem consistent with published literature. Replicate numbers are correctly reported.

3.            “Anecdotal” data: the revised version has clarified the extent to which the observations are detected in one case or in a significant proportion of the enhancers. We think this sufficiently addresses the points raised by the reviewers, but we further encourage the clarifications proposed in the response for a further revised version. We also think that explicitly stating what the authors mean by “context dependent” (e.g., context within the enhancer or around the enhancer) would help clarify the relevance of the anecdotal data. Further, and probably beyond the specific charge you gave us, we think that the proof-of-concept of the technology is exciting and timely beyond any conclusions about the role of specific transcription factors.

**Authors’ response to reviewers**

We have made a number of changes in response to editorial and referee comments, highlighted here. Comments from Referee 3 are in blue.

- The Abstract has been shortened to 100 words to conform with the specification for a Method article at the editors' suggestion. The new abstract reads: "Understanding the contributions of transcription factor DNA binding sites to transcriptional enhancers is a significant challenge. We developed Quantitative enhancer-FACS-Seq for highly parallel quantification of enhancer activities from a genomically integrated reporter in Drosophila melanogaster embryos. We investigated the contributions of the DNA binding motifs of four poorly characterized TFs to the activities of twelve embryonic mesodermal enhancers. We measured quantitative changes in enhancer activity, and discovered a range of epistatic interactions among the motifs, both synergistic and alleviating. We find that understanding the regulatory consequences of TF binding motifs requires that they be investigated in combination across enhancer contexts."

We generally agree with the points originally raised by Reviewer #1, but we think they have been adequately addressed during revision. Specifically:

1. Reproducibility and quantitativeness: new Figure 3 shows good reproducibility between two replicate datasets. We agree using a log scale is more appropriate but still think Figure 3 could be made more accessible overall, especially by labelling the axes and maybe using less obscure notation.

- Figure 3 was replaced with the new version submitted with our rebuttal letter, in which up- and down-regulation of enhancer activity are represented by red and blue colors, respectively, and the color key is labelled with log_2_ fold-change. Labelling of the color key was also expanded, and the figure legend made more detailed, in response to the third referee's request for improved labelling.

2. Statistical tests: we can’t comment on whether the statistical tests used are appropriate in this context or not, but they seem consistent with published literature. Replicate numbers are correctly reported.

3. “Anecdotal” data: the revised version has clarified the extent to which the observations are detected in one case or in a significant proportion of the enhancers. We think this sufficiently addresses the points raised by the reviewers, but we further encourage the clarifications proposed in the response for a further revised version. We also think that explicitly stating what the authors mean by “context dependent” (e.g., context within the enhancer or around the enhancer) would help clarify the relevance of the anecdotal data. Further, and probably beyond the specific charge you gave us, we think that the proof-of-concept of the technology is exciting and timely beyond any conclusions about the role of specific transcription factors.

- "Loss of Deaf1 sites typically led to decreased enhancer activity in whole mesoderm" on p. 10 was changed to "We observed that loss of Deaf1 sites led to decreased enhancer activity in whole mesoderm when it had any reliably detectable effect" for clarity, as discussed.
- A new passage has been added to the Discussion on p. 15 to incorporate discussion points from our rebuttal letter: "Notably, all 4 motifs that we investigated in this study are those of poorly characterized regulators. Most TFs that have strong effects have in all likelihood already been identified from earlier genetic studies and screens in the literature; many poorly characterized TFs remain, and they likely are regulators that exert effects that are subtle and/or highly context-dependent in a manner that depends on the particular enhancer sequence and/or the particular tissue or cell type in which activity is being assessed. Examining the effects of these motifs in combination and in the context of several different enhancer sequences (all assayed at a common genomic location) in the *Drosophila* mesoderm permitted the detection of such subtle effects."
- The following sentence was added to the Discussion on p. 16: "In all cases, the effects of mutating binding sites of a TF were highly context-dependent, in that effects varied across different wild type enhancers and when different combinations of additional motifs were also mutated."
